# Malady in motion: Effects of chronic wasting disease on host movement

**DOI:** 10.1002/eap.70338

**Published:** 2026-10-23

**Authors:** Marie L. J. Gilbertson, Daniel P. Walsh, Daniel J. Storm, Allen Herbst, Wendy C. Turner

**Affiliations:** ^1^ Wisconsin Cooperative Wildlife Research Unit, Department of Forest and Wildlife Ecology University of Wisconsin‐Madison Madison Wisconsin USA; ^2^ U.S. Geological Survey, Montana Cooperative Wildlife Research Unit University of Montana Missoula Montana USA; ^3^ Wisconsin Department of Natural Resources Eau Claire Wisconsin USA; ^4^ U.S. Geological Survey National Wildlife Health Center Madison Wisconsin USA; ^5^ U.S. Geological Survey, Wisconsin Cooperative Wildlife Research Unit, Department of Forest and Wildlife Ecology University of Wisconsin‐Madison Madison Wisconsin USA

**Keywords:** case–control study, conditional logistic regression, disease ecology, disease‐induced behavior change, harvest susceptibility, movement ecology, piecewise regression, *PRNP* genotype, sickness behavior, wildlife health

## Abstract

Pathogens can have significant impacts on host movement and behavior with consequences for ongoing transmission. For example, chronic wasting disease (CWD) causes severe nutritional deficiencies and neurologic changes, which may alter white‐tailed deer (*Odocoileus virginianus*) movement and subsequent transmission risk. Our objectives were to test for changes in movement and habitat selection in deer affected by CWD, and determine the timing of any changes we observed. We leveraged comprehensive movement and mortality data from a large study of free‐ranging deer in southwest Wisconsin, a region with high prevalence of CWD. We used necropsy data to identify end stage CWD cases with movement data leading up to death (*n* = 44), and sex‐, date‐, and age‐matched test‐negative controls. Using GPS collar data, we identified extra‐home range excursions and tested for differences in excursion rates, distances, and durations between cases and controls. We evaluated movement characteristics (e.g., movement rates, weekly range sizes) and habitat selection for the two groups, using change point analysis to determine when behavior changes occurred during disease progression. Excursion distances were reduced for CWD cases but were otherwise largely unaffected by infection status. However, CWD cases moved at slower rates and maintained smaller ranges than controls. Habitat selection varied minimally between cases and controls, though CWD cases had an increased preference for proximity to forest edges. While changes in case movement rates and range sizes were detected at or before 6 months prior to death, other movement and habitat selection changes appeared about 2 months prior to death. In addition, CWD cases were frequently in good body condition even within 6 months prior to death, implying that clinically apparent changes occur very late in the disease progression. Our results suggest the occurrence of subclinical and clinical phases of infection, beginning around 6 and 2 months prior to death, respectively. Public communication may therefore benefit from emphasizing that CWD‐infected deer are infectious long before they appear sick. Further, because most movement changes occur for very short periods relative to the infectious period of CWD, typical healthy deer movement is likely adequate for representing transmission dynamics for surveillance and management planning.

## INTRODUCTION

Animal behavior shapes pathogen transmission through heterogeneity in direct or indirect contact patterns, yet infectious disease can also alter host behavior and subsequent transmission. Furious rabies, for example, increases an infected animal's risk of biting and transmitting to other individuals (Brookes et al., 2019; Reynolds et al., 2015). Infection with *Toxoplasma gondii* can induce changes in rodent behavior that increase their susceptibility to predation by feline definitive hosts (Berdoy et al., 2000; Vyas et al., 2007). Sickness behavior, in contrast, can include prolonged periods of rest and reduced contact rates, thereby reducing the risk of onward transmission (Adelman & Martin, 2009; Lopes et al., 2016). Infection can also alter host foraging behavior and habitat selection (Grabow et al., 2024; Tillman & Adelman, 2023), which can in turn alter the locations at which individuals are most likely to transmit to others. Disease‐induced behavior changes can therefore have critical impacts on disease dynamics.

Such behavior alterations may be most readily observable for acute infections, where changes occur relatively abruptly. For chronic or subclinical infections—especially in difficult to observe wildlife—detecting disease‐induced behavior changes can be challenging (Grabow et al., 2024). Periods of wildlife monitoring (e.g., through GPS tracking devices) are often limited in duration, making slow or gradual shifts in movement or behavior difficult to capture, particularly against the backdrop of normal seasonal variation in behavior (Adelman et al., 2014; Krause et al., 2013). Yet chronic diseases are known to cause progressive degeneration in wildlife hosts that may alter broader transmission dynamics. For example, progressive infection with feline immunodeficiency virus (FIV) in African lions (*Panthera leo*) was associated with dehydration, likely as a result of general depression or malaise (i.e., sickness behavior; Roelke et al., 2009). Theoretical work suggests that when infection lowers contact rates, consequent reductions in transmission may be most significant for low transmissibility pathogens like many chronic wildlife diseases (White et al., 2018). However, these effects will be shaped by temporal lags between the onset of infectiousness (the latent period) and the onset of behavioral changes (the incubation period). A short latent period coupled with a long incubation period can yield ample opportunity for transmission prior to the onset of late‐stage sickness behaviors.

Chronic wasting disease (CWD) is an important contemporary example of a chronic wildlife disease with a long incubation period and eventual behavior changes of unclear transmission and management relevance. Affecting cervid species, CWD is transmitted both directly and through the environment, and is always fatal. The incubation period in white‐tailed deer (WTD) (*Odocoileus virginianus*) is thought to range from 16 to 48 months, though the duration of this period is uncertain for free‐ranging deer. Further, deer prion protein gene (PRNP) polymorphisms can affect CWD progression and the duration of the incubation period (Haley et al., 2019; Johnson et al., 2011; Ketz et al., 2019; Mathiason et al., 2009; Robinson et al., 2012; Williams, 2005; Williams & Miller, 2002). For example, in one experimental study of orally inoculated WTD, a single allele change from the “wild‐type” glycine to a serine residue at PRNP codon 96 was associated with incubation periods even longer than 48 months (from 693 ± 27 days in 96GG deer to 956 ± 107 days in 96GS deer; Johnson et al., 2011).

When clinical symptoms appear, captive deer demonstrate many behavior changes that could alter contact rates, space use, and consequent transmission potential, with death occurring weeks to months after the onset of clinical signs. These clinical effects (Mathiason et al., 2009; Williams, 2005; Williams et al., 2002; Williams & Miller, 2002) include wasting and polydipsia (increased thirst), which could alter habitat selection and consequent environmental contamination if, for example, affected animals prioritize proximity to water sources; and lethargy and ataxia, which could reduce movement and contact rates or ranging behavior, and thereby reduce transmission potential. Some evidence indicates CWD infection is associated with behavior changes in free‐ranging deer, with reduced movement rates and changes in habitat selection detected within 6 months prior to death among mule deer (*Odocoileus hemionus*) in Wyoming (Barrile et al., 2024). These CWD‐infected mule deer also exhibited some increased preference for proximity to water, which could be associated with disease‐induced polydipsia (Barrile et al., 2024). Similarly, Wyoming WTD with CWD reduced their activity and range sizes within about 4 months prior to death, though the sample size for the timing of these changes was limited (*n* = 4; Edmunds et al., 2018). The timing of behavior changes in CWD‐infected WTD (or deer hereafter) is highly uncertain. Further, as habitat generalists, WTD space use and habitat selection can be highly variable across regions (Sample et al., 2023; Walter et al., 2009), yielding uncertainty in how deer behavior may change in response to CWD status outside the arid habitats of the mountain west. These knowledge gaps are critical for understanding if CWD‐induced behavior changes have an impact on subsequent transmission.

In contrast to movement rates, dispersal and migration do not seem to be affected by CWD status (Barrile et al., 2024; Edmunds et al., 2018), so the potential for CWD to spread to new areas would not appear to be reduced as disease progresses. However, dispersal primarily occurs among juveniles, which have a lower risk of being CWD‐positive in the early stages of a CWD outbreak (Gilbertson, Ketz, et al., 2022; Jennelle et al., 2014). Further, occurrence of migration in WTD is variable between populations; many do not exhibit migration at all, and among those that do, migration rates are generally affected by winter severity (Fieberg et al., 2008; Morrison et al., 2024). Of perhaps more general relevance to the long‐distance spread of CWD are extra‐home range excursions (hereafter, *excursions*): short‐term (typically days) movements outside the home range—sometimes at great distances—with a return to the normal, stable range (Hooven et al., 2023). These under‐studied movements are relatively common (Hooven et al., 2023) and can provide short duration but significant opportunities for pathogen spread (O'Brien et al., 2014). Importantly, modeling and surveillance efforts are typically based on the assumption that CWD positive deer do not significantly modify their movement or habitat selection, and may ignore excursions because they are poorly characterized in empirical systems. Violations of these assumptions could produce poor predictions of geographic spread or high‐risk habitats, resulting in reduced efficacy or misapplication of management efforts.

In light of these knowledge gaps, our objective was to determine if and when CWD infection altered WTD movement and habitat selection in a high‐prevalence region of the upper Midwest: Wisconsin, USA, where CWD prevalence can exceed 40% (Wisconsin Department of Natural Resources, 2022). To help frame these questions within the context of the apparent clinical status of deer, we also aimed to determine if changes in body condition were concurrent with changes in movement behavior. We expected CWD infection would be associated with reduced daily activity, increased tortuosity of movement (i.e., less direct), and increased selection for high‐quality forage habitats or proximity to water. Further, we expected most behavior changes would occur about 2–4 months prior to death, based on captive studies and previous studies in WTD and mule deer in Wyoming. We hypothesized that changes associated with severe clinical disease (e.g., reduced body condition, more tortuous movements, polydipsia and increased proximity to water) would occur later in the disease course than non‐specific changes (e.g., reduced movement rates). Similarly, we expected changes in excursion behavior would occur closer to death when clinical symptoms are advanced but considered either direction of change (i.e., increased or decreased excursions) plausible. Excursions could increase due to searching for resources or reduced site fidelity secondary to impaired awareness or cognition; alternatively, excursions could be decreased due to reduced energy associated with advanced wasting. Results of this study shed light on the transmission and management relevance of chronic infection–induced behavior changes in an economically important free‐ranging wildlife species.

## METHODS

### Deer captures, CWD testing, mortality investigations

As part of ongoing CWD research, the Wisconsin Department of Natural Resources (WDNR) captured and GPS‐collared 763 WTD >8 months of age in southwest Wisconsin (Dane, Iowa, and Grant counties; Appendix S1: Figure S1) from 2017 to 2020. Detailed capture and processing methods are described in Gilbertson, Ketz, et al. (2022); all capture and handling efforts were approved under the Wisconsin Department of Natural Resources' Animal Care and Use Committee (Protocol: 16‐Storm‐01). At capture, deer body condition was recorded as integer values on a 0–10 scale based on palpation of fat along the lumbar spine to the tail base. If fat was palpable over about 50% of this extent, the deer was scored as a “5”; if fat was present everywhere along the lumbar spine and tail bed (i.e., the spine was not readily palpable), the deer received a maximum score of “10.” This approach was based on methods for deceased deer, which include measurements of rump fat (Kistner et al., 1980).

In addition to body condition, deer age was estimated at capture based on body size and tooth wear (juveniles and yearlings or subadults). For adults and individuals of uncertain age, an incisiform canine tooth was extracted for aging via cementum annuli (Storm et al., 2014). Ante‐mortem CWD status was determined using biopsy of rectoanal mucosa‐associated lymphoid tissue (RAMALT), and subsequent immunohistochemistry (IHC) performed by the Wisconsin Veterinary Diagnostic Laboratory, Madison, Wisconsin, USA (Gilbertson et al., 2024). Deer genotyping at *PRNP* codon 96 was performed by the U.S. Geological Survey National Wildlife Health Center through the University of Wisconsin Biotechnology Center sequencing core using standard methods for ear tissue (Johnson et al., 2003, 2006).

When GPS‐collared deer died, technicians completed mortality investigations, as described by Gilbertson, Brandell, et al. (2022). In brief, investigations included recording the geographic coordinates of mortality sites, post‐mortem CWD diagnostics (IHC of retropharyngeal lymph node or obex), and necropsy either in the field by technicians or at the University of Wisconsin School of Veterinary Medicine by veterinary pathologists. While body condition at capture was based on palpation, nutritional condition at death was based on the quantity or absence of subcutaneous fat and the amount of fat around internal organs (e.g., heart, kidneys). For consistency across field and laboratory necropsies, we summarized post‐mortem body condition into three categories for this study: “good” (abundant subcutaneous and/or internal body fat); “fair” (typically, limited subcutaneous body fat, but adequate internal body fat); or “poor” (little to no subcutaneous and internal body fat). Further, necropsies also recorded the presence of serous atrophy of bone marrow fat, which is an indicator of severe nutritional deprivation.

### Case–control selection

Deer captures and post‐mortem investigations yielded a set of 245 GPS‐collared deer of known post‐mortem CWD status from which to retrospectively identify cases and matched controls. Cases were defined as deer that tested CWD‐positive at death. Furthermore, to maximize our ability to detect changes in behavior associated with CWD, cases were defined as in putative “end stage” disease at death, based on being in poor body condition with evidence of bone marrow fat atrophy. Controls were CWD‐negative at death. For cases, we followed Barrile et al. (2024) in extracting up to 6 months of GPS collar data prior to death for evaluation. This time frame was based on our expectation that most behavior changes would occur about 2–4 months prior to death, in keeping with observations from captive deer (Williams, 2005; Williams et al., 2002; Williams & Miller, 2002) and free‐ranging mule and WTD in Wyoming (Barrile et al., 2024; Edmunds et al., 2018). Our objective was to compare CWD‐positive deer movement to typical deer movement, so control movement data were extracted based on temporal matching to cases, rather than specifically from the months prior to control death.

Each case was matched to a single control individual to control for influences on habitat selection and movement aside from disease status. While higher ratios of controls to cases are preferable, we were limited by the number of CWD‐negative individuals that could appropriately match to cases. All case–control pairs were of matching sex, and we temporally aligned pairs by matching to day of the year (i.e., year could differ within pairs) to control for seasonal variation in deer movement and habitat selection (Gilbertson et al., 2025). We attempted to match case–control ages, based on case age at time of death. However, because prior analysis with this dataset found limited influence of age on seasonal habitat selection (Gilbertson et al., 2025), this criterion was lower priority than sex and day.

Hand‐picking case–control matches quickly becomes impractical and inefficient as sample sizes and matching criteria increase, so we used a simulation approach to reproducibly automate selection of an optimal set of case–control matches. In each of 500 simulations, we randomly ordered cases, and then selected a candidate control for each case based on sex‐, day‐ (maximize the number of overlapping days between case and control, ignoring year), and age‐matching (minimize the difference in age between case at death and control at the same time of year). Because some cases had multiple potential controls meeting our matching criteria, each simulation could result in a unique set of case–control pairs with varying quality of matches. We selected the set of case–control matches that minimized unpaired individuals and poor quality matches (e.g., pairs with large age discrepancies or limited temporal overlap).

To determine if our analyses were robust to variable GPS collar fix rates (i.e., hours between successive GPS locations) within and between matches, we completed sensitivity analyses (Appendix S3). Based on the results of these analyses, we downsampled fixes within case–control matches as needed to yield fix rates that matched within pairs but could differ between pairs. The final set of matched, resampled data was used in all statistical models evaluating changes in condition and behavior associated with CWD (described below), unless otherwise specified; Figure 1 provides an overview of our research questions and associated statistical analyses (additional details and sample sizes in Appendix S1: Table S1).

**FIGURE 1:**
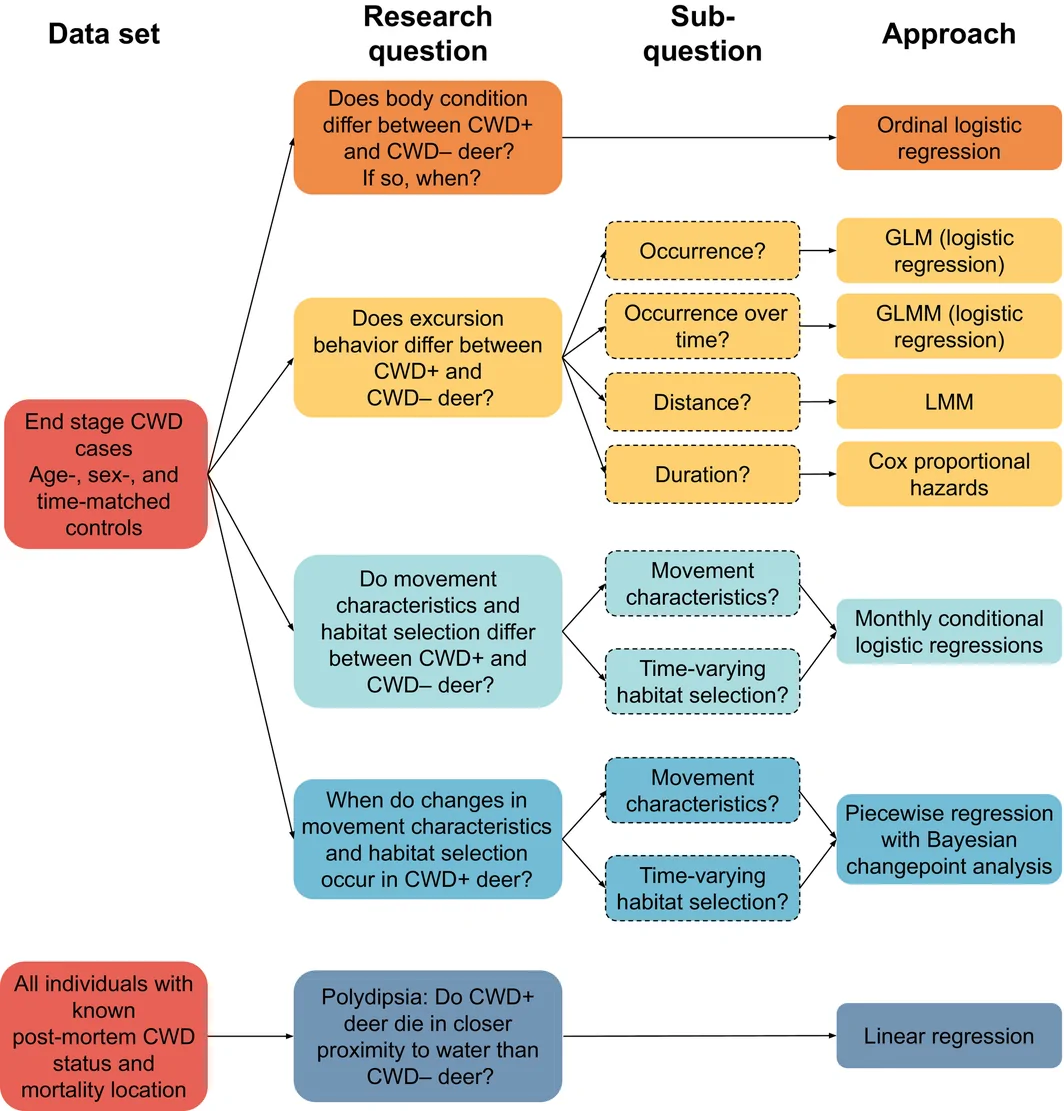
Flowchart of methods used to evaluate changes in condition, movement, and habitat selection associated with chronic wasting disease (CWD) in white‐tailed deer (*Odocoileus virginianus*) in southwest Wisconsin, USA. Sets of CWD cases and controls used to address each research question are given in red boxes; each individual research question, accompanying sub‐questions (dashed outline), and modeling approach is given by a different color. GLM, generalized linear model; GLMM, generalized linear mixed model; LMM, linear mixed model; polydipsia, increased thirst.

### CWD and body condition at deer capture

We tested for differences between cases and controls in body condition at capture. Deer were captured at variable times prior to death, but, per our case definition above, all cases were in poor condition when they died. This analysis therefore aimed to determine if and when changes in body condition associated with CWD were identifiable prior to death. We expected cases would be in worse body condition at capture than controls, especially when captured closer in time to their eventual death.

Body condition at capture was inherently ordinal (0–10 integer score), so we fit cumulative logistic regression models, assuming proportional odds. The response variable was the integer body condition score at capture, and predictors included hypothesis‐driven combinations of CWD status, days prior to death (scaled and centered), age class (juvenile, subadult, adult), sex, and *PRNP* codon 96 genotype (96GG, 96GS, 96SS). In addition, we included an interaction between CWD status and days prior to death. Model selection was based on Akaike information criterion (AIC; Akaike, 1974; Appendix S2: Table S1 for candidate models).

### Extra‐home range excursions

We identified excursions based on daily mean net‐squared displacement (NSD) values associated with each deer's GPS locations. By comparing a given GPS location to the trajectory's origin point, unusually or abruptly increased NSD values can be indicative of extra‐home range movements. Using daily mean NSD values helped reduce rates of false excursion detections. We used the *anomalize* R package (Dancho & Vaughan, 2023) to identify “anomalous” NSD values. The package uses seasonal decomposition of a time series by Loess (Cleveland et al., 1990) to remove seasonal and trend components from a time series, leaving only “remainders.” Anomalies are those observations that exceed three times above or below the interquartile range of the remainders. Following initial anomaly detection with *anomalize*, we visually screened all identified anomalies, including events clearly outside an individual's normal range extent and not regularly revisited. This approach may fail to detect especially short distance excursions, but as our primary interest is in excursions that could allow CWD shedding or transmission in novel areas outside a deer's home range, we deemed this an acceptable limitation.

We expected excursions could be more common around the time an individual dispersed, but the process driving this behavior—which is performed almost exclusively by juvenile or subadult males in our study area (Gilbertson, Ketz, et al., 2022)—would be fundamentally different from that driving excursions more generally. Therefore, for individuals known to have dispersed (Gilbertson, Ketz, et al., 2022), we excluded the month in which their dispersal occurred from the excursion analysis. Dispersals among this population are typically short duration (median 1.71 days; Gilbertson, Ketz, et al., 2022), so excluding a full month was conservative.

We tested if cases were more likely than controls to make excursions using logistic regression. We fit an “overall” model (i.e., did an individual ever make an excursion?), and a “monthly” model (i.e., did the probability of making an excursion change as cases approached death?). For all models, predictors were combinations of CWD status, sex, the number of days observed (scaled and centered; also tested as a second order polynomial), and the fix rate (scaled and centered). For the monthly model, we fit a binomial generalized linear mixed model that added month prior to case death as an interaction with CWD status, and random intercepts for individual. Model selection was based on AIC (Appendix S2: Tables S3 and S5 for suites of candidate models); models within ∆2 AIC were considered equivalent and model assumptions were checked with binned residuals plots (overall models; arm package; Gelman & Su, 2024) or simulated residuals (monthly models; DHARMa package; Hartig, 2022).

We also tested if cases traveled farther during excursions than controls. We defined excursion distance as the maximum distance between excursion locations and that month's range centroid, and log‐transformed this value for use as the response variable in statistical models. We used linear mixed‐effects models to investigate this question, with predictors including hypothesis‐driven combinations of CWD status, months prior to case death, sex, and season in which an excursion started (spring/early summer, fall/breeding, or winter/non‐breeding), with random intercepts for each individual. We also tested two‐way interactions between CWD status, months prior to case death, and sex. Model selection was based on AIC and prioritizing parsimony among equivalent models (those within ∆2 AIC; Appendix S2: Table S7 for candidate models).

Among excursions, we tested if CWD cases made longer duration excursions than controls. We used techniques that account for interval censoring commonly applied in survival modeling (Fay & Shaw, 2010), where the actual excursion duration was unobserved but fell between known minimum and maximum durations. The minimum duration was the time difference between the first and last anomalous NSD observations in a given excursion event. The maximum duration was the time between the last and first non‐anomalous NSD observations preceding and following an excursion event, respectively. We fit a semiparametric Cox proportional hazards (PH) model with an interval response for excursion duration (Anderson‐Bergman, 2017; Pan, 1999; Wellner & Zhan, 1997). We followed the recommendations from Kleinbaum and Klein (2011) for checking the PH assumption by visually assessing observed (from Kaplan–Meier curves) versus expected (from PH models) plots for each candidate predictor. Based on this evaluation, our final model included CWD status and hours between fixes as predictors.

### CWD and deer movement characteristics

We tested for differences in movement characteristics between CWD cases and controls following the approach by Barrile et al. (2024). We calculated six movement characteristics, primarily using daily values to reduce temporal autocorrelation: (1) daily mean movement rate (in kilometers per hour), (2) daily variance in movement rate, (3) daily distance traveled (summed step lengths), (4) daily tortuosity (summed step lengths/distance between start and end points for a day), (5) daily mean displacement from monthly range center (range center allowed to update each month prior to case death), and (6) weekly occurrence distribution area (in square kilometers; hereafter, weekly range area).

For daily displacement, the monthly range center was determined by extracting the centroid from the 50% isopleth of an individual's monthly occurrence distribution, as estimated using Brownian bridge continuous time movement models (Calabrese et al., 2016; Fleming et al., 2015). We elected to use the 50% isopleth so the centroid would be most representative of an individual's “core area” during a given month. However, if an individual had fewer than 30 locations in a given month, we used the median *x*‐ and *y*‐coordinates as the centroid for that month. The weekly range area was similarly based on occurrence distributions estimated using Brownian Bridge continuous time movement models. However, the weekly range area used the 95% isopleth and was only estimated if an individual had recorded at least six locations in a given week.

We used conditional logistic regression to identify differences in movement characteristics between cases and controls, fitting a separate model for each month prior to case death as in Barrile et al. (2024). The response variable was a binary 0/1 representing controls versus cases, respectively. Predictors were the scaled and centered movement characteristics. We note this model structure was fundamentally testing if movement characteristics were associated with or predictive of CWD disease status, rather than testing for the effect of CWD on individual movement characteristics (Barrile et al., 2024). If two metrics were correlated (Pearson ⍴ >0.6), we fit univariate models and used AIC to determine which correlated metric to include in the full model (Appendix S1: Table S2). In addition, we log‐transformed daily mean displacement from range center. Weekly range areas were evaluated in a separate model, as this metric was at a different temporal scale from the other metrics and was correlated with displacement from range center. The final daily model predictors were daily mean movement rate, daily tortuosity, and log‐transformed daily mean displacement from range center. We only included observations from a case–control match if both individuals had data for that day (or week for weekly ranges). Models were fit using the *survival* R package (R version 4.4.0; R Core Team, 2024; Therneau, 2024; Therneau & Grambsch, 2000), with “day” as the strata, and data clustered by case–control matches (i.e., cases were specifically evaluated against their matched control). Our sample sizes were limited, but we also post hoc divided our data set by case genotype at *PRNP* codon 96, and re‐ran our daily models. This analysis aimed to determine if some case genotypes were more susceptible to changes in movement prior to CWD‐associated death.

### CWD and deer habitat selection

We tested for differences in habitat selection between cases and controls. Typical habitat selection analyses provide one selection estimate for a static period of time (e.g., one season) for each habitat covariate. However, more frequent selection estimates would allow our analysis of habitat selection to mirror that for movement characteristics. We therefore followed methods described by Dejeante et al. (2024) to fit a dynamic logistic model for time‐varying habitat selection analysis for each deer. This analysis provides selection estimates associated with each GPS location, which could then be summarized into daily mean selection estimates, as in our movement characteristics analysis.

Like typical resource selection functions, the response variable for time‐varying resource selection functions (tvRSFs) is a binary 0/1 for available versus used locations, and predictors are a suite of environmental layers. The tvRSF assumes a Markov chain covariance matrix, **Q**, with users providing one initial value used across all diagonal elements. Dejeante et al. (2024) refer to the diagonal value as *Q*, and this parameter affects the smoothness of the estimated selection time series.

Habitat selection analyses can be affected by GPS collar fix rates (i.e., through temporal autocorrelation), the number of random locations drawn per observed location, and the spatial extent from which those random locations are selected. In addition, tvRSFs can be affected by the initial value for *Q*. To ensure our results were robust to these parameterizations, we performed a sensitivity analysis (Appendix S3). Based on this analysis, we were confident that tvRSF estimates were robust to *Q* values ≥2, and were relatively insensitive to the number of random locations and potentially autocorrelated data (i.e., robust to using the full versus a dataset downsampled to reduce autocorrelation). We therefore fit tvRSFs with the full movement dataset, 100 random control points for each observed location, and an initial *Q* = 2 (recommended by Dejeante et al., 2024). Random control points were drawn from the 90% isopleth of each individual's occurrence distribution for the 6‐month observation period (as estimated using Brownian Bridge continuous time movement models; Calabrese et al., 2016), but we allowed this “available” space to change in response to range shift events (Appendix S1: Section S1).

Habitat predictors for tvRSFs included: (1) distance to the closest water source; (2) broad‐leaved deciduous forested habitat (hereafter, *forested habitat*; vs. *other*); (3) distance to the closest forest edge of any type (i.e., included coniferous forest, though this forest type is rare in our study area); and (4) aspect, broken down into eastness and northness (Appendix S1: Section S2). Used locations were primarily in forested habitat (68.9%); the next most commonly used habitat type was forage grassland, comprising only 10.9% of used locations (Appendix S1: Figure S2). This limited use of non‐forested habitat types precluded finer resolution habitat classifications than the “forest” versus “other” categories. Additional details and sources for all habitat data are given in Appendix S1.

We determined if habitat selection differed between cases and controls using the same conditional logistic regression approach as used with movement characteristics. Control versus case status was the binary response variable; predictors were the daily mean selection coefficients for each habitat covariate, as estimated by tvRSFs. As above, we fit one conditional logistic regression per month prior to case death. No habitat selection estimates were correlated with each other, so the conditional logistic regressions included all habitat covariates described above.

As a complement to our habitat selection analysis, we also investigated the proximity of deer mortality sites to water sources, hypothesizing that CWD‐induced polydipsia would result in CWD positive deer dying in closer proximity to water sources than test‐negative individuals. For this analysis, we broadened our inclusion criteria, evaluating any mortalities where post‐mortem CWD status and body condition were known. We determined the linear distance between the mortality site and the nearest water source and used linear regression to determine if CWD‐positive deer died in closer proximity to water sources than CWD‐negative deer. We square‐root transformed distances to water sources as the response variable. Predictors were post‐mortem CWD status, with an interaction with body condition at death (good, fair, or poor).

### Timing of behavioral changes

We determined the timing of changes in movement characteristics and habitat selection by performing piecewise regression with Bayesian change point analysis using the *mcp* R package (Lindeløv, 2020), as in Barrile et al. (2024). We fit models separately for cases and controls. For all models, the response variables were the movement characteristics and habitat selection estimates evaluated in the conditional logistic regressions (movement characteristics: movement rate, tortuosity, displacement from range center, weekly range area; habitat selection estimates for: distance to nearest water source, forested habitat, distance to nearest forest edge, eastness, northness). The supported model families in the *mcp* package are limited for continuous response variables, so we transformed movement characteristic variables to meet assumptions of normality (see Appendix S1: Table S4 for transformations).

For each response variable and CWD status combination, we fit two models corresponding to a null and alternative hypothesis: (1) no change in the response variable over time (the expected result for controls), and (2) gradual change (i.e., a “flat” response variable until a change point, followed by a gradual change through the remainder of the time series). We ran our models with uninformative priors for change points and with Gaussian priors where means were based on apparent change points from the conditional logistic regressions (SDs were set to 15 days; both uninformative and Gaussian priors were truncated to minimum and maximum times in the time series; see Appendix S1: Table S5 for all informative priors). We ran three Markov chain Monte Carlo (MCMC) chains with a 22,000 step burn‐in, followed by 10,000 draws from each chain. We performed model selection (i.e., selecting between null and alternative hypotheses) by comparing expected log pointwise predictive density (ELPD) and checking model convergence. Model checking included evaluating Pareto *k* estimates, verifying *R*‐hat values were <1.1, and visually reviewing posterior predictive curves and MCMC chains.

## RESULTS

We identified 44 deer (36 female, 8 male) that met our CWD case definition; sample sizes for each analysis could vary slightly from this number, depending on data coverage and availability (Appendix S1: Table S1). Given our focus on end stage disease, cases usually died as a direct effect of their CWD infection (i.e., starvation with or without pneumonia; Appendix S2: Figure S1). Case mortalities most frequently occurred in January through May and in August, with March having the highest number of case deaths (Figure 2). The mean estimated age at death for cases was 3 years (range: <1 year to >8 years; two were adults of unknown age). Among cases, 37 deer were genotype 96GG at *PRNP* codon 96, 7 were 96GS, and none were 96SS. In contrast, 13 controls were genotype 96GG, 27 were 96GS, and 4 were 96SS.

**FIGURE 2:**
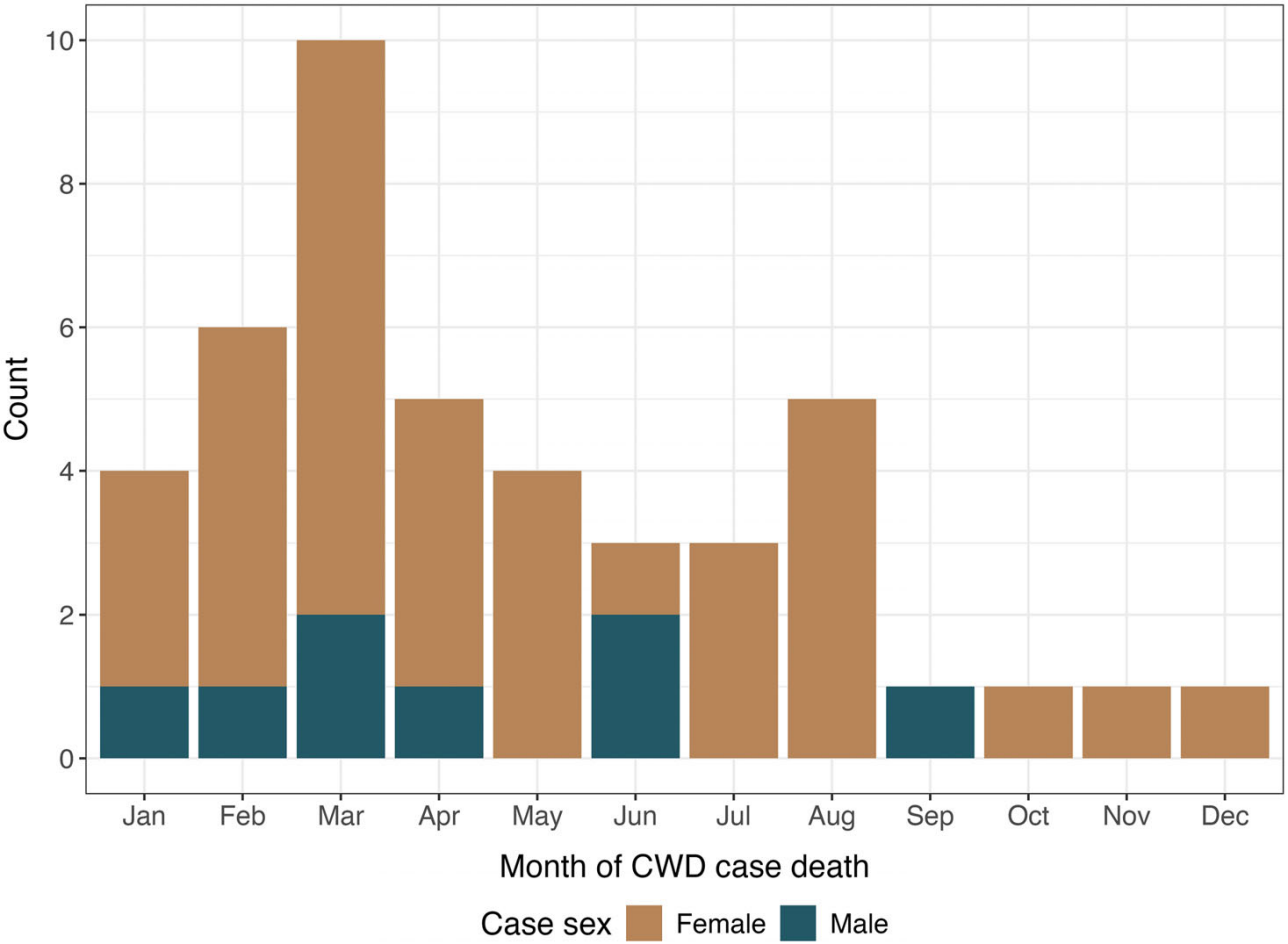
Month of end stage chronic wasting disease (CWD) case death among white‐tailed deer (*Odocoileus virginianus*) in southwest Wisconsin, USA. Bar colors correspond to deer sex, with females in brown and males in blue.

Following case–control matching, all pairs were sex‐matched. The mean age of controls was 3.9 years (range: <1 year to >15 years); the resulting median difference in age estimates within matches was 1.58 years (mean: 3.10 years; range: 0–13 years; Appendix S2: Figure S2). After resampling fix rates within matches, the mean number of days with observations (Appendix S2: Figure S3) was 123 for cases (range: 11–180 days) and 124 for controls (range: 30–181). Disregarding year, the mean number of matched days within a pair was 69 (range: 1–180 days). The mean fix rate after resampling was one GPS location every 9 h for cases and controls (case range: every 2–23 h; control range: every 2–26 h).

### CWD and body condition at deer capture

Body condition at capture was highly variable, and many CWD cases had excellent body condition scores within 6 months prior to their death, including those that were RAMALT positive at capture (Figure 3). We found no statistically significant relationship between body condition at capture and post‐mortem CWD status, while controlling for the days prior to death (Appendix S2: Tables S1 and S2). Age class was a significant predictor of body condition, with subadults and adults having higher scores than juveniles (subadults: odds = 0.21, 95% CI = 0.07–0.62; adults: odds = 0.18, 95% CI = 0.07–0.52; Appendix S2: Table S2, Figures S4 and S5).

**FIGURE 3:**
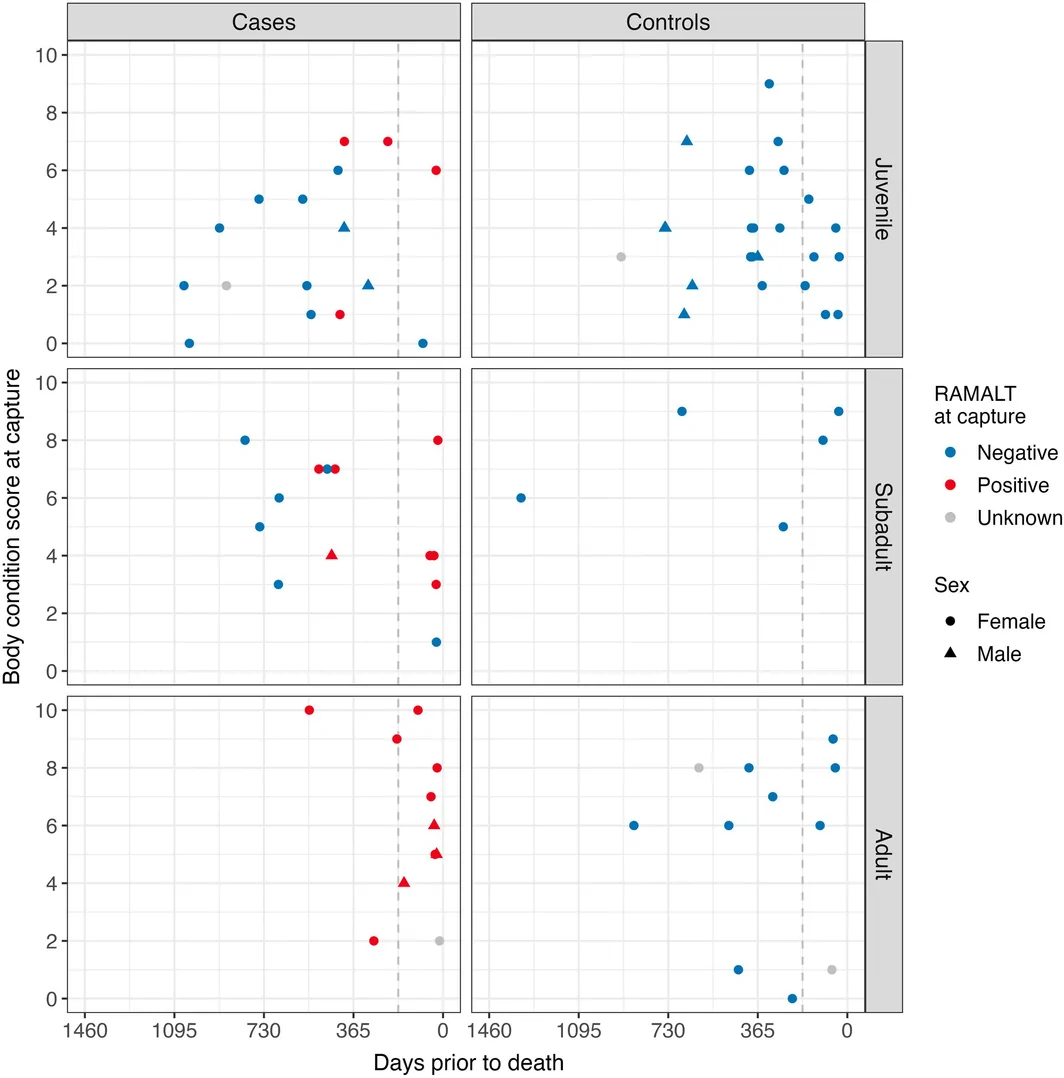
White‐tailed deer (*Odocoileus virginianus*) body condition scores at time of capture relative to time of death in southwest Wisconsin, USA. Body condition was scored as integers from 0 to 10, with 10 corresponding to excellent condition. The *x*‐axis gives the time between the capture event and an individual's later death (0 = time of death), and the vertical dashed line corresponds to 6 months prior to death. Points are colored by ante‐mortem chronic wasting disease (CWD) testing results (immunohistochemistry with rectoanal mucosa‐associated lymphoid tissue [RAMALT] biopsies) at the time of capture; at death, all cases were CWD positive and in poor body condition, and all controls were CWD negative. Point shapes correspond to deer sex, with females represented by circles and males by triangles. Panel columns are divided by post‐mortem CWD status, and panel rows by deer age class at capture (juvenile: ≤8 months old; subadult: ≤20 months old; adult: >20 months old).

### Extra‐home range excursions

We observed at least one excursion for 53 deer (28 deer had no observed excursions, and one individual's duration of observations was too short to detect excursions). The mean number of excursions per deer was 1.4 (range: 0–6). Controlling for variation in the duration of observations across deer, the mean excursion rate was 0.013 excursions per day observed (range: 0–0.097). The probability of a deer making at least one excursion prior to case death did not differ between cases and controls or between sexes, when evaluated across the whole 6‐month time series (odds ratio [OR] for cases: 0.93; 95% CI = 0.34–2.52; OR for males: 0.85; 95% CI = 0.21–3.89; Appendix S2: Tables S3 and S4), but did increase with increasing sampling effort (OR for number of days of observed, scaled, and centered: 1.91; 95% CI = 1.17–3.25; Appendix S2: Table S4). In our test of whether occurrence of excursions varied between cases or controls on a monthly basis, excursion probability increased for controls as the time series progressed (i.e., as months prior to case death decreased), but remained relatively constant for cases (OR for months prior to death for controls: 0.76, 95% CI = 0.61–0.95; Figure 4; Appendix S2: Tables S5 and S6). The number of days observed was no longer statistically significant in this model, and excursion probabilities again did not differ by sex (Appendix S2: Table S6).

**FIGURE 4:**
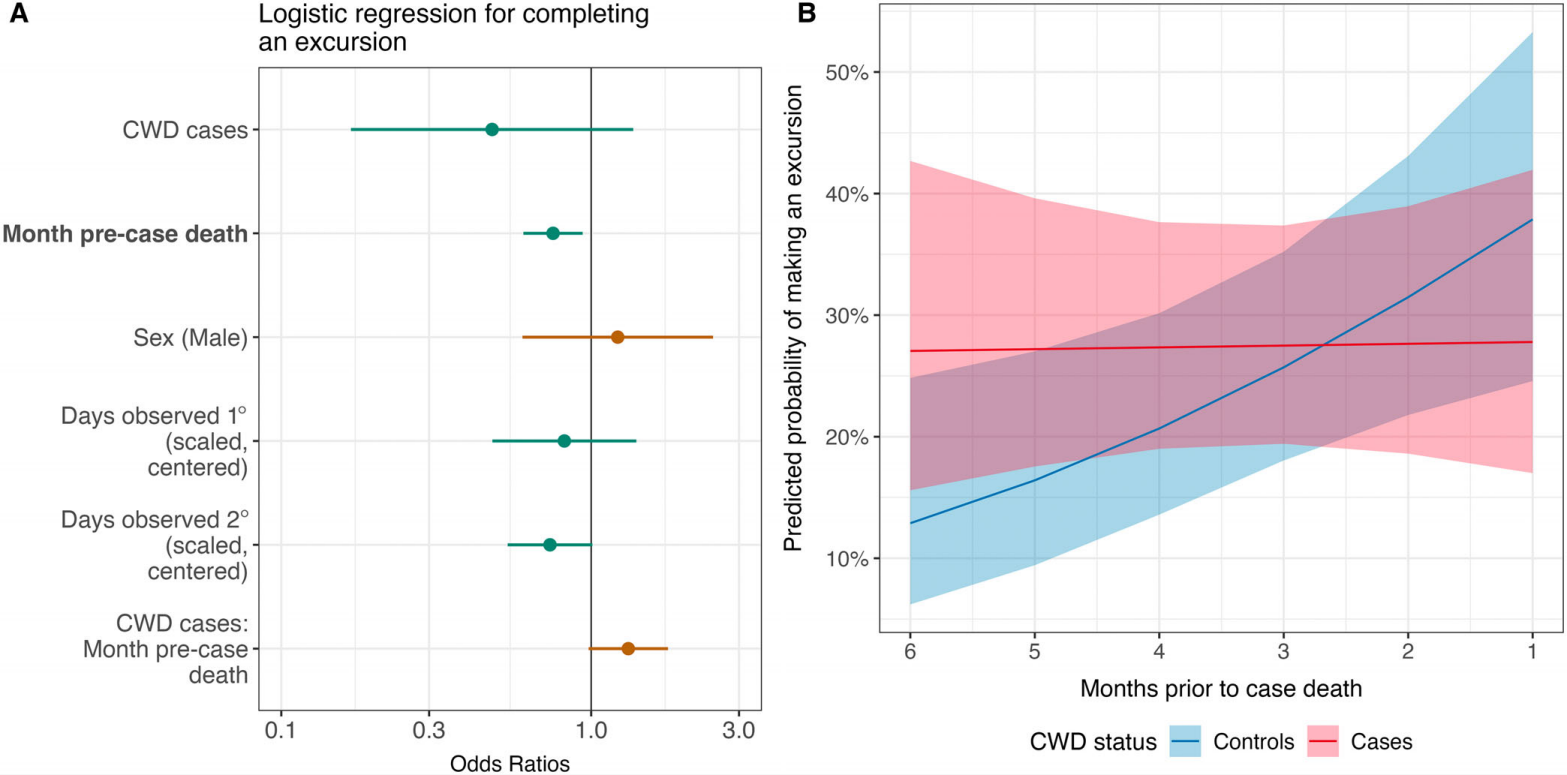
Generalized linear mixed model results for the monthly occurrence of an extra‐home range excursion among white‐tailed deer (*Odocoileus virginianus*) in southwest Wisconsin, USA. Panel (A) shows model results, with points representing estimates for each predictor and horizontal lines showing the 95% CIs. Statistically significant predictors are highlighted in bold; teal color indicates a negative estimate, and brown a positive estimate. CWD, chronic wasting disease; “days observed” was modeled as a second order polynomial, so 1° and 2° represent the first and second degrees of this predictor, respectively. Panel (B) gives the marginal effects of CWD status and months prior to death on the monthly probability of making an excursion. Lines are estimates and ribbons the 95% CIs; controls are shown in blue and CWD cases in red.

The mean distance of deer excursions was 1.99 km (range: 0.36–12.92 km). Excursion distance was significantly reduced for cases (cases mean = 1.49 km, range = 0.39–7.96 km; controls mean = 2.50 km, range = 0.36–12.92 km), though we observed no effect for months prior to case death (Figure 5; Table 1; Appendix S2: Table S7). In addition, within‐individual heterogeneity accounted for more variability in excursion distance than between‐individual heterogeneity (Table 1). Excursions generally lasted <1–2 days, but ranged up to about 11 days (Figure 5). Excursion durations did not significantly differ between cases and controls (Figure 5; Appendix S2: Figure S6, Table S8).

**FIGURE 5:**
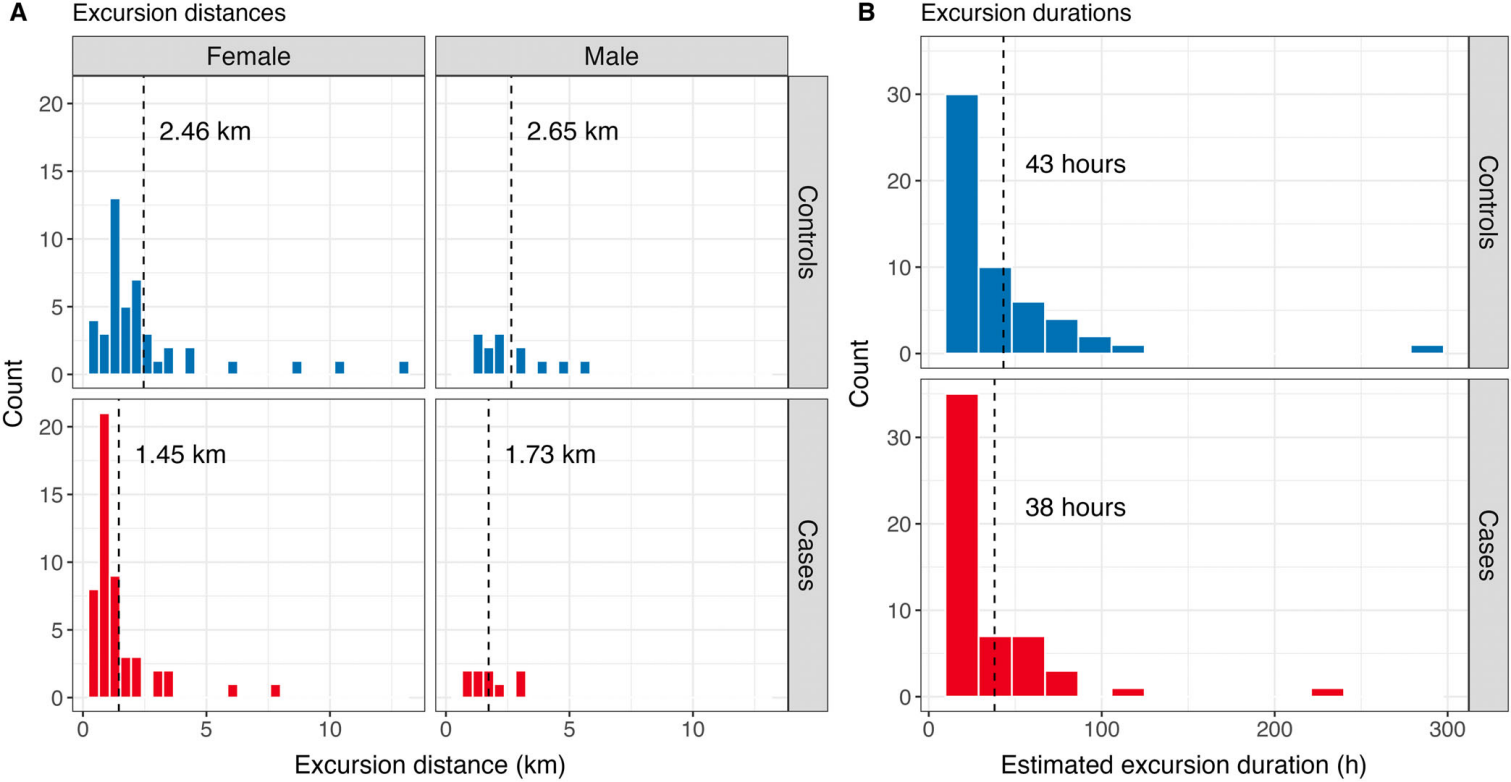
Distances and durations of extra‐home range excursions among white‐tailed deer (*Odocoileus virginianus*) in southwest Wisconsin, USA. Panel (A) shows histograms of excursion distances by sex and chronic wasting disease (CWD) status (case or control). Panel (B) shows histograms of excursion duration by CWD status. Because durations were evaluated as intervals, for each excursion, the plotted value is the mean of the shortest and longest possible duration (e.g., if an excursion was at least 20 h in duration, but <30 h, the plot would show a duration of 25 h). In both figures, vertical dashed lines and accompanying text give mean values.

**TABLE 1:** Linear mixed‐effects model results for log‐transformed excursion distance among white‐tailed deer (*Odocoileus virginianus*) in southwest Wisconsin.

Predictor^a^	Estimate	95% CI
Intercept	0.46	0.18 to 0.78
**CWD status: case**	**−0.45**	**−0.70 to −0.17**
Months prior to case death	0.04	−0.04 to 0.11
Sex: male	0.33	−0.06 to 0.65

*Note*: Statistically significant predictors are highlighted in bold text.

Abbreviation: CWD, chronic wasting disease.

^a^ Individual random effects variance = 0.01, SD = 0.11; residual random effects variance = 0.42, SD = 0.65.

### CWD and deer movement characteristics

Movement characteristics differed between cases and controls and as the time series progressed. Reduced movement rates, relative to controls, were associated with CWD cases, though this relationship was only statistically significant in the final month prior to case death (Figure 6). Sensitivity analyses demonstrated estimates were robust to GPS collar fix rate, though power to detect differences between cases and controls was reduced with less frequent fixes (Appendix S3). Reductions in case movement as death approached did not substantially differ based on case genotype at *PRNP* codon 96 (Appendix S2: Figure S7). Bayesian change point analysis indicated movement rates of cases decreased with time, while those of controls increased (Figure 7). Detected movement rate change points were at the very start of the time series for both cases and controls (cases: 177.6 days prior to case death, 95% highest‐density interval [HDI]: 173.7–180 [maximum] days; controls: 174.9 days prior to case death, 95% HDI: 166–180 [maximum] days); these estimates should be interpreted with caution as they lack a substantial steady state before the detected change points.

**FIGURE 6:**
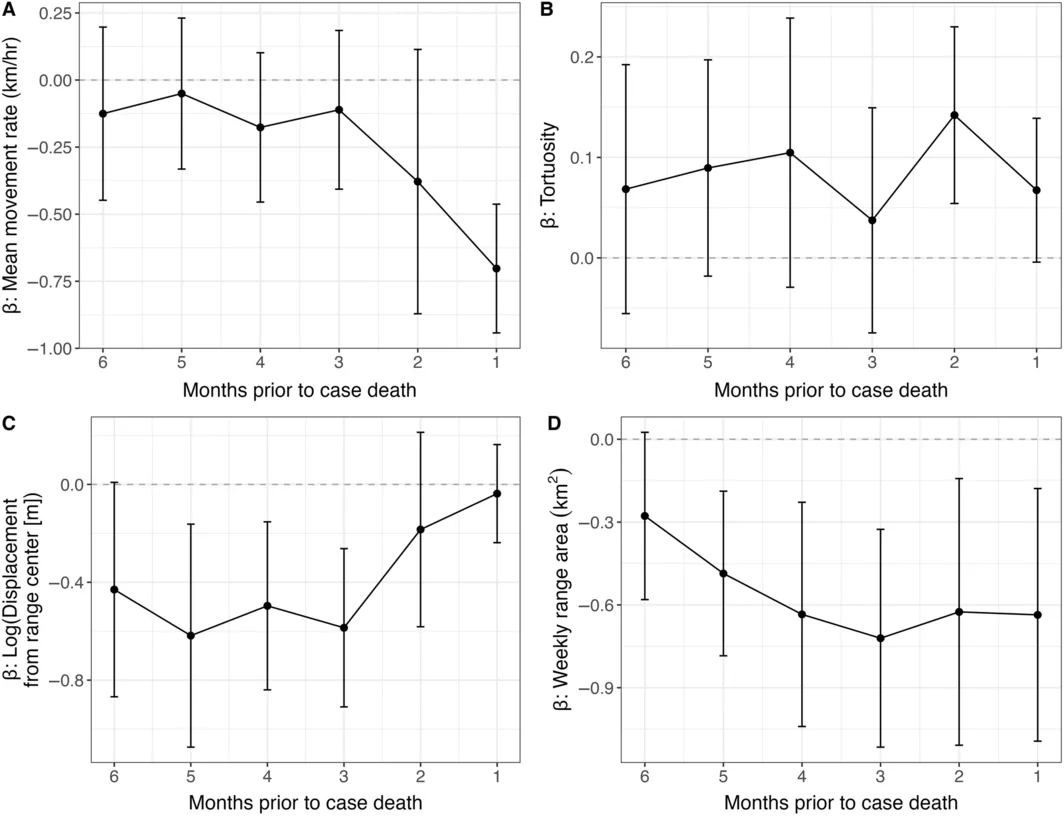
The relationship between key movement characteristics and chronic wasting disease (CWD) status for white‐tailed deer (*Odocoileus virginianus*) in southwest Wisconsin, USA, as quantified by conditional logistic regression. The response variable was CWD case or control status, so the *y*‐axis gives parameter estimates for the effect of each movement characteristic (β) on CWD status. A separate model was fit for each month prior to case death, so points represent the estimate from a given model, and bars are the 95% CIs. An estimate of zero indicates no relationship between a metric and CWD status; a negative value indicates case status was associated with lower metric values than controls.

**FIGURE 7:**
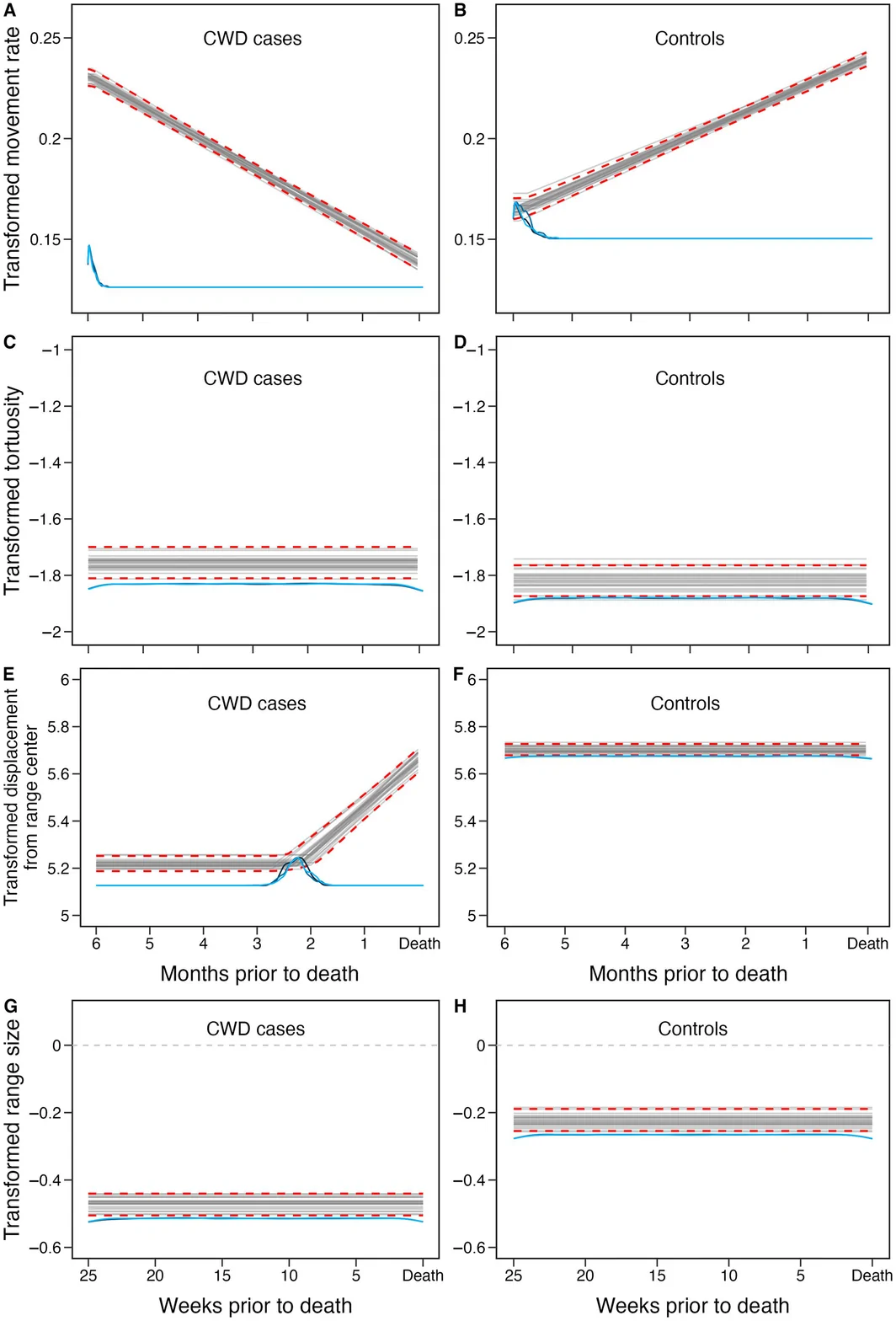
Bayesian change points for key movement characteristics among white‐tailed deer (*Odocoileus virginianus*) in southwest Wisconsin, USA. Panel columns represent chronic wasting disease (CWD) cases and controls. Blue lines in each plot show posterior model draws for the change point of a given metric. Solid gray lines give posterior draws for the fitted metric values, and red dashed lines the 95% quantiles. Movement characteristics were transformed to meet assumptions of normality: movement rate was square‐root transformed; tortuosity was scaled and centered, with an added constant and further log‐transformation; displacement from range center was log‐transformed; and range size was log_10_‐transformed (Appendix S1: Table S4). Note that *y*‐axes differ between, but not within, panel rows.

Increased tortuosity was associated with cases, but this relationship was only statistically significant in the second month prior to case death (Figure 6). The observed increase may be due to fix rate, as sensitivity analysis found tortuosity estimates were prone to positive bias as fix rates between matches became coarser. In addition, change point analysis failed to detect a significant behavioral change point for either cases or controls (Figure 7). CWD case status was associated with decreased displacement from a deer's monthly range center, relative to controls, for much of the 6 months prior to case death (Figure 6). However, this relationship disappeared in the final 2 months of the time series. Change point analysis demonstrated this shift arose from cases, rather than controls: cases increased their displacement starting about 2 months prior to death (70 days prior to case death, 95% HDI: 50–80 days; Figure 7). While sample sizes of CWD positive 96GS genotype deer were limited, we did not observe substantial qualitative differences in results when post hoc stratifying all daily conditional logistic regression models by genotype (Appendix S2: Figure S7).

Reduced weekly range size, relative to controls, was significantly associated with CWD cases, with the exception of the 6th month prior to case death (Figure 6). Change point analysis, however, failed to detect a clear shift in range size for either cases or controls, suggesting this difference arose prior to the 6‐month study window (Figure 5).

### CWD and deer habitat selection

Both CWD cases and controls generally preferred forested versus non‐forested habitat (cases mean selection estimate = 0.67, SD = 1.49; controls mean selection estimate = 0.44, SD = 1.20), and the strength of selection for forest was not associated with CWD status (Appendix S2: Figure S8). In addition, we did not detect a change point in relative selection for forested habitat for either cases or controls (Appendix S2: Figure S8). The relationship between selection for proximity to forest edges and CWD status was variable through time, with a significant preference for proximity to edges associated with cases 2 months prior to death (Figure 8). This timing was supported by change point analysis, which identified a shift in habitat selection about 2 months prior to case death (62.1 days prior to case death, 95% HDI: 52.9–71.0 days), with cases increasing selection for proximity to forest edges (i.e., decreased selection for distance to edges) as death approached. In contrast, control selection for forest edges remained unchanged.

**FIGURE 8:**
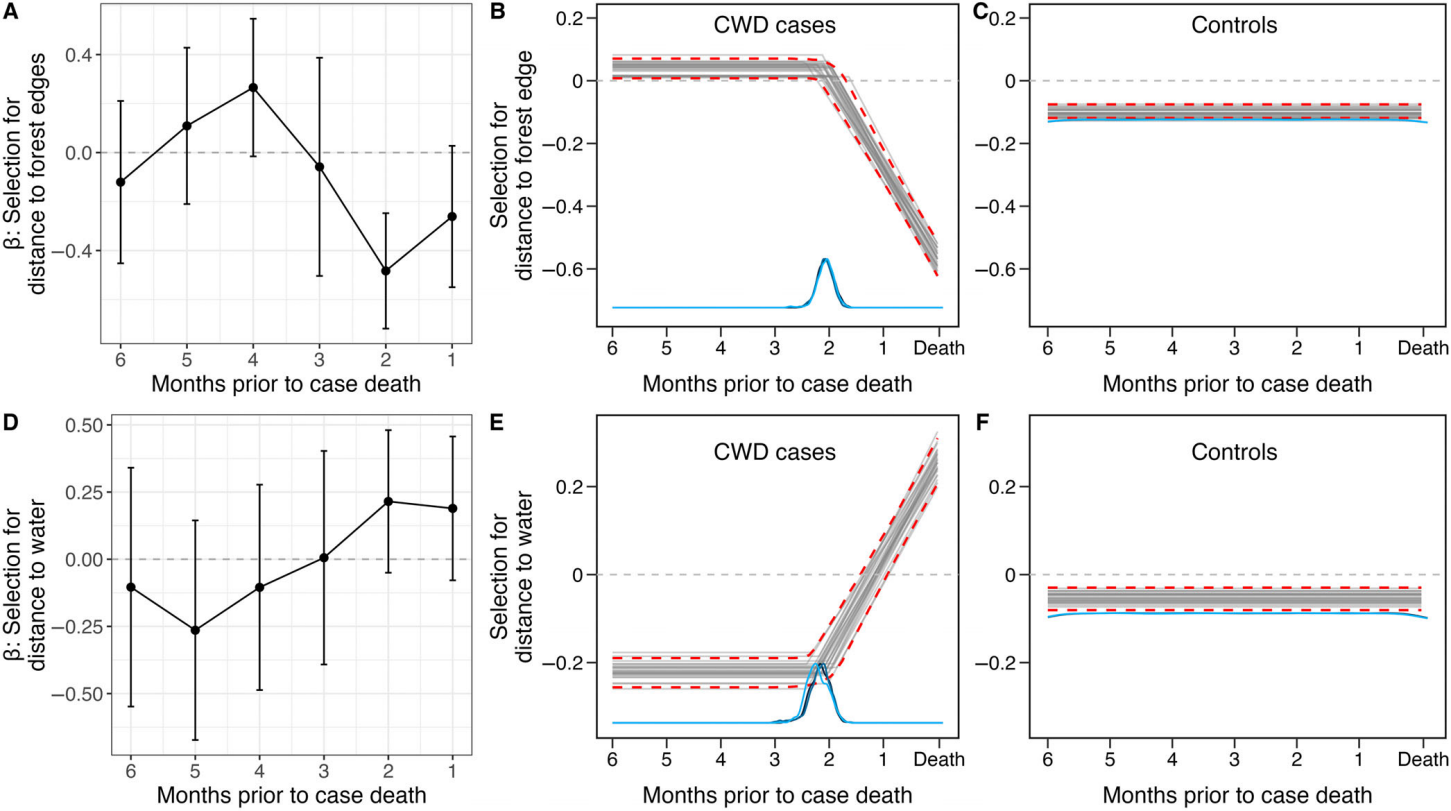
The relationship between key habitat characteristics and chronic wasting disease (CWD) status for white‐tailed deer (*Odocoileus virginianus*) in southwest Wisconsin, USA. Plots (A–C) show results for selection for distance to forest edges (i.e., the inverse of proximity to forest edges), and (D–F) results for selection for distance to water. Plots (A) and (D) give conditional logistic regression results, so the *y*‐axis gives parameter estimates (points) and 95% CIs (vertical bars) for the effect of each habitat characteristic (β) on CWD status. A negative estimate indicates case status was associated with reduced relative selection for that habitat characteristic, as compared to controls. Plots (B, C) and (E, F) give Bayesian change points for CWD cases (B, E) and controls (C, F). Blue lines in each plot show posterior model draws for the change point of a given metric. Solid gray lines give posterior draws for the fitted metric values, and red dashed lines the 95% quantiles of posterior densities of metrics. In these plots, zero indicates “no selection or avoidance,” so negative fitted values indicate relative avoidance of a given habitat characteristic.

Cases and controls generally preferred more easterly and southerly aspects than westerly or northerly (Appendix S2: Figures S9 and S10). Eastness was not associated with CWD status, and northness was only significantly associated with CWD status for the fourth month prior to case death (Appendix S2: Figures S9 and S10). We did not detect clear change points in selection for either component of aspect for cases or controls.

Selection for distance to water was not significantly associated with CWD status (Figure 8). However, change point analysis did detect a shift in case selection about 2 months prior to death (65.6 days prior to case death, 95% HDI: 53.7–76.5 days). Initially, cases preferred greater proximity to water than controls (though this was not associated with a significant relationship with CWD status in conditional logistic regression), but then shifted to increasing selection for greater distance to water sources.

In our analysis of deer mortality sites beyond case–control matches, the mean distance of mortalities to water sources was 0.55 km for CWD positive deer (*n* = 110, range = 0–2.47 km) and 0.57 km for test negatives (*n* = 141, range: 0–1.95 km). A subset of 163 mortalities (83 CWD test negative, 80 CWD positive) had recorded body condition scores at death. Among these individuals, CWD‐positive mortalities in good body condition were closest to water sources (*n* = 5; mean = 0.33 km, range = 0.03–0.69 km). However, we failed to detect a significant relationship between square‐root transformed proximity to water at death and CWD status or body condition (Table 2).

**TABLE 2:** Linear regression results for the square‐root transformed distance between white‐tailed deer (*Odocoileus virginianus*) mortality sites and the nearest water source in southwest Wisconsin.

Predictor	Estimate	95% CI
Intercept	21.9	(17.8–25.9)
CWD status: case	−5.7	(−16.3 to 4.9)
Fair condition	2.0	(−5.2 to 9.1)
Poor condition	−1.6	(−6.9 to 3.8)
Case: fair condition	1.2	(−13.9 to 16.2)
Case: poor condition	7.1	(−4.4 to 18.6)

*Note*: Reference category for CWD status was test‐negative individuals. “Condition” refers to body condition at death, for which the reference category was “good.”

Abbreviation: CWD, chronic wasting disease.

## DISCUSSION

### Phases of CWD infection

We sought to determine if and when a chronic infection, CWD, affects movement and habitat selection in its WTD host. We observed distinct changes in case movement behavior, but the timing of changes was variable across different movement and habitat selection metrics. This variability implies disease‐induced behavior changes do not occur in a single transition from a non‐clinical incubation phase to a clinical disease state. Generally, the changes we observed occurred at or before 6 months (movement rate, weekly range size) and 2 months (selection for forest edges, displacement from range center) before case death. Body condition scores were often high for cases at capture, even within 6 months prior to death, despite all cases being in poor condition when they died. We found no effect for the time between capture and death on body condition score, implying body condition changes occur relatively quickly and late in the disease process. The earliest movement changes (e.g., reduced weekly range sizes) therefore appear to precede the onset of overt, clinical symptoms associated with CWD (e.g., wasting). Typically, CWD is described as having a “preclinical phase” (or incubation period) that starts at the time of infection and includes the onset of the infectious period (start of prion shedding; Ketz et al., 2019). The preclinical phase is followed by the “clinical phase,” which begins just weeks to months prior to death (Williams, 2005; Williams et al., 2002; Williams & Miller, 2002). However, the reductions in movement rates and weekly range sizes we observed suggest the existence of an additional “subclinical phase” that falls between the preclinical and clinical phases (Figure 9). An animal in a subclinical phase of infection may not present as clinically ill on inspection (e.g., good body condition), but would exhibit changes in movement detectable by statistical analysis of behavioral data (e.g., reduced movement rates; Blaha et al., 2026). The late onset of clinically apparent, CWD‐induced changes is critical to convey to hunters, wildlife viewers, and the general public. If, for example, hunters only consider the possibility of CWD infection in a harvested deer if the animal visually appears “sick,” they may be less likely to submit samples for voluntary CWD testing, or may take fewer precautions to limit contact with or transportation of potentially infectious material (Yoder et al., 2025).

**FIGURE 9:**
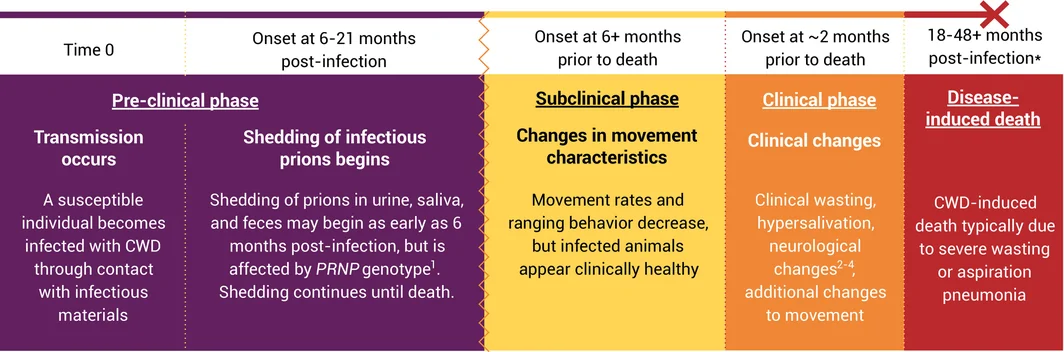
Proposed phases of chronic wasting disease (CWD) infection in white‐tailed deer (*Odocoileus virginianus*), as observed in Wisconsin, USA. The incubation period is not specifically shown, but would begin at the point of transmission and end at the onset of the clinical phase. The vertical zigzag represents a larger jump in time as the preclinical phase can be prolonged. * = The precise time between infection and death in free‐ranging deer is highly uncertain, and appears to vary based on host genotype at prion protein gene (PRNP) codon 96. 1 = Denkers et al. (2024); 2–4  = Williams et al. (2002), Williams & Miller (2002), and Williams (2005).

The timing of movement changes we observed was earlier than in studies of WTD and mule deer in Wyoming. In mule deer, movement rates decreased among CWD positive animals about 4 months prior to death (Barrile et al., 2024). Similarly, Edmunds et al. (2018) observed a “clinical phase” in WTD based on biaxial activity data, starting about 16 weeks before death. However, this latter finding was based on four individuals, and accelerometer data rather than GPS relocations, as used here. WTD in southwest Wisconsin likely have better access to high‐quality foraging resources than deer in Wyoming. Indeed, deer in arid regions like Wyoming generally have larger home ranges than deer in our study area (Stewart et al., 2011). The earlier reductions in WTD movement and ranging in our study could, therefore, result from a diminished need to range widely among the deer studied here; resources are plentiful, so subclinically affected deer could reduce their movements without sacrificing access to necessary resources. Nevertheless, the onsets of subclinical and clinical phases are not likely abrupt events. Rather, disease continuously progresses, with cumulative changes among a suite of symptoms eventually becoming apparent through direct observation or through analysis of movement data.

### Disease‐associated changes in movement and habitat selection

CWD case status was consistently associated with reduced movement and ranging behavior (e.g., movement rates, weekly range sizes). While daily displacement was reduced for cases in the initial months of the time series, we did observe a late increase in the final 2 months of case life, while weekly ranges remained small. This seemingly counterintuitive result may have arisen from the different time scales of measurement for these two metrics. However, the timing of the increased displacement among cases aligned with the change points we observed for habitat selection (forest edges and water sources); the late increase in displacement could therefore represent shifting space use within a home range. While we typically did not observe behavioral changes among our controls, we detected increases in movement rates and excursions for controls within the 6 months prior to case death. Most cases died in January to April, so a preponderance of our movement data—especially for the final months of case life—came from these months (Appendix S2: Figure S11). Matching cases and controls by day ensured differences in movement behavior between the two CWD classes did not arise from simple seasonal differences within pairs. However, uneven data coverage throughout the calendar year implies the movement characteristics we observed are more representative of deer behavior during winter to early spring than other times of the year. This period is associated with major shifts in movement behavior among healthy deer, with deer increasing their activity and movement rates (Gilbertson et al., 2025). The observed increase in control movement therefore likely reflects a seasonal shift in behavior, and highlights the relative difference with cases, which decreased their movement rates.

The reductions we observed in deer movement and ranging among cases suggest the spatial footprint of CWD transmission risk shrinks as an individual's disease progresses. For example, reduced weekly range sizes and reduced displacement among cases would generate a smaller area of higher concentration prion contamination, with this change starting at or before 6 months prior to death. However, a reduced footprint of environmental contamination does not necessarily equate to reduced transmission risk (Dougherty et al., 2018; Farnsworth et al., 2006; Manlove et al., 2022). Within‐group individuals may experience higher infection risk due to the increased contamination of their local range area; this differs from highly mobile hosts, who are able to escape contaminated ranges (Altizer et al., 2011; Pandey et al., 2024). However, this effect assumes CWD‐induced illness does not significantly alter social group cohesion and spatial overlap (e.g., through avoidance of sick conspecifics; Curtis et al., 2004; Lopes et al., 2022; Sarabian et al., 2023). Regardless, the prolonged infectious period of CWD implies within‐group individuals experience high transmission risk, even before any disease‐induced behavior changes can occur. Indeed, transmission appears to occur readily within social groups (Grear et al., 2010; Schauber et al., 2007).

The potential effects of reduced movement and ranging on CWD transmission may be more important between groups. In general, reduced ranging implies fewer opportunities for between‐group contact—direct or environmental—as reductions in space use or mobility may limit spatial and spatiotemporal overlap with conspecifics (Dougherty et al., 2018; Pandey et al., 2024). However, although case excursions were shorter distances, their duration and probability of occurrence were comparable to those of controls. For context, the average seasonal range size for deer in this region is 1.07 km^2^ (SD = 0.81; Gilbertson et al., 2025), so a mean excursion distance of 1.49 km among cases represents a substantial departure from the normal range. Dispersals in this region are generally longer distances than excursions (median dispersal distance of 5.77 km in Gilbertson, Ketz, et al., 2022), but typically occur among juvenile males and only once or twice in a lifetime, while excursions occur among both sexes and appear to be a far more regular occurrence. The risk of CWD spread through extra‐home range transmission events may therefore be reduced, but not eliminated, as disease progresses within an individual. Further, both between‐ and within‐group transmission rates depend on direct and environmental contact rates, and we did not specifically test for changes in contact rates associated with CWD. Contact rates and transmission risk could be reduced, for example, if sick deer are less socially active (as with immune challenge in house mice *Mus musculus domesticus*, Lopes et al., 2016; similar to aging‐related changes in red deer *Cervus elaphus*, Albery et al., 2022) or if healthy conspecifics avoid clinically ill individuals (Curtis et al., 2004; Lopes et al., 2022; Sarabian et al., 2023).

Contact rates may be affected not just by changes in movement characteristics, but also by shifts in the habitats in which individuals overlap directly or indirectly. Habitat selection was largely unchanged by CWD infection, aside from selection for proximity to forest edges. An increased preference for proximity to these edges among our cases could support the generation of transmission “hot spots,” as forest edges are generally preferred by WTD, regardless of CWD status (Gilbertson et al., 2025). This is similar to conclusions by Edmunds et al. (2018) who argued riparian areas could be CWD transmission hotspots, as they were selected for by both CWD cases and test‐negative individuals. In these instances, increased selection by cases could concentrate CWD contamination in high‐value habitats—conditions favorable for “ecological traps” (Hale & Swearer, 2016; Robertson & Hutto, 2006). These traps could have significant consequences for larger population health, as the potential fitness benefits of high‐value forest edge habitats (Beier & McCullough, 1990; Miyashita et al., 2008; Nixon et al., 1991; Rohm et al., 2007; VerCauteren & Hygnstrom, 2011) could be negated by an increased probability of CWD transmission. Future work could examine direct and indirect contact rates—especially among between‐group individuals—by infection status and habitat to determine if the changes in habitat selection we observed are adequate to produce ecological trap‐like effects.

The effect of CWD‐associated changes to deer movement or habitat selection on subsequent transmission depends on when these changes occur relative to the onset of transmissibility. Because CWD shedding begins in the preclinical phase of infection, and the duration of the incubation period is long (18–48 months; Williams, 2005; Williams et al., 2002; Williams & Miller, 2002) relative to the clinical and proposed subclinical phases of infection, typical “healthy” deer movement and habitat selection likely represent transmission‐relevant behaviors for much of the infectious period. As such, movement data from typical deer is likely adequate for informing habitat selection and the spatial extent of deer movement in models of CWD transmission dynamics. However, this conclusion is likely affected by deer *PRNP* genotype. The vast majority of our study cases were genotype 96GG; Denkers et al. (2024) showed WTD of this genotype can shed CWD prions at or even before testing RAMALT positive. Our sample size of 96GS cases was too limited for robust inference, but our post hoc assessment of movement metrics suggested movement changes associated with CWD did not substantially differ between 96GG and 96GS deer. Given that high prevalence of CWD can select for increased frequency of 96GS genotypes (Ketz et al., 2022), future research could more closely examine interactions between the timing and duration of prion shedding for each genotype, relative to CWD‐induced behavioral changes.

From a management perspective, the smaller range sizes, reduced movement rates, and increased preference for forest edges among CWD cases could affect these individuals' susceptibility to hunter harvest or predation in the final 6 months of life. The effects of CWD on harvest susceptibility are still uncertain: Edmunds et al. (2016) found CWD cases were overrepresented among hunter harvested deer, while Grear et al. (2006) found no relationship between harvest susceptibility and CWD. Increases in harvest or predation susceptibility, if they do occur, may arise from reduced vigilance and avoidance of hunters or predators during progressive CWD infection. Alternatively, reduced activity among cases may decrease detection by hunters (similar to decreased harvest success when hunting seasons occur after peak rut, when deer activity is decreasing; Hunsaker et al., 2025). Changes to diel activity cycles in response to CWD (Edmunds et al., 2018) could similarly alter susceptibility, though we lacked the high temporal resolution movement data necessary to test for these shifts. Assuming CWD alters movement prior to changes in mentation that could cause decreased vigilance, we speculate that reduced movement among subclinically affected deer may make them less susceptible to hunter harvest than CWD‐negative individuals. As CWD‐infected deer progress toward clinical infection, however, changes in vigilance and habitat selection could then make deer more vulnerable to harvest than their healthy counterparts. Whether these patterns would hold true for predation vulnerability is unclear and merits additional research, as predation may play an important role in CWD dynamics (Brandell et al., 2022). Nonlinear changes to harvest susceptibility as CWD progresses would make such a relationship more difficult to detect with simple correlative studies based on CWD status at the time of harvest. Future work could consider metrics for the stage of CWD at death as an additional covariate to help determine if harvest susceptibility and CWD status are non‐monotonically related.

### CWD and attraction to water

Despite polydipsia being a known symptom of CWD, selection for proximity to the nearest water source was not a significant predictor of CWD status, nor did CWD‐positive individuals die in closer proximity to water sources than test‐negative deer. However, we did observe a change point in selection for proximity to water for cases, which was characterized by an initial preference for proximity, followed by a shift to selection for increased distance about 2 months before death. Curiously, Barrile et al. (2024) found a similar change and timing in mule deer. In addition, while not statistically significant, we note that CWD‐infected deer in good body condition (i.e., not in the clinical phase) died in closer proximity to water sources than test‐negative or other test‐positive deer. As Barrile et al. (2024) suggested, this apparent shift to greater distances from water sources could be spurious, as we lack a clear mechanism to explain this observation. Alternatively, other changes during the clinical phase of CWD infection could be correlated with or supersede polydipsia (e.g., preference for forest edges could outweigh preference for water sources). Attraction to water sources may also occur at a finer spatiotemporal scale than we could evaluate here. The average time between fixes was relatively high (9 h), compared to the probable time scale of selection for water resources. Further, some water sources may be too small and ephemeral to be included in remote sensed data. These spatiotemporal scale limitations may be especially relevant for our study area, where water is not a limited resource, as compared to mule deer in the mountain west. Furthermore, while CWD case deaths were distributed throughout the calendar year, a preponderance occurred in winter to early spring. As such, the preceding calendar months may be overrepresented in our data. In southwest Wisconsin, many water sources freeze during the winter, which could weaken any relationships between CWD status and attraction to water. Given these uncertainties, we caution against over‐interpreting these results. Future research may examine the relationships between attraction to water and CWD status more specifically, especially at finer spatiotemporal scales. Water resources or riparian areas—especially in water limited regions—could be attractive sites that favor transmission, so these relationships merit additional investigation.

### Limitations and future directions

Our relatively coarse fix rates restricted our ability to evaluate changes in short duration behaviors (e.g., contact patterns, drinking events). They also may result in differences in habitat selection estimates as compared to studies with other fix rates (Thurfjell et al., 2014), or in underestimates of excursion frequency, duration, and distance; our excursion results can therefore be interpreted as conservative estimates. In addition, fix rates and durations of observation were initially variable within matches. Our sensitivity analyses guided our decision to downsample fix rates to match within pairs and ensured our results were conservative. Similarly, sensitivity analyses determined our conclusions were robust to varying sample sizes (i.e., different numbers of matched pairs) through the time series that arose from different durations of observation. Challenges with fix rates and observation durations are common in animal telemetry studies (Hofman et al., 2019; Krause et al., 2013). In particular, movement data with high fix rates are difficult to achieve when studying diseases with long incubation periods like CWD: monitoring may have to be prolonged to capture the onset of clinical signs, but most GPS collars cannot maintain high fix rates over these long periods due to limited battery life (Krause et al., 2013). Continued improvement of GPS collar technology could provide valuable opportunities for improving our understanding of the relationships between wildlife behavior and chronic diseases or those with long incubation periods.

Case–control studies are relatively rare for wildlife health studies (Rabinowitz et al., 1999) and can provide valuable insight into the effects of disease on animal behavior. Nevertheless, our study was limited as a retrospective study with only one control for each case and a 6‐month observation window (Grimes & Schulz, 2005). To mitigate the impacts of our limited number of controls, we attempted to closely match all case–control pairs, including to the level of the day of the year. Future studies with larger sample sizes may be able to detect significant differences between cases and controls of smaller magnitude than we could here. Our 6‐month observation window matched that in Barrile et al. (2024), and was further justified by observations in other studies that most changes in behavior with CWD occurred within the final few weeks to months of life (Williams, 2005; Williams et al., 2002; Williams & Miller, 2002). In addition, extending our 6‐month window further had diminishing returns, as day matching between cases and controls decreased with increased duration of the observation period. Nevertheless, because some movement metrics (e.g., range size) changed prior to 6 months before CWD‐induced death, future work may consider using longer observation windows to identify these change points with more precision.

We defined cases as deer that were CWD positive at death and in poor body condition to focus on deer in “end stage” CWD (i.e., had severe clinical signs). One female case was less than a year of age, which would typically not be associated with end stage CWD. This individual's immediate cause of death was a vehicle collision, but she was CWD positive with no obvious body fat at the time of death, thereby meeting our case definition. If this or other cases' cause of poor body condition was not CWD (i.e., CWD was an incidental finding), we would expect their movement and habitat selection prior to death to be more similar to controls than true end stage cases. Our case definition was therefore conservative, and the changes we observed in case behavior may be underestimates.

## CONCLUSIONS

CWD‐infected deer reduced their movement, weekly range sizes, and excursion distances in the final months of life. Habitat selection was largely unchanged, but CWD cases did show increasing preference for proximity to forest edges. Behavior changes appeared to occur primarily at two time points prior to CWD‐induced death (at or before 6 months or 2 months before death), with changes in body condition occurring very late in the disease course. These different change points imply CWD progression may fall into three phases—preclinical, subclinical, and clinical—though transitions through these phases are likely gradual. To maximize voluntary CWD testing, public communication—especially with hunters—could be more effective by emphasizing that CWD‐infected deer only appear visibly sick very late in the disease course. While reductions in movement and ranging behavior may limit subsequent transmission, further research on direct and indirect contact rates among CWD cases would be needed to understand the full implications of these results. Further, because changes in movement and habitat selection occur late in CWD's prolonged infectious period, typical deer movement is likely adequate to inform models and predictions of disease spread.

## CONFLICT OF INTEREST STATEMENT

The authors declare no conflicts of interest.

## Supporting information


Appendix S1.

Appendix S2.

Appendix S3.

## Data Availability

Code (Gilbertson, 2026) is available in Zenodo at https://doi.org/10.5281/zenodo.21983271. Deer genotyping data (Herbst et al., 2025) are available in a U.S. Geological Survey data release via ScienceBase at https://doi.org/10.5066/P137CERE. Additional data (capture, mortality, necropsy, and movement data from the Southwest Wisconsin CWD, Deer and Predator Study through February 2024) were obtained via a data sharing agreement with the Wisconsin Department of Natural Resources and are not publicly available; data requests can be directed via email to Daniel Storm (danielj.storm@wisconsin.gov) or the Office of Applied Science Wildlife Research Team Leader (David MacFarland; david.macfarland@wisconsin.gov).
