# Supplementary material for: Malady in motion: Effects of chronic wasting disease on host movement
Source: Ecol Appl. 2026 Oct 23;36(7):e70338. doi: 10.1002/eap.70338 (PMC13694198; doi:10.1002/eap.70338)
Supplement: Supplementary file 1 [file EAP-36-e70338-s001.pdf]

APPENDIX S3: SENSITIVITY ANALYSES

Journal: ***Ecological Applications***

Malady in motion: Effects of chronic wasting disease on host movement
Marie L. J. Gilbertson, Daniel P. Walsh, Daniel J. Storm, Allen Herbst, Wendy C. Turner

Any use of trade, product, or firm names is for descriptive purposes only and does not imply endorsement by the U.S. Government.

Table of Contents

Section S1: Fix rate sensitivity analyses overview.......... 2
Section S2: Within-pair fix rate sensitivity.......... 2
Section S3: Between-pair fix rate sensitivity.......... 5
Section S4: Time-varying resource selection function sensitivity analyses.......... 8
Section S5: Sensitivity of conditional logistic regression to sample size changes.......... 16
Section S6: Supporting references.......... 17

Section S1: Fix rate sensitivity analyses overview

We used global positioning system (GPS) collar data from white-tailed deer (*Odocoileus virginianus*) in southwestern Wisconsin, USA to determine when and how deer movement and habitat selection change with chronic wasting disease (CWD) infection. CWD cases were paired with age-, sex-, and day-matched controls. However, we did not match pairs based on the time between their GPS collar relocations (fix rate). Fix rates therefore frequently differed within and between pairs. To determine potential impacts of these fix rate differences, we performed two sensitivity analyses: (1) within-pair fix rate sensitivity, and (2) between-pair fix rate sensitivity.

Section S2: Within-pair fix rate sensitivity

Fix rates were usually mismatched within case-control pairs, but we identified seven pairs with >30 days of observations and matching within-pair fix rates. We treated these as "ideal" pairs (*ideal dataset*). We calculated daily mean movement rates and tortuosity values for each individual. These two metrics were then predictors in univariate conditional logistic regressions for CWD status for each pair, with "day" as the strata. We extracted the coefficient estimate for CWD status for each model.

We downsampled data from cases and controls to represent the various types of mismatched fix rates we could see within pairs in our full dataset. For example, if a pair initially had matching 2-hour fix rates, we would generate the following mismatched datasets:

Case fix rate (hours)	Control fix rate (hours)
2	4
2	12
2	24
4	2
12	2
24	2
4	12
4	24
12	4
24	4
12	24
24	12

We would then "correct" the fix rate for either the case or control to match the fix rate of the other individual (*corrected datasets*). Corrections were either via downsampling so both individuals matched at the coarser fix rate, or interpolating so both individuals matched at the higher frequency fix rate. For example, if the case fix rate was every 2 hours and the control fix rate was every 4 hours, a downsampling correction would result in both individuals having fixes every 4 hours; an interpolating correction would have both individuals with fixes every 2 hours. Interpolations were performed using the crawlWrap() function (default parameters) in the *momentuHMM* package (McClintock and Michelot 2018) in R v4.4.0 (R Core Team 2024).

For each corrected dataset, we repeated calculating daily mean movement rates and tortuosity values and fitting conditional logistic regressions, as done with the ideal dataset. We then plotted the coefficient estimates and 95% confidence intervals generated from the ideal dataset against those from the corrected datasets (Appendix S3: Figures S1-S2). Tortuosity was not calculated for fix rates >13 hours, as three relocations are necessary to calculate a single tortuosity value.

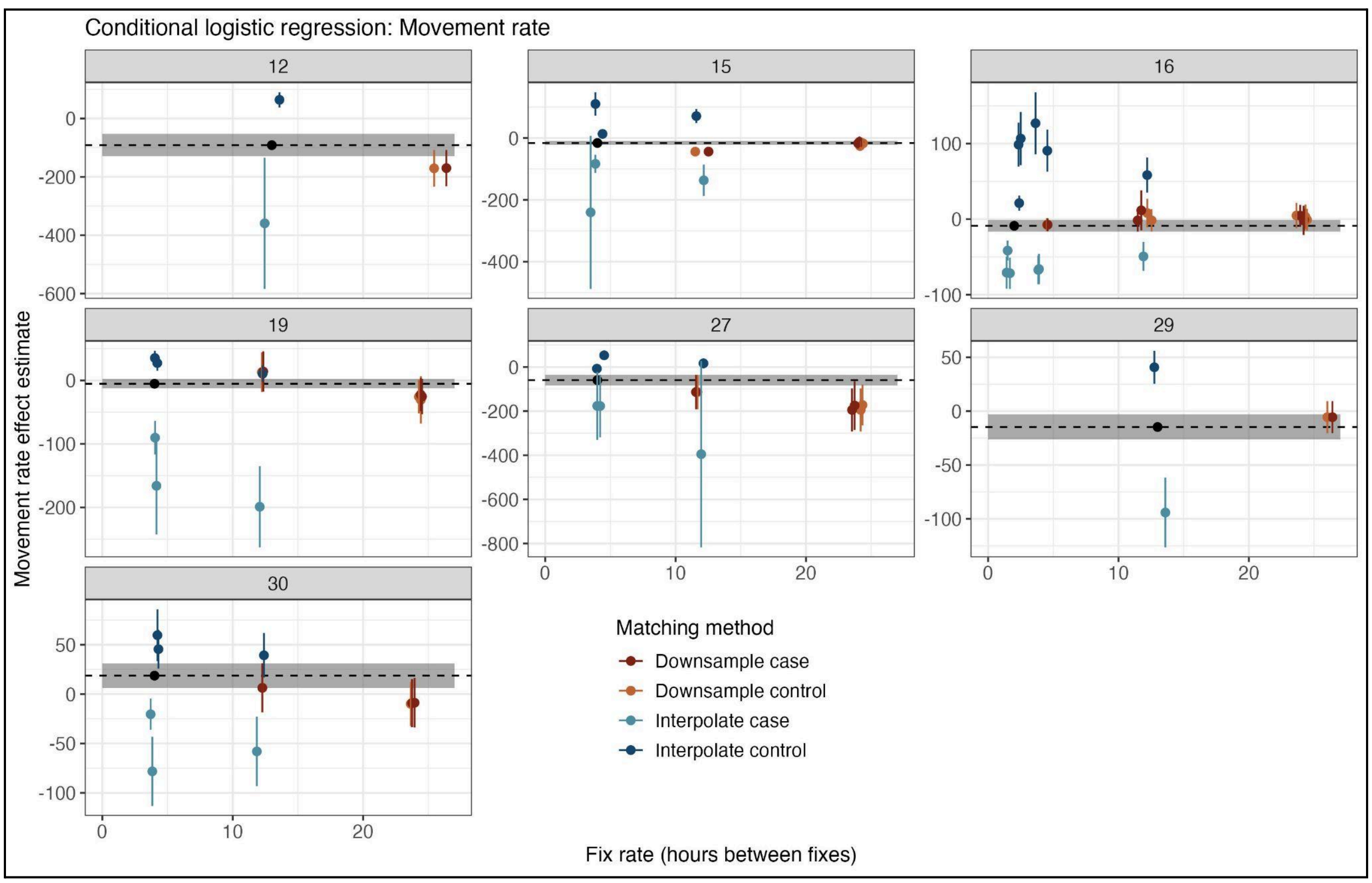

Figure S1: *The effect of correcting global positioning system (GPS) collar fix rates within matched Wisconsin, USA white-tailed deer (*Odocoileus virginianus*) pairs on subsequent inference for movement rates. Plots show conditional logistic regression (conditional LR) estimates for the predictive effect of movement rates for chronic wasting disease (CWD) status. Each panel gives results from a different matched pair (numbers indicate the pair number). The black dot and dashed line show the movement rate effect estimate from the "ideal" original*

dataset in which both individuals had matching fix rates. The position of the black dot along the x-axis shows the original matching fix rate with the pair. The gray ribbon gives the 95% confidence interval (CI) for the estimate from the ideal data. Fix rate mismatches were then generated by downsampling the case and/or control. Blue dots and vertical lines show estimates and 95% CIs when interpolation was used to correct simulated mismatches; red dots and vertical lines give these values when downsampling was used. The 95% CIs for these corrected datasets should ideally include the estimate from the ideal dataset. Points and accompanying CIs are jittered along the x-axis for visibility.

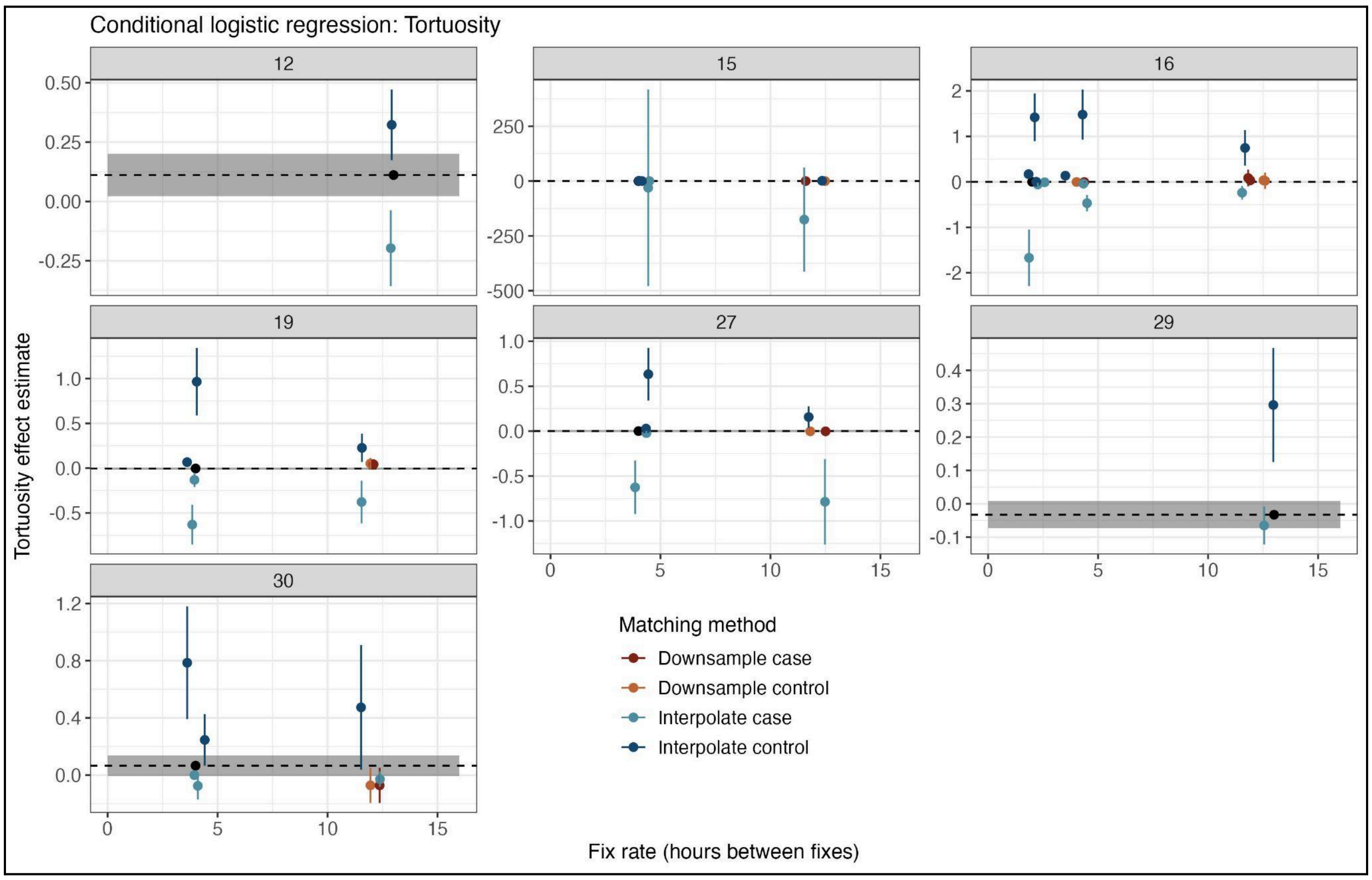

Figure S2: *The effect of correcting global positioning system (GPS) collar fix rates within matched Wisconsin, USA white-tailed deer (*Odocoileus virginianus*) pairs on subsequent inference for tortuosity. Plots show conditional logistic regression (conditional LR) estimates for the predictive effect of tortuosity for chronic wasting disease (CWD) status. Each panel gives results from a different matched pair (numbers indicate the pair number). The black dot and dashed line show the tortuosity effect estimate from the "ideal" original dataset in which both individuals had matching fix rates. The position of the black dot along the x-axis shows the original matching fix rate with the pair. The gray ribbon gives the 95% confidence interval (CI) for the estimate from the ideal data. Fix rate mismatches were then generated by downsampling the case and/or control. Blue dots and vertical lines show estimates and 95% CIs when interpolation was used to correct simulated mismatches; red dots and vertical lines give these*

values when downsampling was used. The 95% CIs for these corrected datasets should ideally include the estimate from the ideal dataset. Tortuosity was not calculated for fix rates >13 hours, as ≥3 relocations are necessary to calculate a single tortuosity value. Points and accompanying CIs are jittered along the x-axis for visibility.

Confidence intervals from models using downsampled corrected data more consistently overlapped with estimates—or at least 95% confidence intervals—from the ideal datasets. This was true for both movement rate and tortuosity. Interpolation frequently resulted in extremely wide confidence intervals and vastly incorrect estimates. Adjustments to the interpolation workflow (e.g., more tailored parameterization of the crawlWrap() function) may have been able to reduce the extremity of these errors, but were outside the scope of this study. We did not observe a clear difference in performance when downsampling case versus control movement data. Given the better performance of downsampled datasets, compared to interpolated, we elected to use downsampling to match fix rates within pairs for our analyses in the main text.

Section S3: Between-pair fix rate sensitivity

Within the subset of case-control pairs with matching fix rates, a further subset had matching fix rates of a recorded location at least every 4 hours (n = 5). To determine the effect of mismatched fix rates between pairs, we used this "high fix rate" subset of pairs as the "ideal" dataset. In the interest of most closely representing our analysis completed in the main text, we fit a conditional logistic regression with CWD status as the response variable, and both movement rate and tortuosity as predictors, with day as the strata and data clustered by matched pair. We extracted the coefficient estimates and 95% confidence intervals from this model fitted to "ideal" data.

To generate datasets with fix rates that vary between pairs (but not within), we completed 500 simulations. In each simulation, pairs were randomly assigned to fix rates of every 4, 8, or 12 hours and downsampled accordingly. Daily movement rates and tortuosity were calculated and a conditional logistic regression fit to the simulated dataset. Following all simulations, we compared estimates from simulations to those from the ideal dataset (Appendix S3: Figures S3-S4).

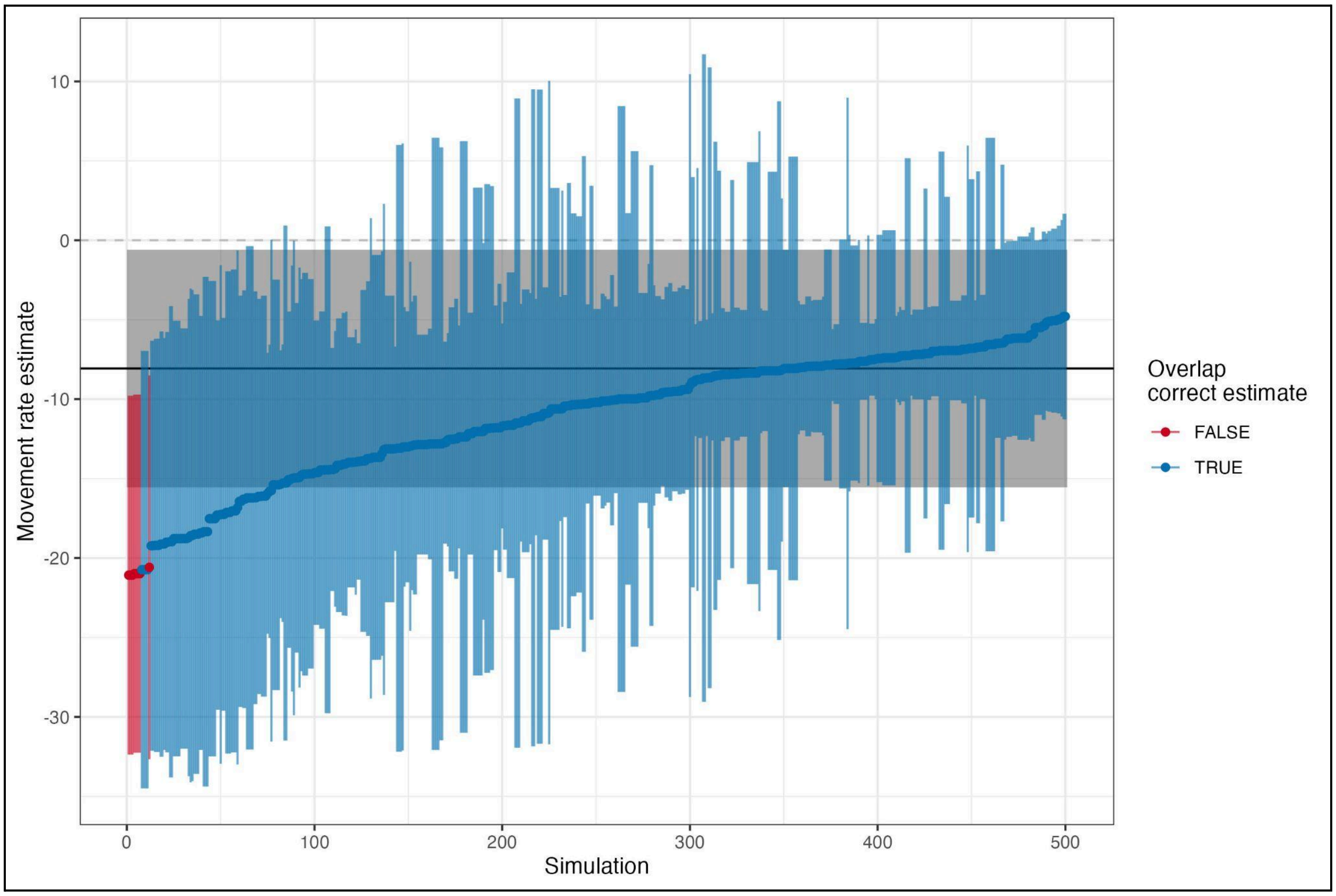

Figure S3: *The effect of variable between-individual fix rates on movement rate inference among Wisconsin, USA white-tailed deer (*Odocoileus virginianus*). Red and blue points represent effects estimates from conditional logistic regressions for chronic wasting disease status when data was downsampled to simulate variable fix rates between case-control pairs; the associated vertical lines are the 95% confidence intervals (CIs). The horizontal black line gives the effect estimate with the full, "ideal" dataset, and the gray ribbon the associated 95% CI. The horizontal dashed line at zero indicates "no effect." Points and vertical lines are blue if the 95% CI from downsample data includes the estimate from the ideal dataset. If estimates are robust to variable fix rates, 95% CIs from downsampled datasets should nearly always include the estimate from the full dataset (i.e., points and lines should be blue).*

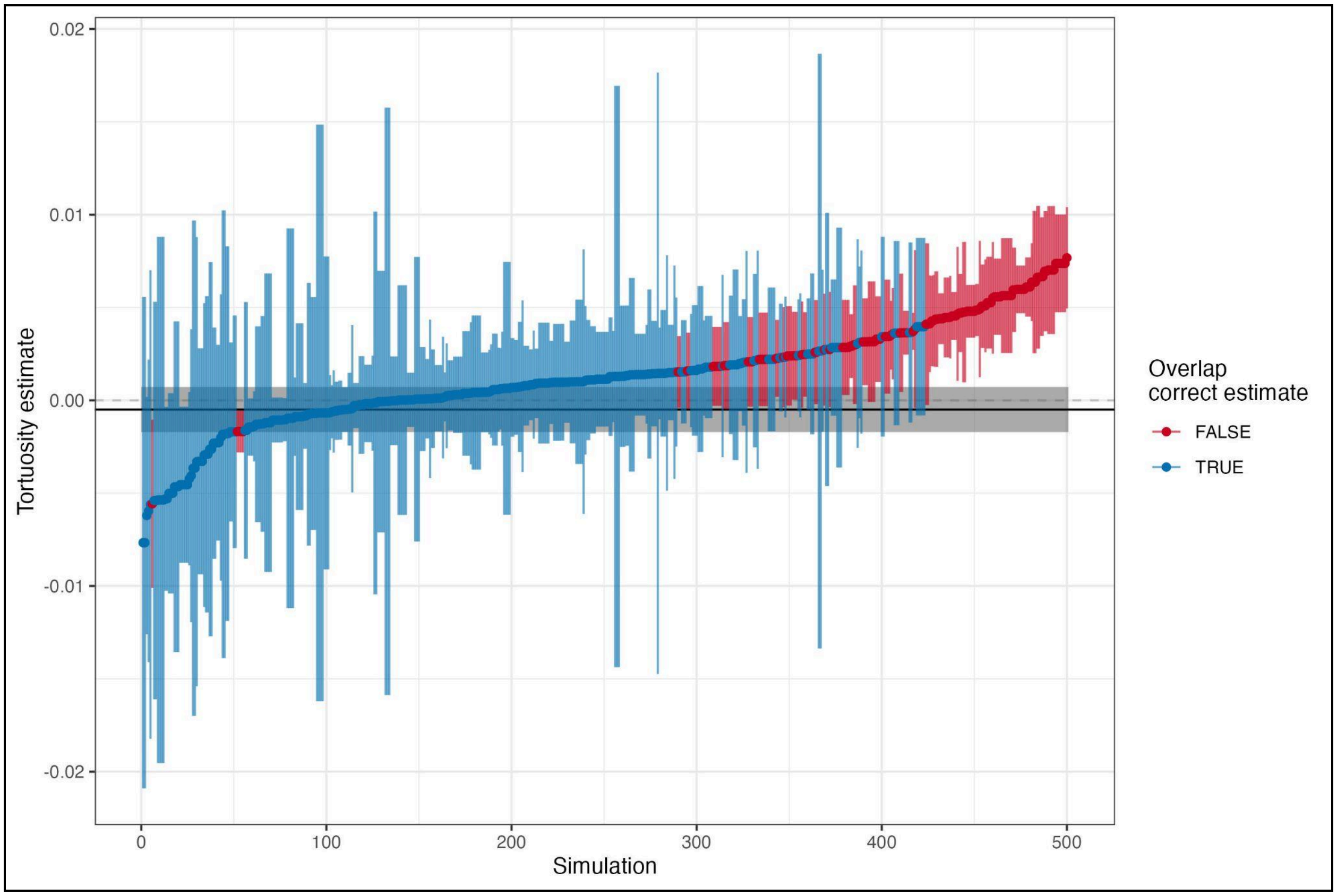

Figure S4: *The effect of variable between-individual fix rates on tortuosity inference among Wisconsin, USA white-tailed deer (*Odocoileus virginianus*). Red and blue points represent effects estimates from conditional logistic regressions for chronic wasting disease status when data was downsampled to simulate variable fix rates between case-control pairs; the associated vertical lines are the 95% confidence intervals (CIs). The horizontal black line gives the effect estimate with the full, "ideal" dataset, and the gray ribbon the associated 95% CI. The horizontal dashed line at zero indicates "no effect." Points and vertical lines are blue if the 95% CI from downsample data includes the estimate from the ideal dataset. If estimates are robust to variable fix rates, 95% CIs from downsampled datasets should nearly always include the estimate from the full dataset (i.e., points and lines should be blue).*

Movement rate effects estimates were robust to varying fix rates between pairs: 98.4% of models fitted to simulated datasets resulted in 95% CIs that contained the "true" value from the ideal dataset. Power, however, was reduced, as only 65.2% of estimates were correctly assigned statistical significance. Tortuosity was more sensitive to fix rates between pairs: 70.4% of models fitted to simulated data produced 95% CIs that contained the true value. Nearly all CIs that did not include the true value produced positive effects estimates (i.e., estimates were biased high). In addition, 76.6% of models fitted with simulated data correctly failed to detect statistical significance (i.e., tortuosity was associated with increased Type I error). We therefore would encourage caution when interpreting tortuosity results.

Section S4: Time-varying resource selection function sensitivity analyses

As described in the main text, we fit time-varying resource selection functions (tvRSFs; Dejeante et al. 2024) to the movement data for each deer in our study. Estimates from tvRSFs can be affected by the number of random locations drawn for each observed location and the initial value the user selects for the *Q* parameter (which affects the smoothness of the resulting time series estimates). In addition, tvRSFs do not account for temporal autocorrelation, so high frequency movement datasets may produce biased estimates. We performed sensitivity analyses to determine if (1) the number of random locations, (2) the initial *Q* parameter, or (3) temporal autocorrelation were likely to have an effect on our results.

We fit tvRSFs across a range of settings for each of these three concerns: the number of control points could be 10, 20, 50, or 100 per observed location; the initial *Q* parameter could be set to 0.1, 0.5, 2, or 4; and the dataset was either the full dataset (potentially autocorrelated) or a downsampled one (one location every 12 hours). We varied these settings in a full factorial design, for a total of 32 different variations across which to fit tvRSFs. Predictors were all tvRSFs were the same: distance to nearest water source, forested (versus non-forested) habitat, distance to nearest forest edge, eastness, and northness. Distances to water and forest edges were scaled and centered. After fitting tvRSFs, we calculated the mean daily selection coefficient for each predictor across individuals, and plotted the resulting time series to visually assess the impact of the tvRSF settings on our coefficient estimates (Appendix S3: Figures S5-S10).

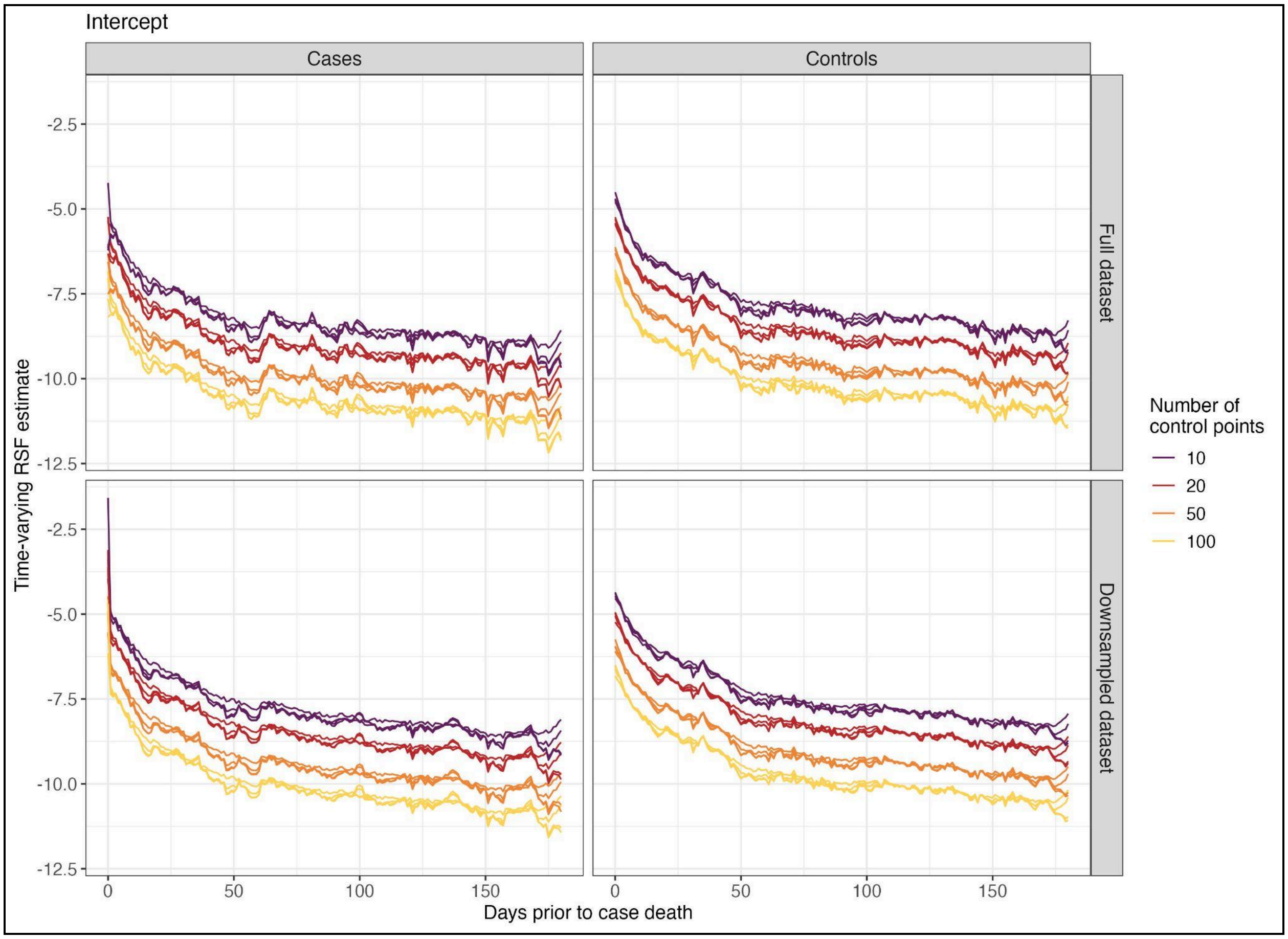

Figure S5: *The effect of time-varying resource selection function (RSF) settings on the resulting intercept estimates for white-tailed deer (*Odocoileus virginianus*) habitat selection in Wisconsin, USA. Each line represents estimates averaged across all deer per day prior to chronic wasting disease (CWD) case death. Lines are colored by the number of random control points drawn for each observed location. Lines of the same color vary by initial* Q *parameter settings. Each panel gives results by CWD status (case or control) and dataset, where the dataset is either the full, potentially autocorrelated movement dataset, or downsampled to a fix rate of every 12 hours (low or no autocorrelation).*

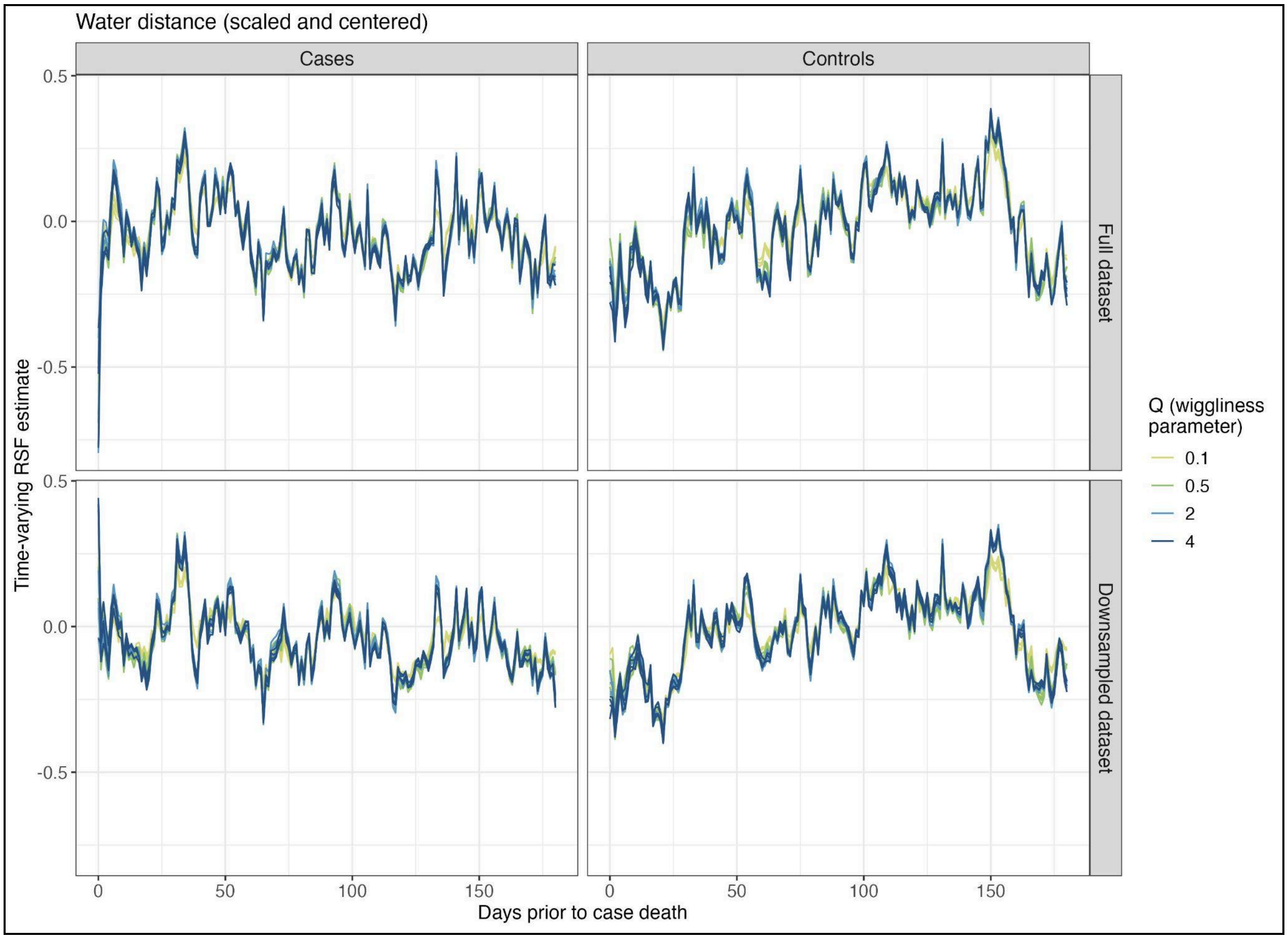

Figure S6: *The effect of time-varying resource selection function (RSF) settings on the resulting selection estimates for distance to nearest water source among white-tailed deer (*Odocoileus virginianus*) in Wisconsin, USA. Each line represents estimates averaged across all deer per day prior to chronic wasting disease (CWD) case death. Lines are colored by the number the initial* Q *parameter value. Lines of the same color vary by the number of random control points drawn for each observed location. Each panel gives results by CWD status (case or control) and dataset, where the dataset is either the full, potentially autocorrelated movement dataset, or downsampled to a fix rate of every 12 hours (low or no autocorrelation).*

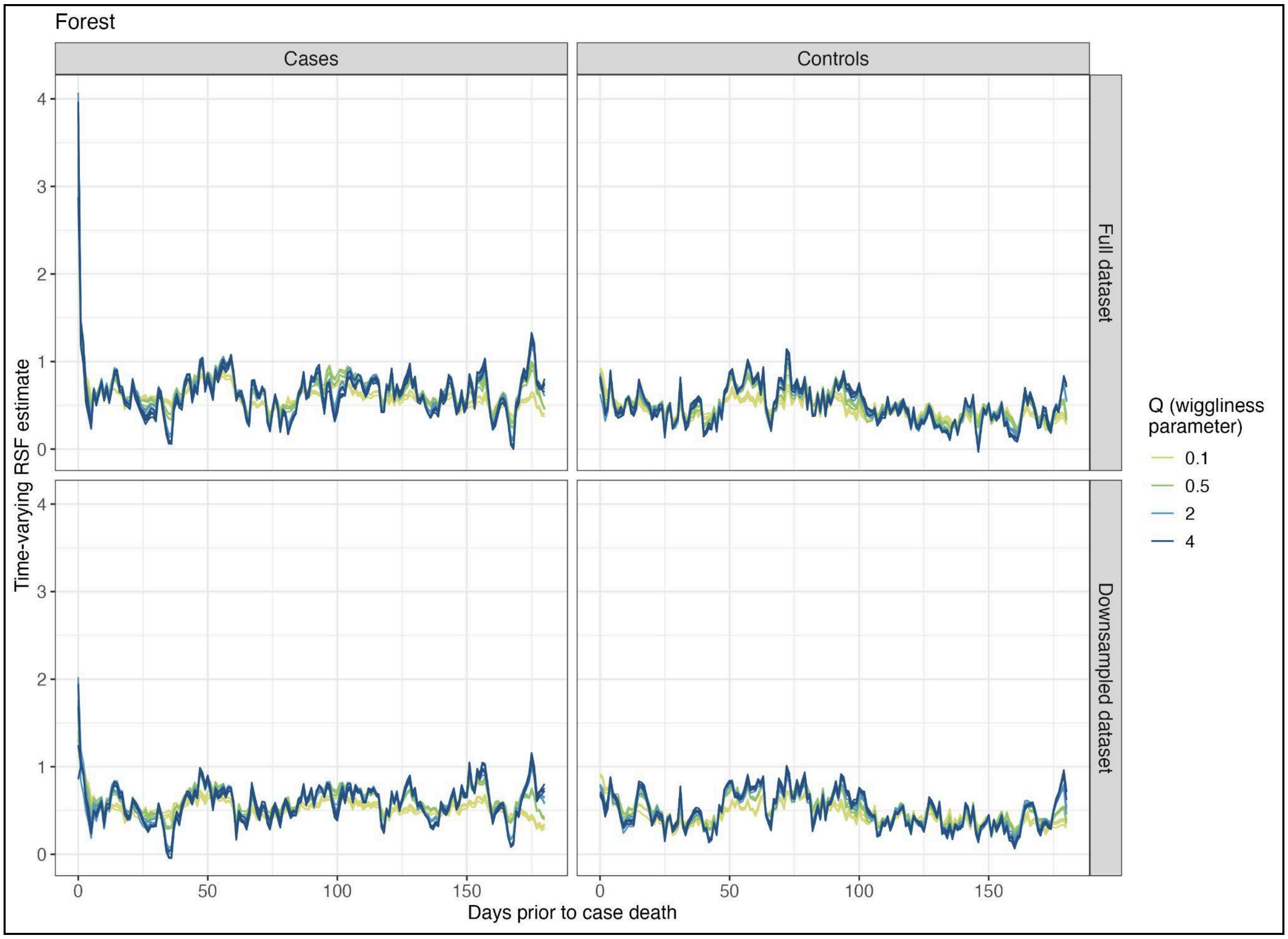

Figure S7: *The effect of time-varying resource selection function (RSF) settings on the resulting selection estimates for forested habitat among white-tailed deer (*Odocoileus virginianus*) in Wisconsin, USA. Each line represents estimates averaged across all deer per day prior to chronic wasting disease (CWD) case death. Lines are colored by the number the initial* Q *parameter value. Lines of the same color vary by the number of random control points drawn for each observed location. Each panel gives results by CWD status (case or control) and dataset, where the dataset is either the full, potentially autocorrelated movement dataset, or downsampled to a fix rate of every 12 hours (low or no autocorrelation).*

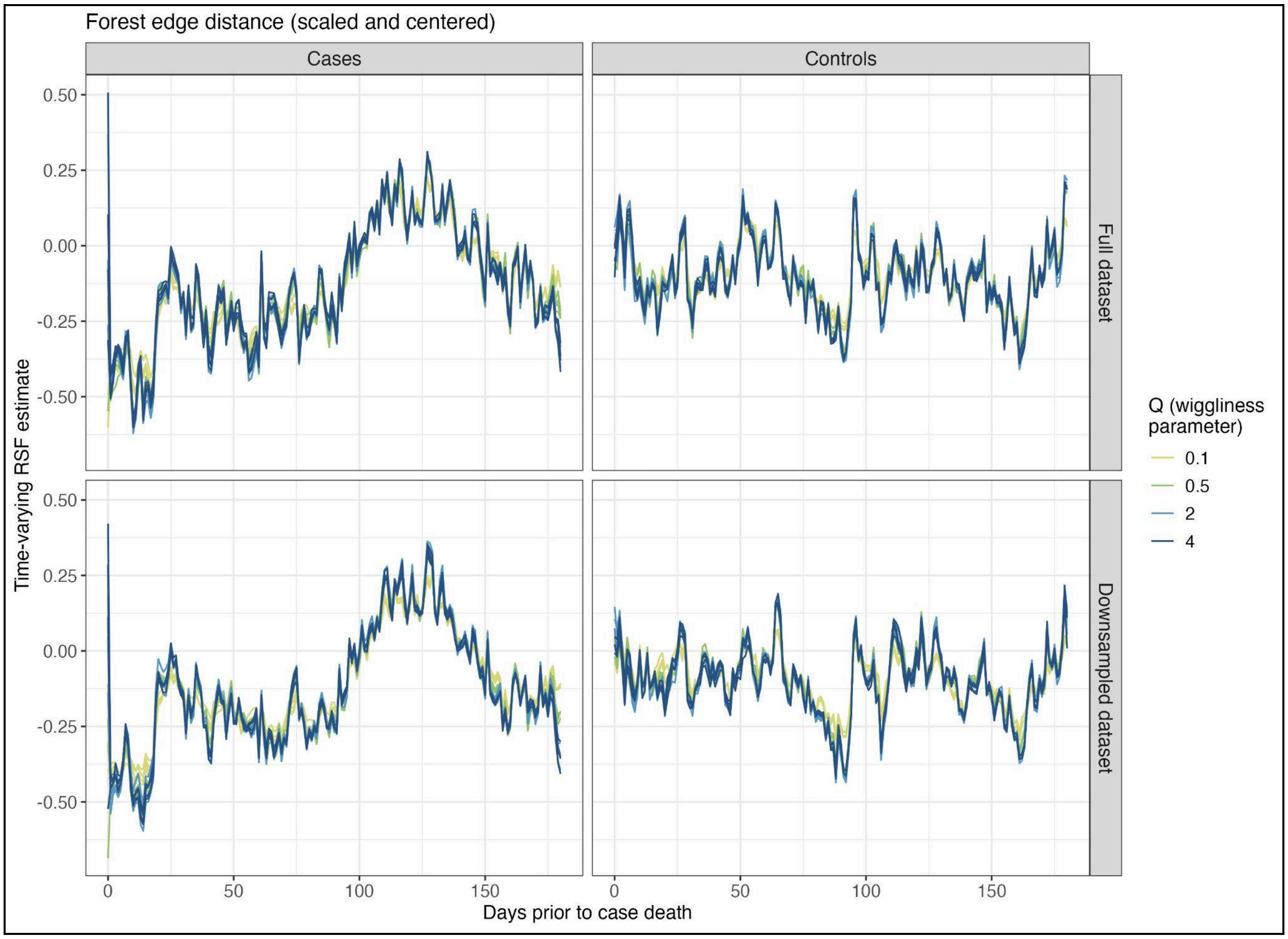

Figure S8: *The effect of time-varying resource selection function (RSF) settings on the resulting selection estimates for distance to nearest forest edge among white-tailed deer (*Odocoileus virginianus*) in Wisconsin, USA. Each line represents estimates averaged across all deer per day prior to chronic wasting disease (CWD) case death. Lines are colored by the number the initial* Q *parameter value. Lines of the same color vary by the number of random control points drawn for each observed location. Each panel gives results by CWD status (case or control) and dataset, where the dataset is either the full, potentially autocorrelated movement dataset, or downsampled to a fix rate of every 12 hours (low or no autocorrelation).*

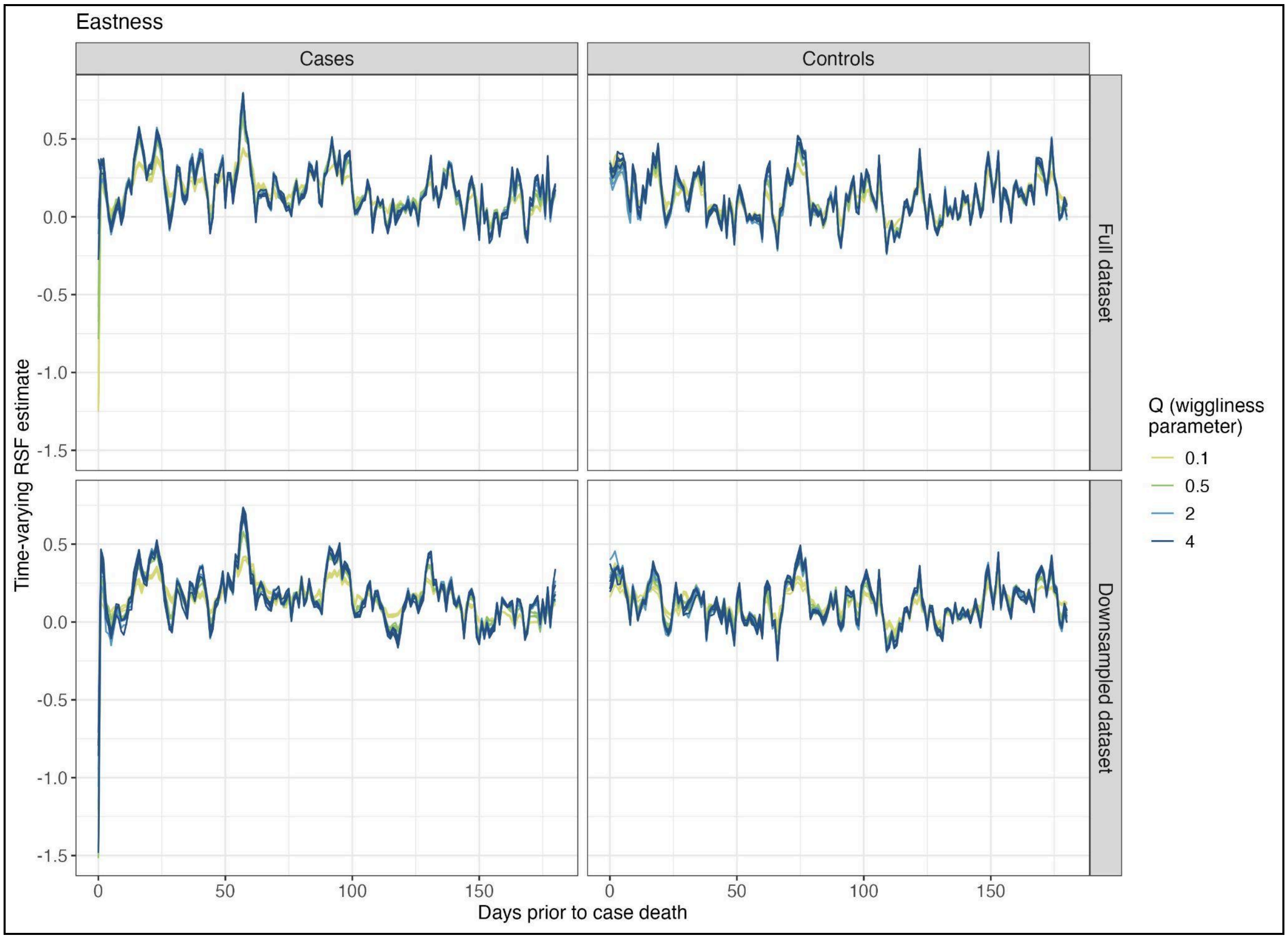

Figure S9: *The effect of time-varying resource selection function (RSF) settings on the resulting selection estimates for aspect eastness among white-tailed deer (*Odocoileus virginianus*) in Wisconsin, USA. Each line represents estimates averaged across all deer per day prior to chronic wasting disease (CWD) case death. Lines are colored by the number the initial* Q *parameter value. Lines of the same color vary by the number of random control points drawn for each observed location. Each panel gives results by CWD status (case or control) and dataset, where the dataset is either the full, potentially autocorrelated movement dataset, or downsampled to a fix rate of every 12 hours (low or no autocorrelation).*

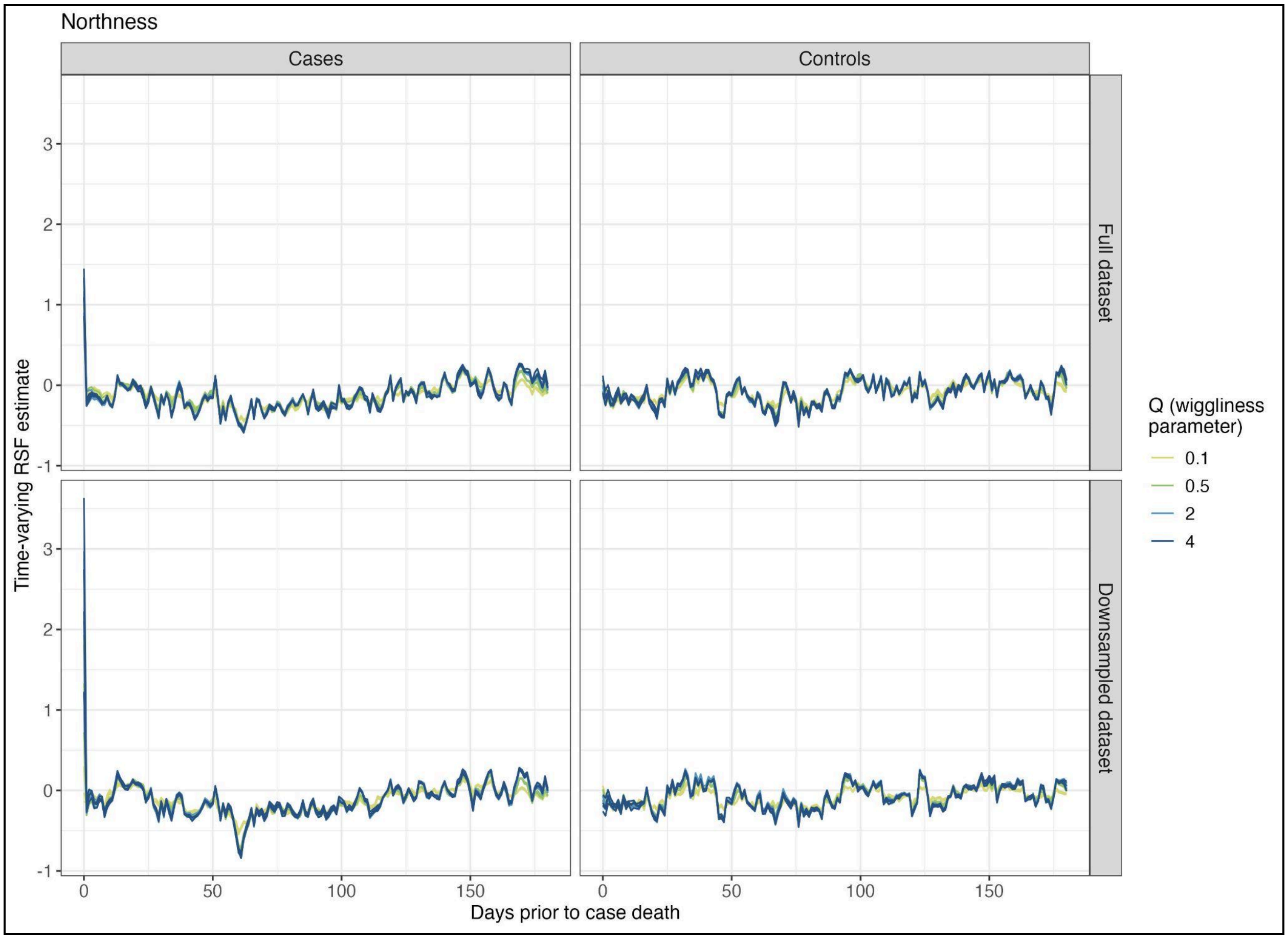

Figure S10: *The effect of time-varying resource selection function (RSF) settings on the resulting selection estimates for aspect northness among white-tailed deer (*Odocoileus virginianus*) in Wisconsin, USA. Each line represents estimates averaged across all deer per day prior to chronic wasting disease (CWD) case death. Lines are colored by the number the initial* Q *parameter value. Lines of the same color vary by the number of random control points drawn for each observed location. Each panel gives results by CWD status (case or control) and dataset, where the dataset is either the full, potentially autocorrelated movement dataset, or downsampled to a fix rate of every 12 hours (low or no autocorrelation).*

The intercept estimate was most affected by the number of control points selected for each random location, but all other parameters were relatively robust to this setting. This is apparent in Appendix S3: Figures S6-S10, where the different lines colored by the same *Q* parameter setting are largely indecipherable (i.e., overlapping) with each other. Habitat selection parameters were sensitive to the initial *Q* parameter setting, but this appeared to decrease as *Q* increased such that selection estimates when *Q* was set to 2 or 4 were largely consistent over the time series (i.e., light and dark blue lines are almost fully overlapping in Appendix S3: Figures S6-S10). These observations directed our decision to use tvRSF estimates from models with 100 control points for each observed location and an initial *Q* parameter of 2. This latter setting is

consistent with recommendations from Dejeante et al. (2024), who explored the effect of initial *Q* settings.

We also examined the Pearson correlation coefficients between selection estimates from the full and downsampled datasets (using 100 control points and initial $Q = 2$). With autocorrelated movement data, estimated standard errors are typically too low (i.e., excess Type I error; Alston et al. 2023); however, our objective in obtaining selection estimates from tvRSFs was not to make inference from those estimates, but to examine changes in the estimates over time. As such, our priority was to ensure that the selection estimates were robust to potential temporal autocorrelation (Appendix S3: Figure S11).

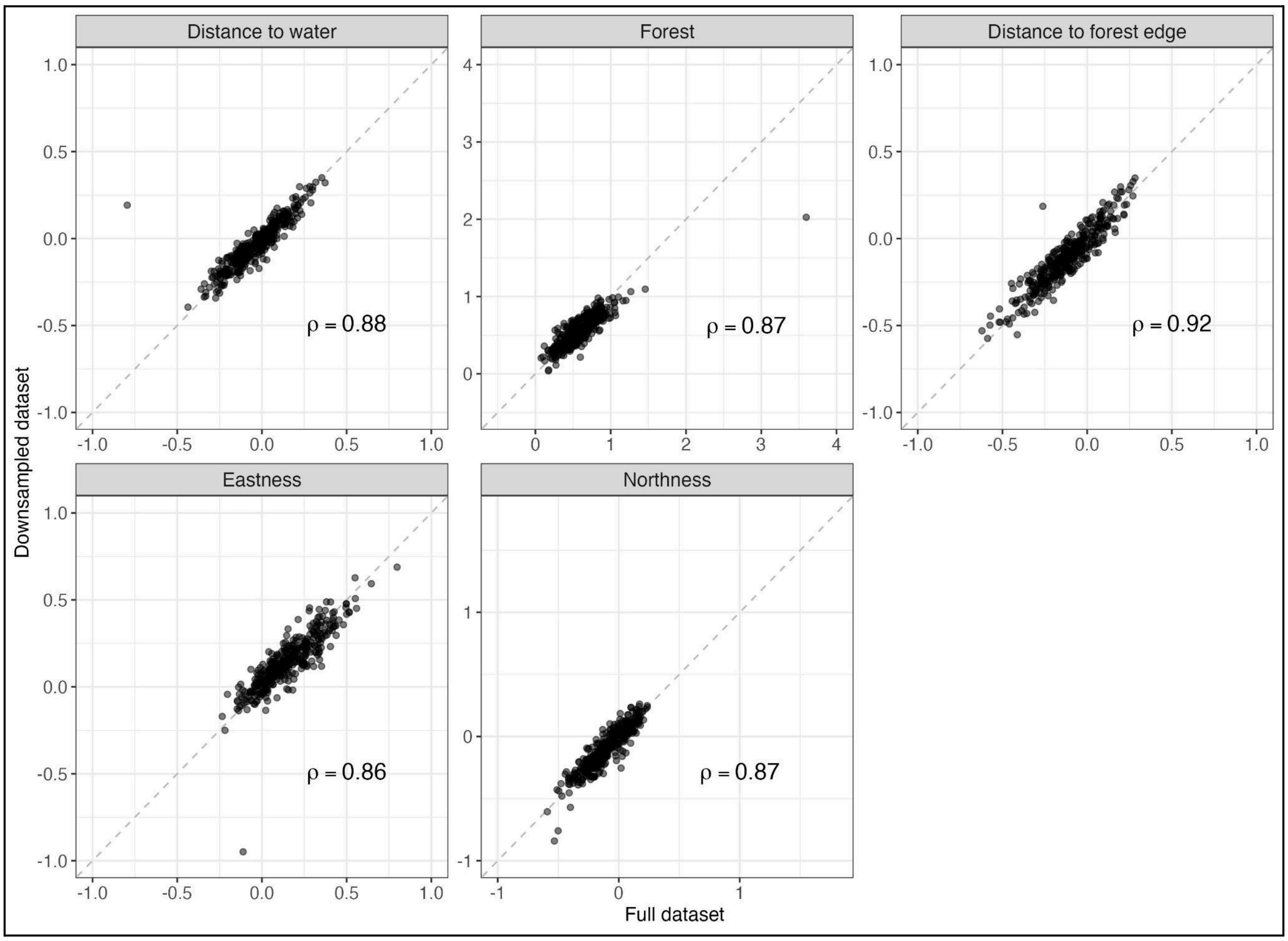

Figure S11: *Pearson correlation coefficients comparing time-varying habitat selection estimates from two different versions of a white-tailed deer (*Odocoileus virginianus*) movement dataset. The full dataset is potentially temporally autocorrelated, while the downsampled dataset has been reduced to a fix rate of every 12 hours for each individual (low to no autocorrelation). Points compare selection coefficient estimates when drawn from the same model, but the two different datasets. The dashed line is a 1:1 line; perfect agreement in estimates would fall precisely along the dashed line with a correlation coefficient (ρ) of 1.*

The tvRSF selection estimates from the full and downsampled datasets were highly correlated (Pearson ρ > 0.85) for all habitat covariates. As such, we concluded that our selection results were robust to any autocorrelation that may be present in the full dataset.

Section S5: Sensitivity of conditional logistic regression to sample size changes

Deer were monitored for different durations of time due to collar failure and animal death. In addition, temporal overlap within case-control pairs varied in duration, despite matching by Julian day rather than date. As a result, sample size (number of matched pairs) increased toward the end of the time series (Appendix S3: Figure S12). We extracted the subset of pairs with GPS collar data in all 6 months prior to case death (n = 17 pairs) and fitted the conditional logistic regression models described in the main text to determine if our results were robust to changing sample size over time. Results with this subset were consistent with those from the full dataset presented in the main text (Appendix S3: Figure S13).

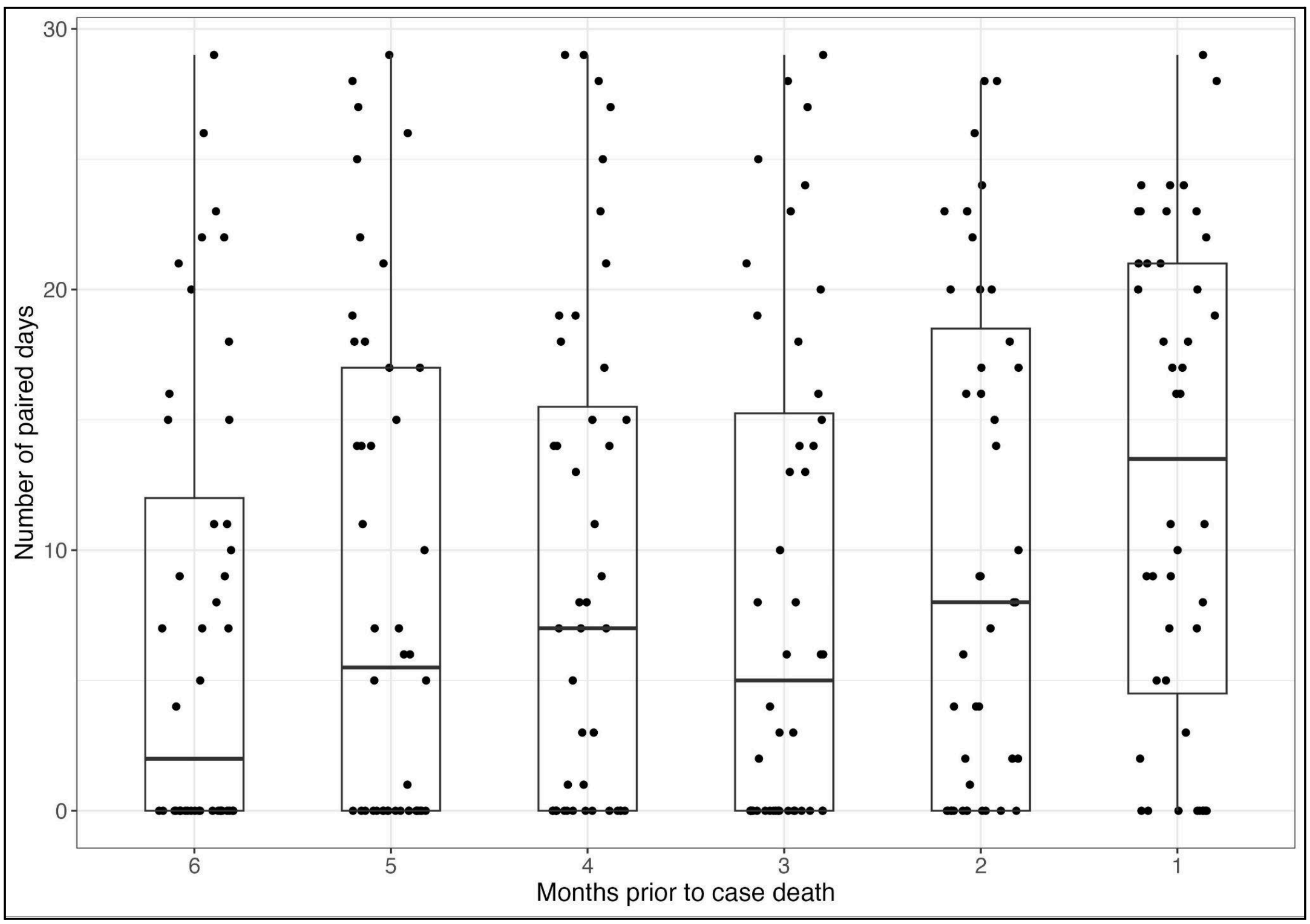

Figure S12: *Number of days with movement data within case-control pairs of Wisconsin, USA white-tailed deer (*Odocoileus virginianus*). Boxplots were calculated including pairs with no observations in a given month. Points are jittered in the x-axis for visibility.*

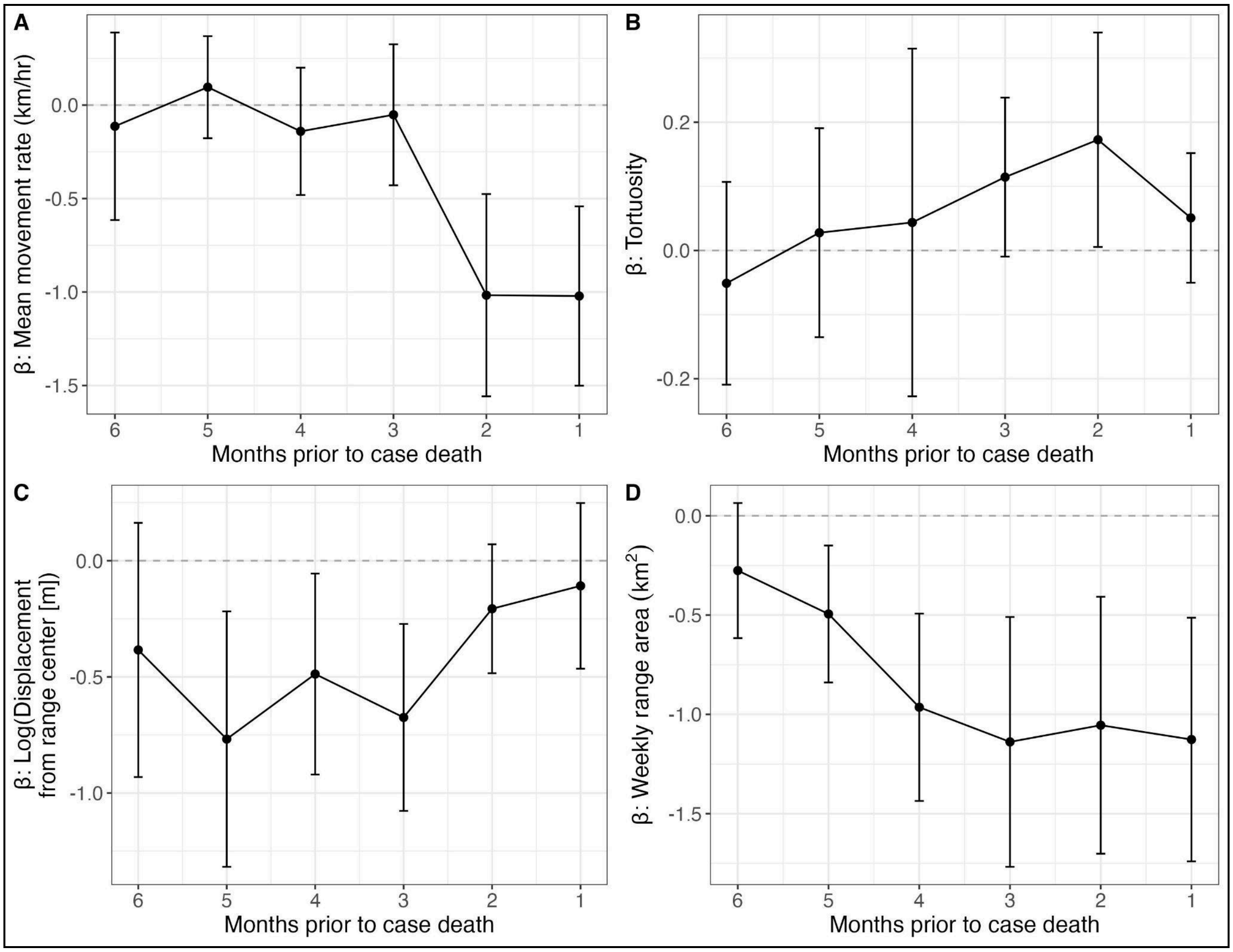

Figure S13: *The relationship between key movement characteristics and chronic wasting disease (CWD) status for white-tailed deer (*Odocoileus virginianus*) in southwest Wisconsin, USA, as quantified by conditional logistic regression. Models were fit with the subset of pairs that had movement data for all six months prior to case death. The response variable was CWD case or control status, so the y-axis gives parameter estimates for the effect of each movement characteristic (β) on CWD status. A separate model was fit for each month prior to case death, so points represent the estimate from a given model, and bars are the 95% confidence intervals. An estimate of zero indicates no relationship between a metric and CWD status; a negative value indicates case status was associated with lower metric values than controls.*

<u>Section S6: Supporting references</u>

Alston, J. M., C. H. Fleming, R. Kays, J. P. Streicher, C. T. Downs, T. Ramesh, B. Reineking, and J. M. Calabrese. 2023. Mitigating pseudoreplication and bias in resource selection functions with autocorrelation‐informed weighting. *Methods in Ecology and Evolution* 14:643–654.

Dejeante, R., M. Valeix, and S. Chamaillé-Jammes. 2024. Time-varying habitat selection

analysis: A model and applications for studying diel, seasonal, and post-release changes. *Ecology* 105:e4233.

McClintock, B. T., and T. Michelot. 2018. momentuHMM: R package for generalized hidden Markov models of animal movement. *Methods in Ecology and Evolution* 9:1518-1530.

R Core Team. 2024. R: A Language and Environment for Statistical Computing. R Foundation for Statistical Computing, Vienna, Austria.
