# Supplementary material for: Malady in motion: Effects of chronic wasting disease on host movement
Source: Ecol Appl. 2026 Oct 23;36(7):e70338. doi: 10.1002/eap.70338 (PMC13694198; doi:10.1002/eap.70338)
Supplement: Supplementary file 2 [file EAP-36-e70338-s002.pdf]

APPENDIX S1: SUPPLEMENTARY METHODS

Journal: ***Ecological Applications***

Malady in motion: Effects of chronic wasting disease on host movement

Marie L. J. Gilbertson, Daniel P. Walsh, Daniel J. Storm, Allen Herbst, Wendy C. Turner

Any use of trade, product, or firm names is for descriptive purposes only and does not imply endorsement by the U.S. Government.

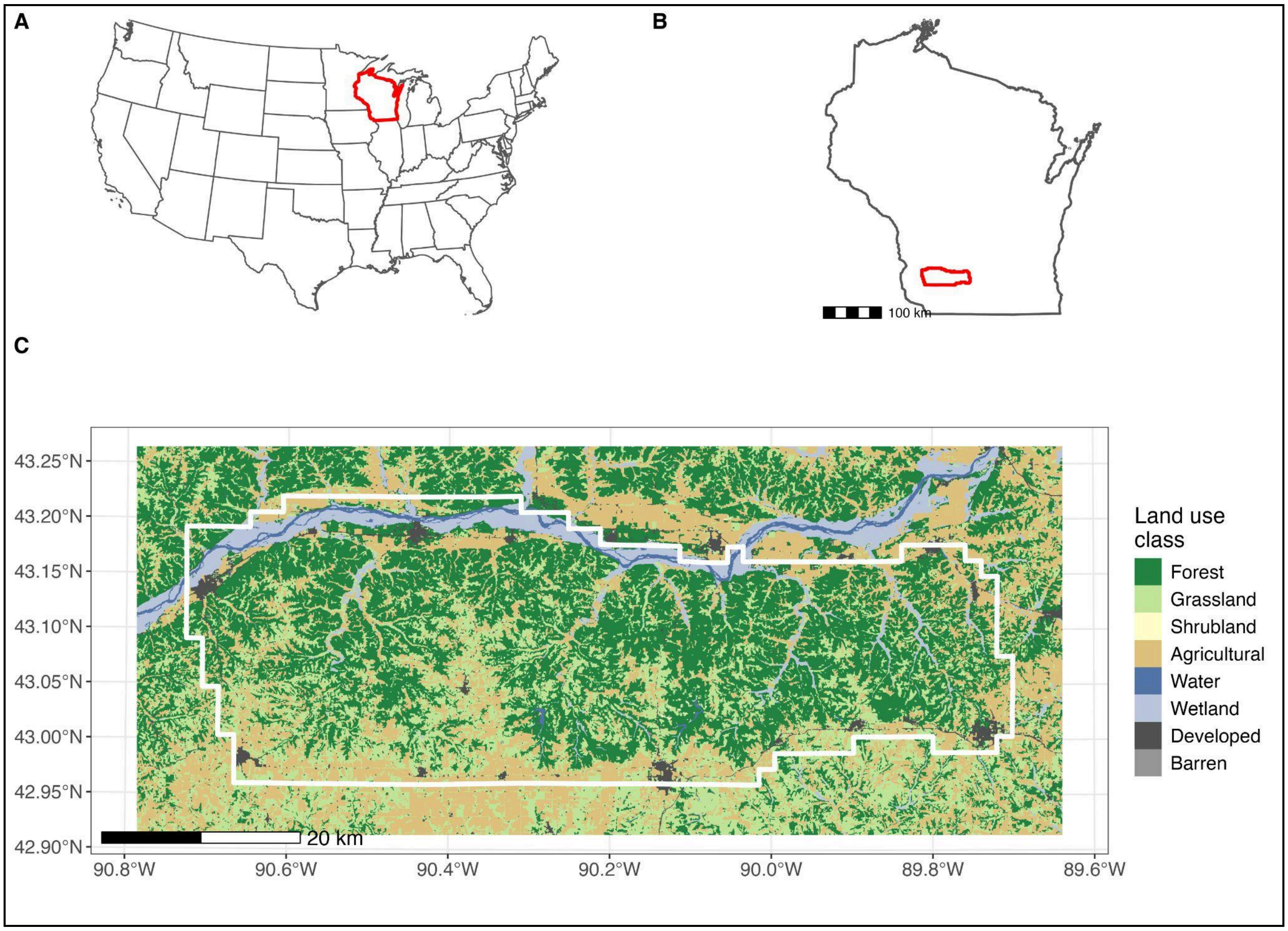

Figure S1: Maps of study area in southwest Wisconsin, USA. Map (A) shows Wisconsin outlined in red within the context of the continental United States. In (B), the study area is outlined in red within the state of Wisconsin. In (C), the study area outline is given in white, with the background landscape colored by land use designations from the Wiscland2 database (Appendix S1: Table S3). Note that animals were all captured within the study area outline, but could disperse outside of this area.

Table S1: Methods overview and sample sizes for analyses of white-tailed deer (*Odocoileus virginianus*) chronic wasting disease (CWD) status, condition, movement, and habitat selection in southwest Wisconsin, USA.

Figure 1 mapping	Description	Matched data?	# individuals	# CWD positive
End stage CWD cases and age-, sex-, and time-matched controls	Maximum number of CWD cases matched with a control individual; all analyses with matched data use a subset of this dataset	Y	88	44
Does body condition differ between CWD cases and matched controls? If so, when?	Analysis of body condition at capture, CWD status, and time between capture and death	Y	80	40
Does excursion behavior differ between CWD cases and matched controls? Subquestions: Occurrence? Occurrence over time?	Analysis of occurrence of excursions (ever or on a monthly basis) and CWD status	Y	81	40
Does excursion behavior differ between CWD cases and matched controls? Subquestion: Distance?	Analysis of excursion distance; only includes animals who made at least one excursion	Y*	53	26
Does excursion behavior differ between CWD cases and matched controls? Subquestion: Duration?	Analysis of excursion distance; only includes animals who made at least one excursion	Y*	53	26
Do movement characteristics and habitat selection differ between CWD cases and matched controls? Subquestion: Movement characteristics?	Analysis of CWD status as a function of movement characteristics; data are clustered such that a case is directly compared to their matched control	Y	80	40
Do movement characteristics and habitat selection differ between CWD cases and matched controls? Subquestion: Time-varying habitat selection?	Analysis of CWD status as a function of habitat selection; data are clustered such that a case is directly compared to their matched control	Y	78	39
When do changes in movement characteristics and habitat selection occur in CWD cases? Subquestion: Movement characteristics?	Analysis of the time at which movement characteristic change for CWD cases versus controls	Y	80	40

When do changes in movement and habitat selection occur in CWD cases? Subquestion: Habitat selection?	Analysis of the time at which habitat selection changes for CWD cases versus controls	Y	78	39
All individuals with known post-mortem CWD status and mortality location	Descriptive statistics for distance to nearest water source at time of death for all CWD positive and test-negative individuals with a recorded mortality location	N	251	110
Polydipsia: Do CWD cases die in closer proximity to water than controls?	Analysis of distance to nearest water source at time of death for CWD positive and test-negative individuals with recorded post-mortem body condition	N	163	80

*Note: Column "Figure 1 mapping" provides color coding and text matching those used in Figure 1 in the main text. Columns "# individuals" and "# CWD positive" give sample sizes for total individuals and those that were chronic wasting disease (CWD) positive, respectively. Y = an analysis used a subset of the matched dataset (described in the first row); N = an analysis did not use a subset of the matched dataset. * = these analyses used a subset of the matched data, but because inclusion depended on having made an excursion, both individuals in a matched case-control pair were not necessarily included in the analysis. Differences in sample sizes for the analyses using subsets of the matched data arose from differences in data availability.*

Table S2: Pairwise correlations and univariate model selection for movement characteristics among white-tailed deer (*Odocoileus virginianus*) in southwest Wisconsin, USA.

	Mean movement rate	Movement rate variance	Distance traveled	Tortuosity	Log(Mean displacement)
**Mean movement rate**	AIC = 11,444.1	**0.64**	**0.91**	0.15	0.46
Movement rate variance		AIC = 11,603.6	0.57	0.07	0.26
Distance traveled			AIC = 11,462.2	0.17	0.51
**Tortuosity**				AIC = 11,687.7	0.03
**Log(Mean displacement)**					AIC = 11,385.7

Note: Movement characteristics were calculated daily and were evaluated for use in conditional logistic regressions. Cells along the diagonal give Akaike information criterion (AIC) for univariate conditional logistic regressions. All other values are Pearson correlation coefficient estimates. Correlations greater than 0.6 were considered strongly correlated and are highlighted in bold. Among correlated movement characteristics, we selected the one(s) with the lowest AIC values for use in full conditional logistic regression analysis. These final movement characteristics are given by the bold row names.

Section S1: Habitat selection control point selection

Our primary interest for our habitat selection analysis was in determining how selection varied within an animal's immediate environment, based on chronic wasting disease (CWD) status. We therefore drew our available points from the 90% isopleth of each individual's occurrence distribution for the six-month observation period (as estimated using Brownian Bridge continuous time movement models; Calabrese et al. 2016). Some individuals, however, completed range shift events during this observation period. To ensure that selection was based only on habitat immediately available to an individual in a given time period, we allowed the "available" space to vary with time. Each 90% range isopleth was composed of one or more polygons; we determined the span of days that an individual used each polygon. For a given observed point, we then drew available points only from those polygons whose span of use overlapped the used point's time stamp.

Section S2: Habitat data

Original sources for all habitat layers are provided in Appendix S1: Table S3. The "forested versus other" habitat layer was derived from the Wiscland2 land use classification layer, with all 4200-level (broad-leaved deciduous forest) classes considered "forested" and all other classes considered "other." See Appendix S1: Figure S2 for the distribution of non-forested habitat types, relative to forested, among used deer locations.

The "distance to nearest forest edge" layer was then derived from a forested habitat raster that included all 4000-level forest (i.e., any forest, rather than just broad-leaved deciduous). We used the boundaries() function in the R *terra* package (R version 4.4.0; R Core Team 2024, Hijmans 2026) to detect forest edges (considering all 8 cells surrounding a given cell, i.e., Queen's case, and outer boundaries). We determined the distance to the nearest forest edge using the *FNN* package (Beygelzimer et al. 2022).

Aspect (in radians) was calculated from an elevation raster using the *terra* package. We converted radians to compass-oriented degrees, and then decomposed aspect into its "eastness" and "northness" components.

Distance to the nearest water source was derived from the combination of river/stream and water body layers (Appendix S1: Table S3). For rivers/streams, we included all perennial flows (hydrocodes 100, 101, 200, 201) and river/stream centerlines, stream extensions, and river/stream single-lines (hydrotypes 503, 506, 508). Similarly, we included all perennial water bodies (hydrocodes 6011, 6021, 011, 7021, 7031, 7051, 7061, 7071, 7081, 7091). We used the "distance accumulation" tool in ArcGIS to calculate the distance to the nearest water source.

Table S3: Data layers and sources used in study of white-tailed deer (*Odocoileus virginianus*) movement in southwest Wisconsin, USA.

Habitat layer	Source	Link	Date accessed
Land use classification	Wiscland2 from Wisconsin DNR Open Data	https://data-wi-dnr.opendata.arcgis.com/documents/wi-dnr::wiscland-2/about	May 09, 2024
Elevation	30 meter digital elevation model from Wisconsin DNR Open Data	https://data-wi-dnr.opendata.arcgis.com/documents/wi-dnr::digital-elevation-model-dem-30-meter/about	Aug 10, 2024
Rivers/Streams	24k hydro flowlines (rivers/streams) from Wisconsin DNR Open Data	https://data-wi-dnr.opendata.arcgis.com/datasets/wi-dnr::24k-hydro-flowlines-rivers-streams/about	May 09, 2024

Water bodies	24k hydro waterbodies (open water) from Wisconsin DNR Open Data	https://data-wi-dnr.opendata.arcgis.com/datasets/wi-dnr::24k-hydro-waterbodies-open-water/about	May 09, 2024

Note: "DNR" = Department of Natural Resources

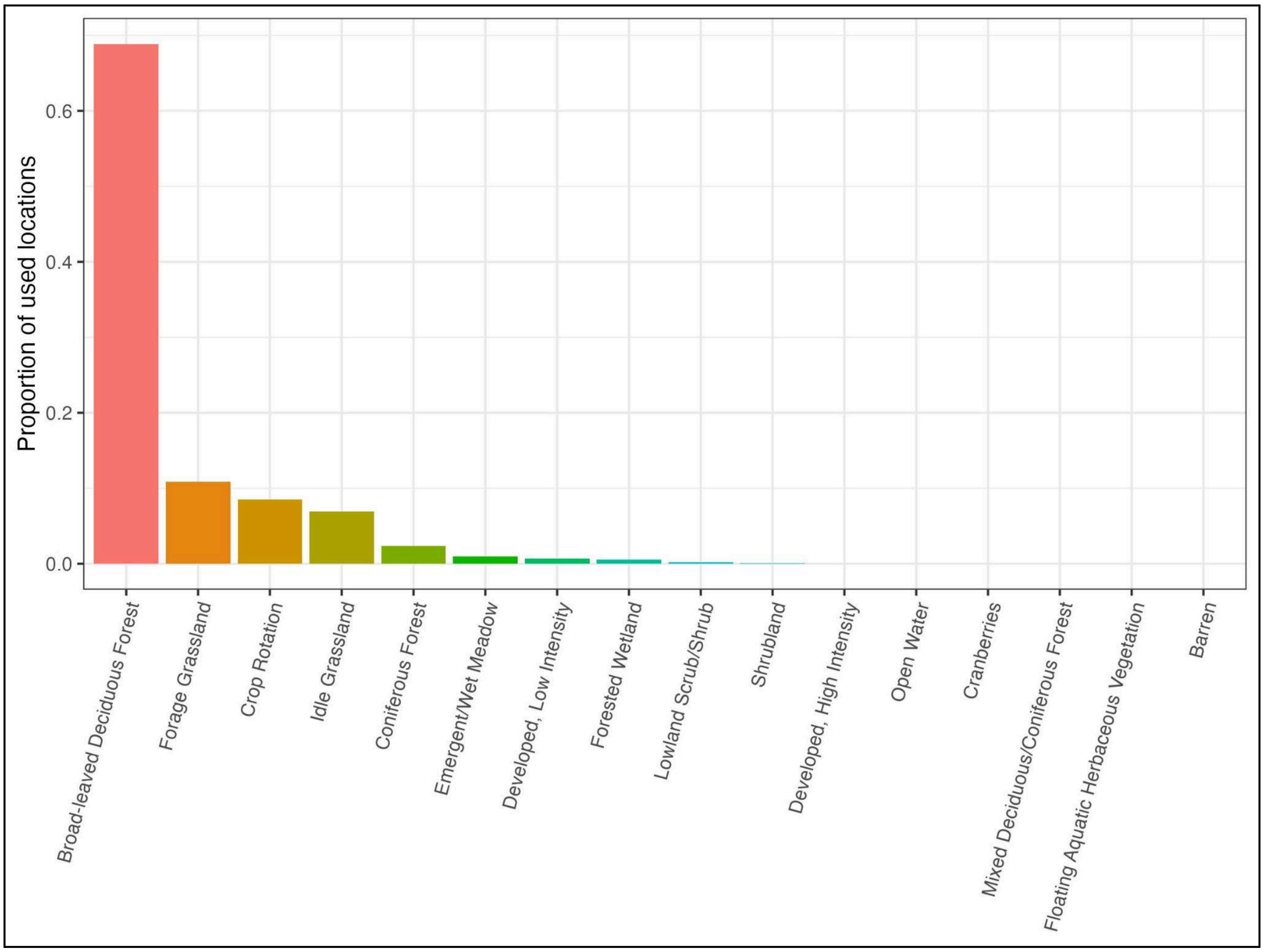

Figure S2: Distribution of habitat types used by white-tailed deer (*Odocoileus virginianus*) in southwest Wisconsin, USA. Note that the majority of broad-leaved deciduous forest is further classified as "central hardwoods." All non-broad-leaved deciduous forest habitat classes were merged into a single "other" category for habitat selection analysis.

Section S3: Change point analysis

Table S4: Data transformations for southwest Wisconsin white-tailed deer (*Odocoileus virginianus*) movement characteristics evaluated in change point analysis

**Response variable**	**Transformation**
Movement rate	Square root-transformed
Displacement from range center	Log-transformed
Tortuosity*	Scaled and centered, added a constant (0.01), and log-transformed
Weekly range area	Log_{10}-transformed

*Note: Transformations were performed to meet assumptions of normality for change point analysis. *Tortuosity was extremely skewed and truncated, so several transformations were needed to meet assumptions of normality. Because the objective of change point analysis was to identify the presence and timing of any change points in movement characteristics—rather than retaining interpretability of coefficient estimates—an extreme transformation was considered acceptable.*

Table S5: Informative Gaussian priors for change point analysis of movement and habitat selection data from white-tailed deer (*Odocoileus virginianus*) in southwest Wisconsin, USA.

**Response variable**	**Change point prior: mean(sd)***
Movement rate	120(15)
Displacement from range center	120(15)
Tortuosity	120(15)
Weekly range size[†]	4(2)
Selection for distance to water	120(15)
Selection for forested habitat	90(15)
Selection for distance to forest edges	120(15)
Selection for easterly aspects	30(15)
Selection for northerly aspects	60(15)

*Note: *Gaussian priors for the timing of the change point, with mean based on apparent change points from conditional logistic regressions (described in main text) and standard deviations (sd) provided in parentheses. Change point prior means represent days into the time series (e.g., the prior for the change point for selection for easterly aspects is centered on 30 days into the time series, or about 150 days prior to case death). †Weekly range area is an exception, where units are in weeks rather than days.*

Section S4: Supporting references

Beygelzimer, A., S. Kakadet, J. Langford, S. Arya, D. Mount, and S. Li. 2022. FNN: Fast Nearest Neighbor Search Algorithms and Applications.

Calabrese, J. M., C. H. Fleming, and E. Gurarie. 2016. Ctmm: An r package for analyzing animal relocation data as a continuous‐time stochastic process. *Methods in ecology and evolution* 7:1124–1132.

Hijmans, R. J. 2026. terra: Spatial Data Analysis.

R Core Team. 2024. R: A Language and Environment for Statistical Computing. R Foundation for Statistical Computing, Vienna, Austria.
