# Supplementary material for: Malady in motion: Effects of chronic wasting disease on host movement
Source: Ecol Appl. 2026 Oct 23;36(7):e70338. doi: 10.1002/eap.70338 (PMC13694198; doi:10.1002/eap.70338)
Supplement: Supplementary file 3 [file EAP-36-e70338-s003.pdf]

APPENDIX S2: SUPPLEMENTARY RESULTS

Journal: ***Ecological Applications***

Malady in motion: Effects of chronic wasting disease on host movement

Marie L. J. Gilbertson, Daniel P. Walsh, Daniel J. Storm, Allen Herbst, Wendy C. Turner

Any use of trade, product, or firm names is for descriptive purposes only and does not imply endorsement by the U.S. Government.

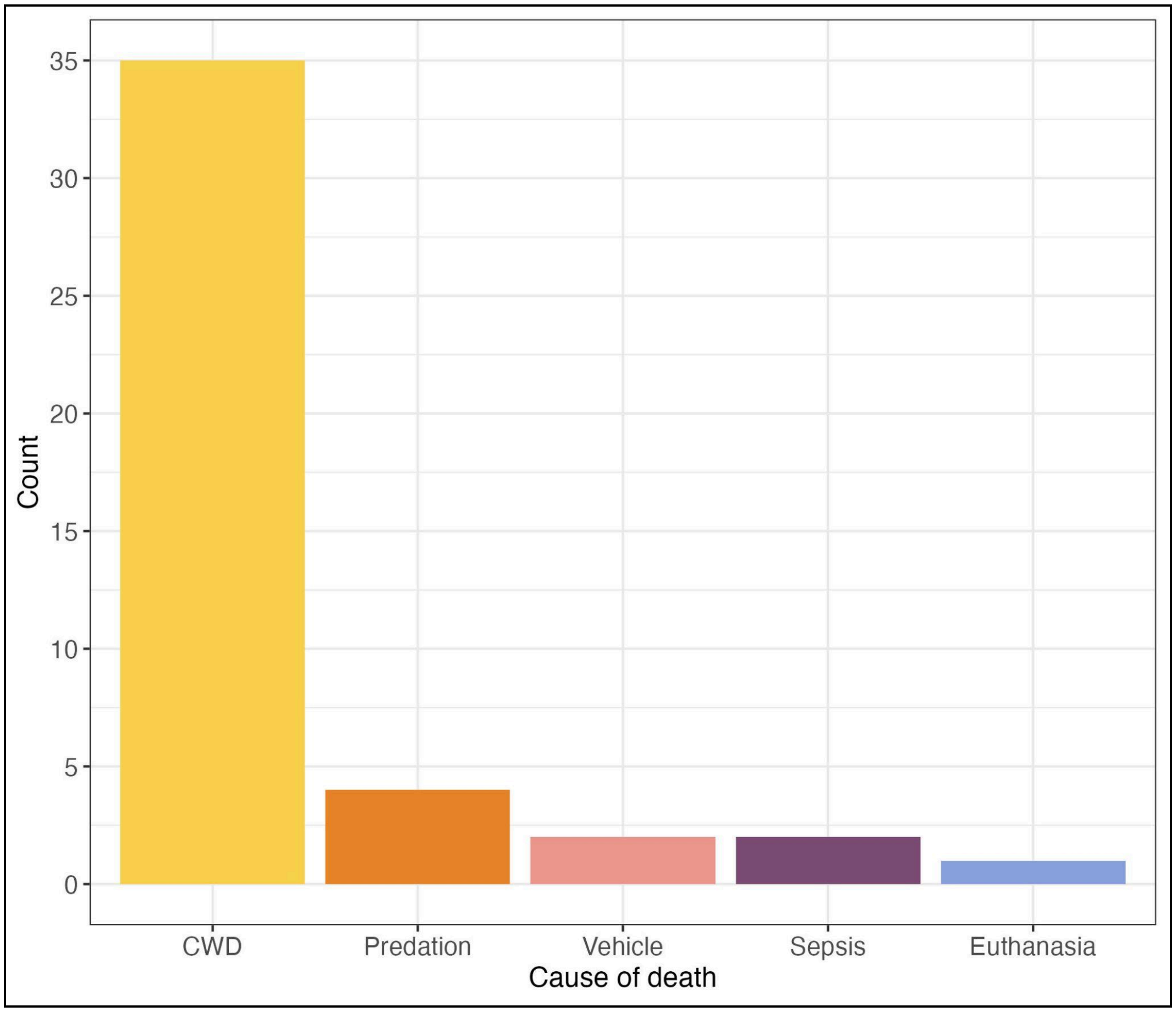

Figure S1: *Cause of death among white-tailed deer (*Odocoileus virginianus*) in end-stage chronic wasting disease (CWD) evaluated in this study from southwest Wisconsin, USA. Causes of death were determined from field and laboratory necropsies; for individuals whose cause of death is listed as CWD, necropsy directly attributed the animal's death to CWD through starvation with or without pneumonia.*

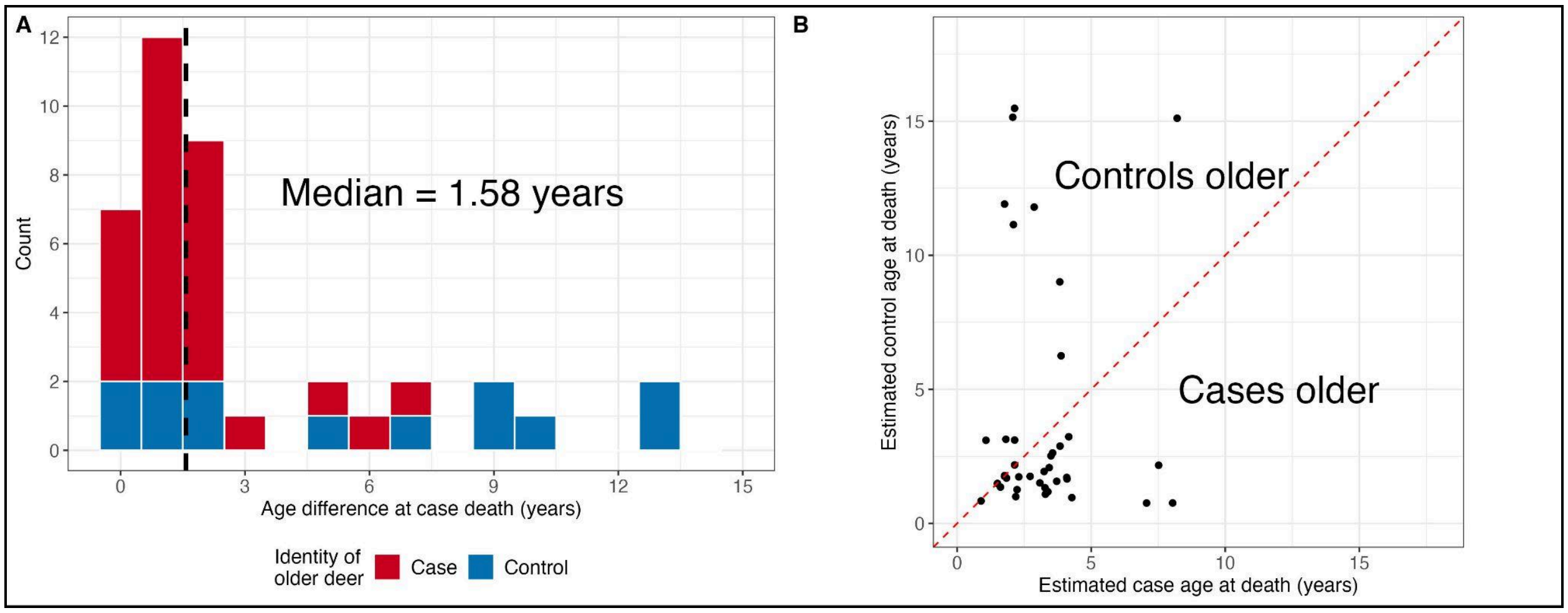

Figure S2: *Differences in ages at time of case death within matched pairs of white-tailed deer (*Odocoileus virginianus*) in southwest Wisconsin, USA. A) Distribution of age differences, with the vertical dashed line giving the median age difference within pairs. Bars are colored by the identity of the older individual within pairs. B) Scatterplot of case versus control ages within pairs; the dashed red line is a 1:1 line, so perfectly matched ages would fall along this line. Points above the 1:1 line correspond to pairs where the control was older than the case; below the line correspond to pairs where the case was older.*

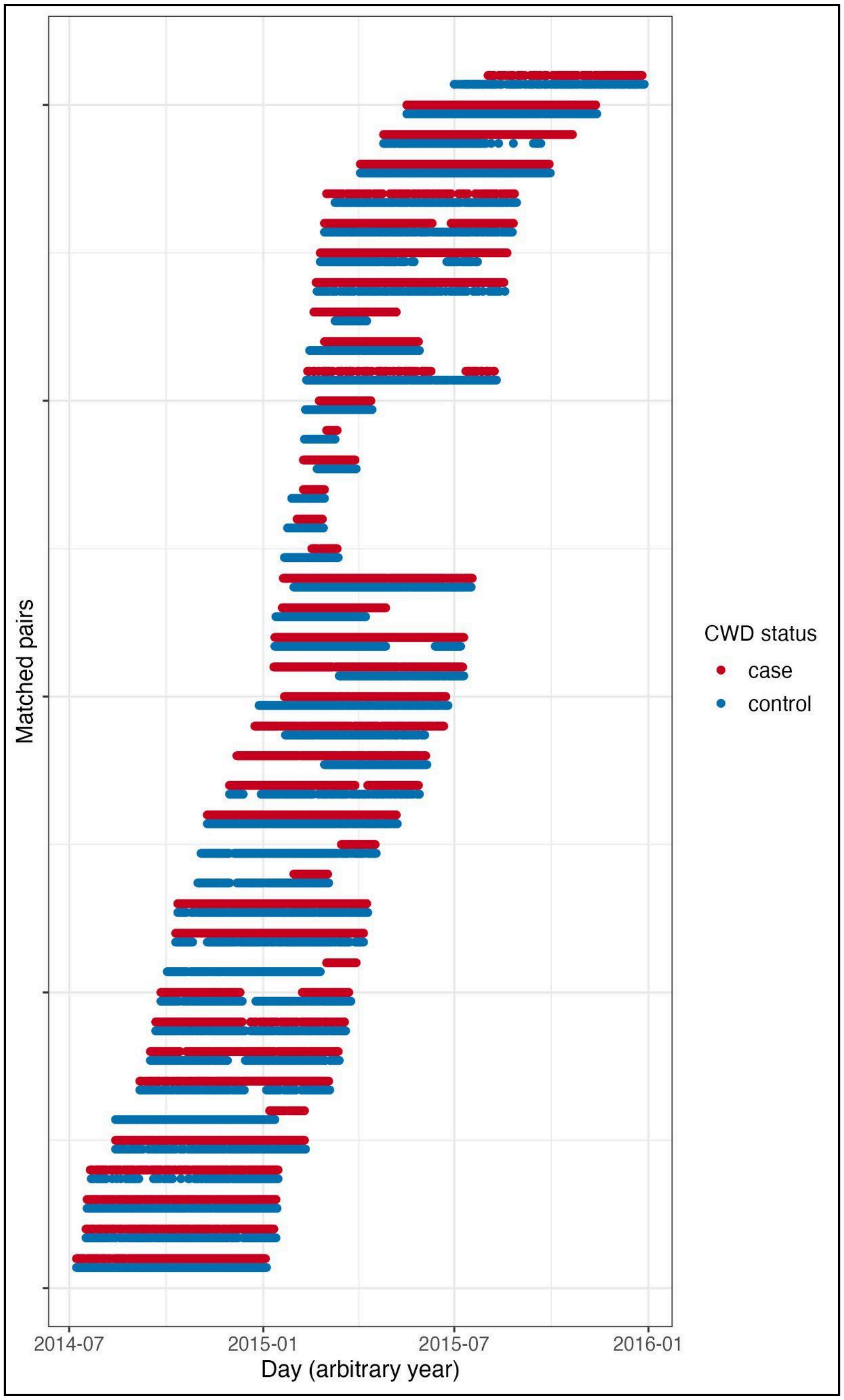

Figure S3: *Matching of case-control paired white-tailed deer (*Odocoileus virginianus*) in time in southwest Wisconsin, USA. Cases are those deer that tested positive for chronic wasting disease (CWD), and are shown in red. The control matched with a given case is shown in blue directly beneath the corresponding case. Pairs were matched by Julian day, so the year shown is arbitrary.*

Table S1: Candidate cumulative logistic regressions for body condition score at capture among among white-tailed deer (*Odocoileus virginianus*) in southwest Wisconsin, USA

Model	ΔAIC
**CWD status*days prior to death + age class + sex**	**0**
CWD status*days prior to death + age class + genotype	2.4
CWD status*days prior to death	8.9

Note: CWD = Chronic wasting disease; AIC = Akaike information criterion. The top model is highlighted in bold.

Table S2: Cumulative logit regression results for body condition score at capture among white-tailed deer (*Odocoileus virginianus*) in southwest Wisconsin, USA. Body condition scores were integers ranging from 0 (low) to 10 (high).

Predictor	Odds	95% CI
**Intercept: 1**	**0.05**	**(0.01 - 0.18)**
**Intercept: 2**	**0.21**	**(0.09 - 0.48)**
**Intercept: 3**	**0.46**	**(0.22 - 0.96)**
Intercept: 4	0.78	(0.38 - 1.6)
Intercept: 5	1.61	(0.77 - 3.35)
**Intercept: 6**	**2.47**	**(1.16 - 5.26)**
**Intercept: 7**	**4.74**	**(2.12 - 10.61)**
**Intercept: 8**	**9.96**	**(4.11 - 24.16)**
**Intercept: 9**	**24.05**	**(8.49 - 68.14)**
**Intercept: 10**	**93.77**	**(19.47 - 451.66)**
CWD status: Case	1.45	(0.65 - 3.21)
Days prior to death*	0.94	(0.52 - 1.71)
**Age class: Subadult**	**0.21**	**(0.07 - 0.62)**
**Age class: Adult**	**0.18**	**(0.07 - 0.52)**
Sex: Male	1.63	(0.51 - 5.18)
Case:Days prior to death*	1.11	(0.48 - 2.57)

**Scaled and centered. Statistically significant results are given in bold text. 95% CI = 95% confidence interval; CWD = Chronic wasting disease (status determined at death). Reference category for CWD status was controls; for age class was juveniles; for sex was females.*

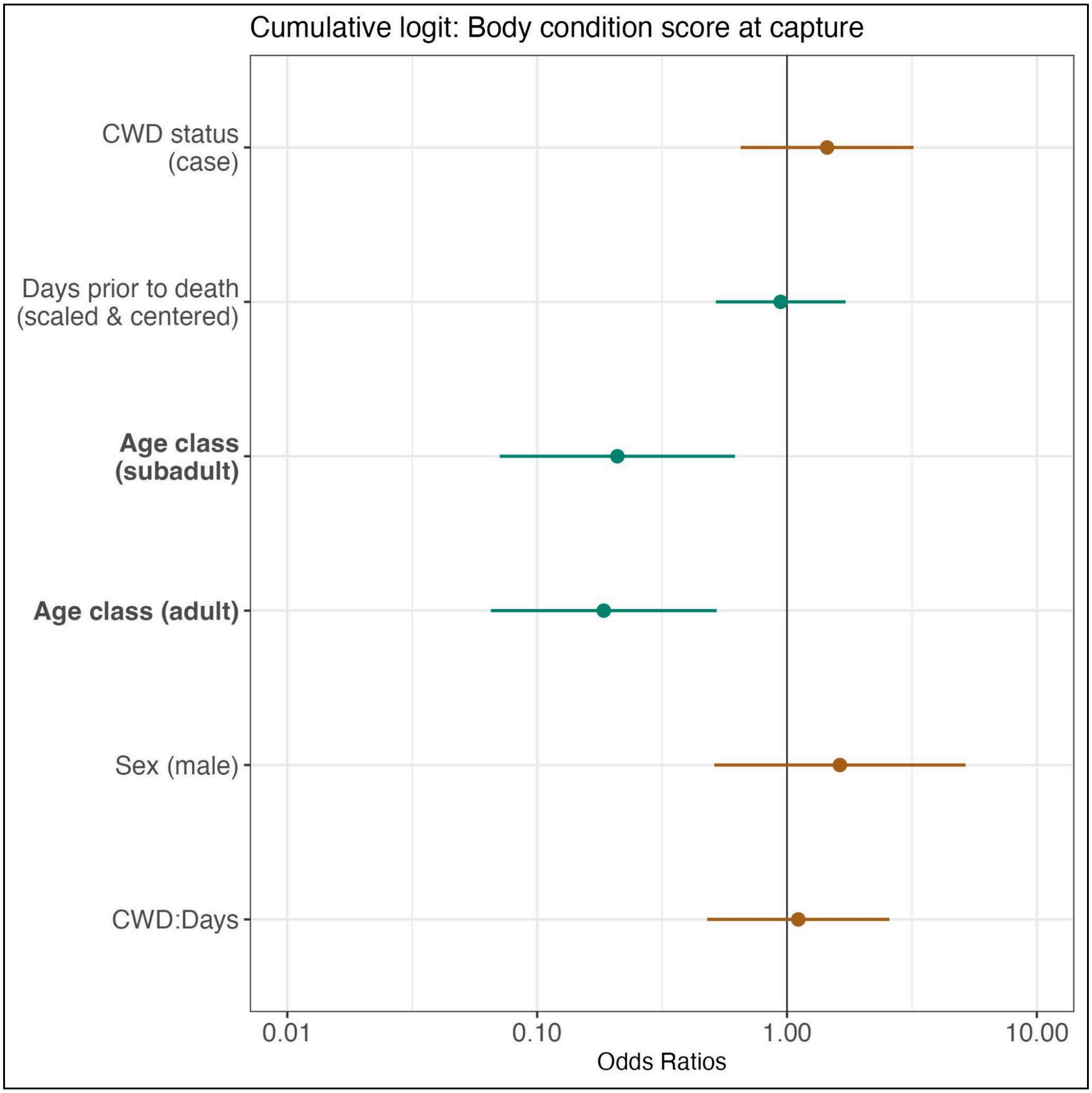

Figure S4: *Cumulative logit regression estimates (points) and 95% confidence intervals (horizontal lines) for body condition score at capture among white-tailed deer (*Odocoileus virginianus*) in southwest Wisconsin, USA. Body condition scores were integers ranging from 0 (low) to 10 (high). Statistically significant results are given in bold text. Color indicates direction of relationship, with teal values representing odds ratios less than one, and tan values greater than one. Note that interpretation of estimates from cumulative logit regressions can seem counterintuitive: an odds ratio less than one is associated with increased body condition score. CWD = chronic wasting disease. Reference category for CWD status was controls; for age class was juveniles; for sex was females.*

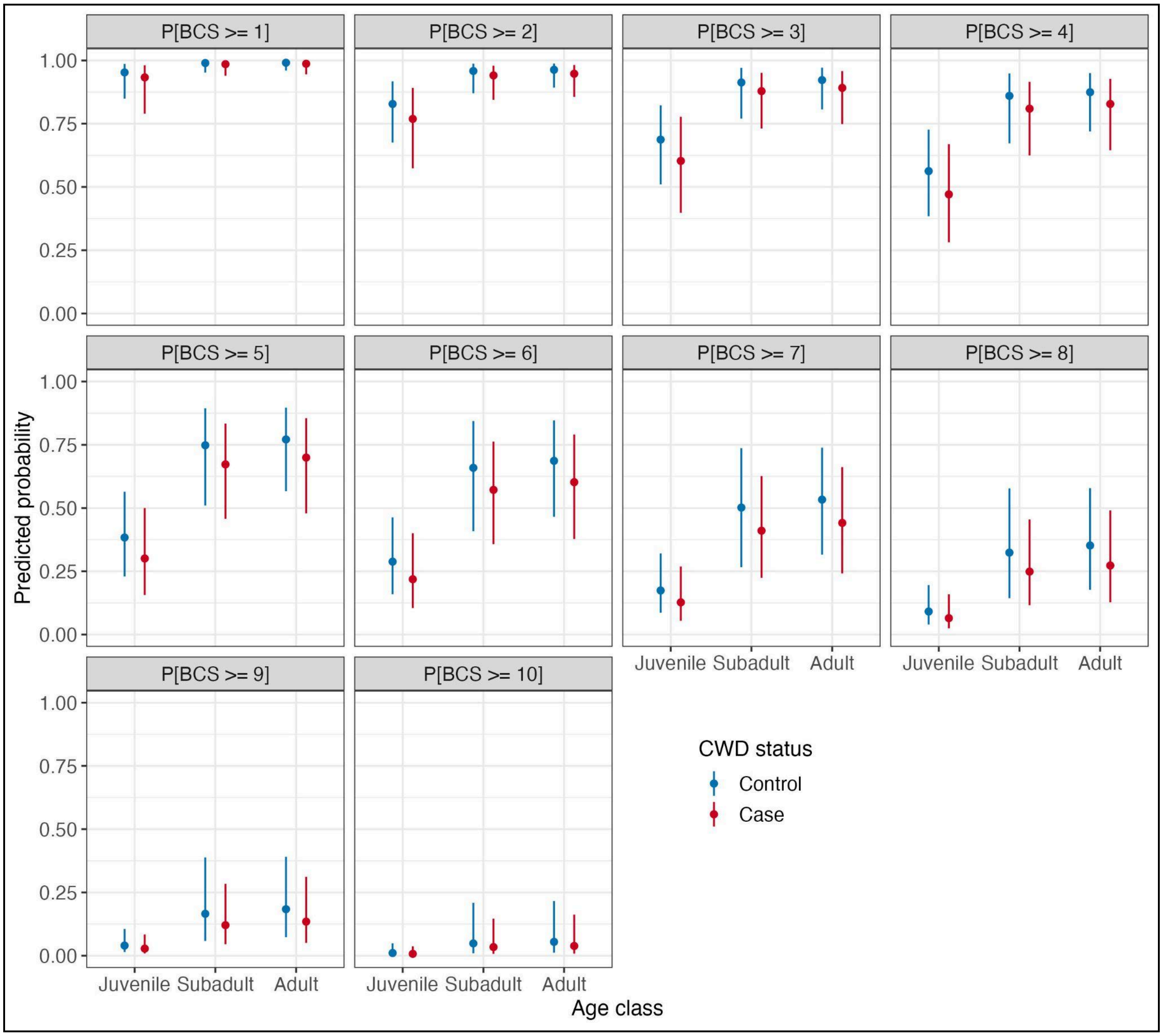

Figure S5: *Predicted effects from cumulative logistic regression for body condition score at capture among white-tailed deer (*Odocoileus virginianus*) in southwest Wisconsin, USA. Body condition scores (BCS) were integers ranging from 0 (low) to 10 (high). Each panel gives the predicted probability of a deer being classed as a given BCS or greater (e.g., the probability of being classed as a BCS of 1 or more is very high). Points represent probability estimates, and vertical colored lines the 95% confidence intervals. Colors represent chronic wasting disease (CWD) status.*

Table S3: Candidate logistic regressions for the occurrence of an excursion among white-tailed deer (*Odocoileus virginianus*) in southwest Wisconsin, USA

**Model**	**ΔAIC**	**Binned residuals**
CWD status + sex + days observed	0.0	Fail
**CWD status + sex + days observed + hours between fixes**	**0.3**	**Pass**
CWD status*sex + days observed + hours between fixes	1.3	Fail
CWD status + sex + days observed^2	1.4	Pass
CWD status + sex + hours between fixes	5.0	NA

Note: CWD = Chronic wasting disease; AIC = Akaike information criterion. Days observed and hours between fixes were scaled and centered. The top model is highlighted in bold. Pass/fail for binned residuals is based on the expectation that about 95% of binned residuals would fall within the error bounds if a model is correctly specified. Binned residuals were only evaluated for models within Δ2 AIC of the "top" model.

Table S4: Logistic regression results for occurrence of an excursion at any time during the six month observation window of white-tailed deer (*Odocoileus virginianus*) in southwest Wisconsin, USA

**Predictor**	**Odds Ratio**	**95% CI**
**Intercept**	**2.19**	**1.06 – 4.80**
CWD status: case	0.93	0.34 – 2.52
Sex: male	0.85	0.21 – 3.89
**Number of days observed (scaled and centered)**	**1.91**	**1.17 – 3.25**
Hour between fixes (scaled and centered)	1.47	0.82 – 2.66

Note: CWD = chronic wasting disease; 95% CI = 95% confidence interval. Statistically significant predictors are highlighted in bold.

Table S5: Candidate generalized linear mixed models for the monthly occurrence of an excursion among white-tailed deer (*Odocoileus virginianus*) in southwest Wisconsin, USA

**Model**	**ΔAIC**
**CWD status*month + sex + days observed^2**	**0.0**
CWD status*month pre-death + sex + days observed	2.6
CWD status*month pre-death + days observed + hours between fixes	2.6

Note: CWD = Chronic wasting disease; AIC = Akaike information criterion. Days observed and hours between fixes were scaled and centered. All models included individual as a random intercept. The top model is highlighted in bold.

Table S6: Generalized linear mixed model results for monthly occurrence of an excursion among white-tailed deer (*Odocoileus virginianus*) in southwest Wisconsin, USA

**Predictor**	**Odds Ratio**	**95% CI**
Intercept	0.81	0.36 – 1.81
CWD status: case	0.48	0.17 – 1.37
**Months prior to case death**	**0.75**	**0.60 – 0.94**
Sex: male	1.22	0.60 – 2.47
Number of days observed 1º (scaled and centered)	0.82	0.48 – 1.40
Number of days observed 2º (scaled and centered)	0.74	0.54 – 1.01
Case : months prior to case death	1.32	0.98 – 1.77
**Random effects**	**Variance**	**Standard deviation**
Individual	0.23	0.48

Note: CWD = chronic wasting disease; 95% CI = 95% confidence interval. Statistically significant predictors are highlighted in bold text. Results correspond to those pictured in Figure 4 in the main text.

Table S7: Candidate linear mixed models for the log-transformed excursion distance among white-tailed deer (*Odocoileus virginianus*) in southwest Wisconsin, USA

Model	ΔAIC
**CWD status + month pre-death + sex**	**0.0**
CWD status*month pre-death + sex	1.2
CWD status + month pre-death*sex	1.8
CWD status*sex + month pre-death	1.9
CWD status + month pre-death + sex + season	2.6
CWD status*month pre-death + sex + season	3.8
CWD status*month pre-death + sex*season	7.7

Note: CWD = Chronic wasting disease; AIC = Akaike information criterion. All models included individual as a random intercept. The top model is highlighted in bold.

Table S8: Semi-parameteric interval survival model results for the duration of excursions completed by white-tailed deer (*Odocoileus virginianus*) in southwest Wisconsin, USA

Predictor	Estimate	Hazard ratio	Standard error	p-value
CWD status: case	0.03	1.03	0.23	0.88
Hours between fixes (scaled and centered)	0.08	1.08	0.11	0.47

Note: CWD = chronic wasting disease.

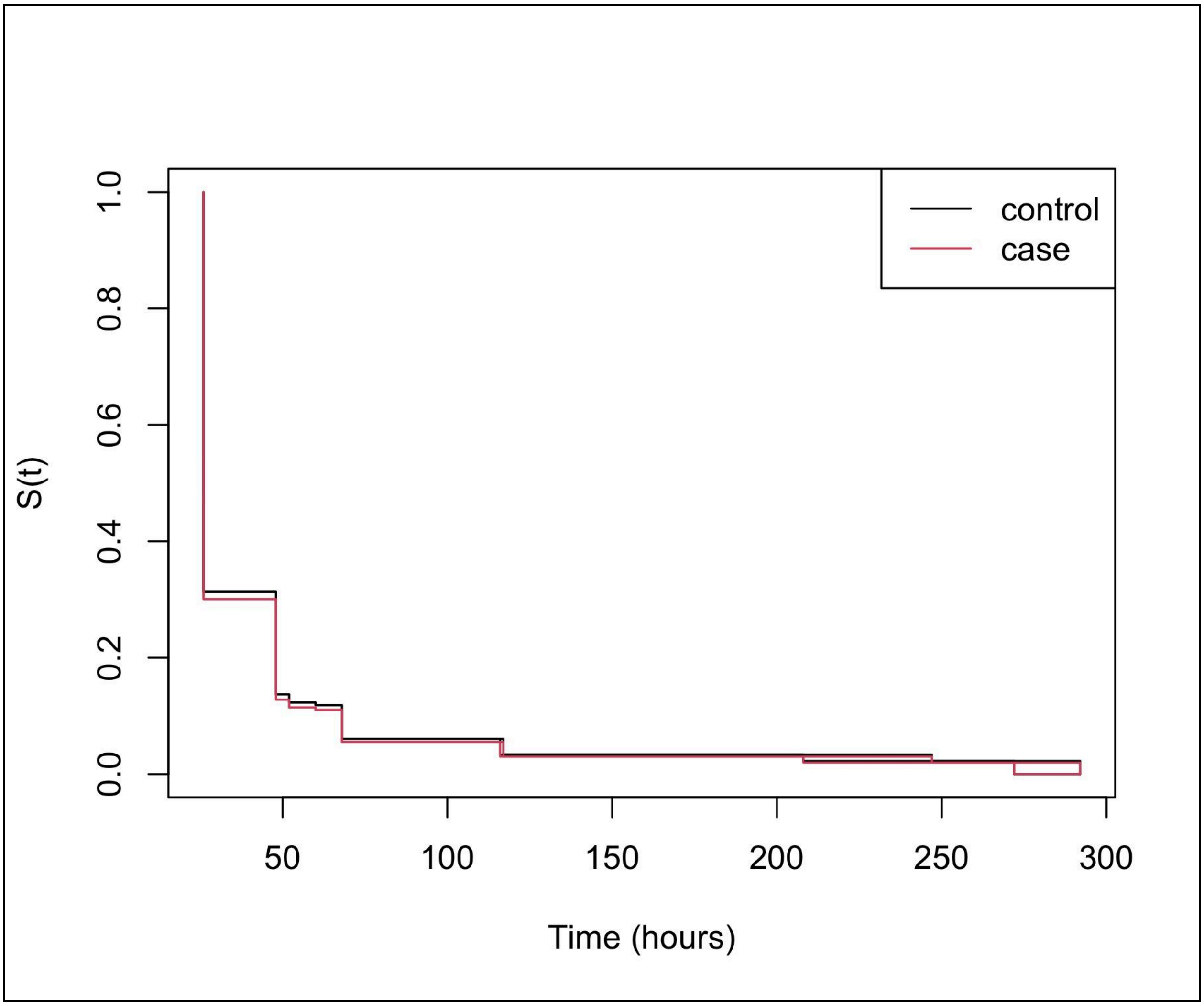

Figure S6: *Semi-parameteric interval survival curve for the duration of excursions completed by white-tailed deer (Odocoileus virginianus) in southwest Wisconsin, USA. S(t) corresponds to the probability of an excursion lasting a specified time. Results correspond to those given in Appendix S2: Table S5. Colors of lines represent chronic wasting disease status, with cases given in red and controls in black.*

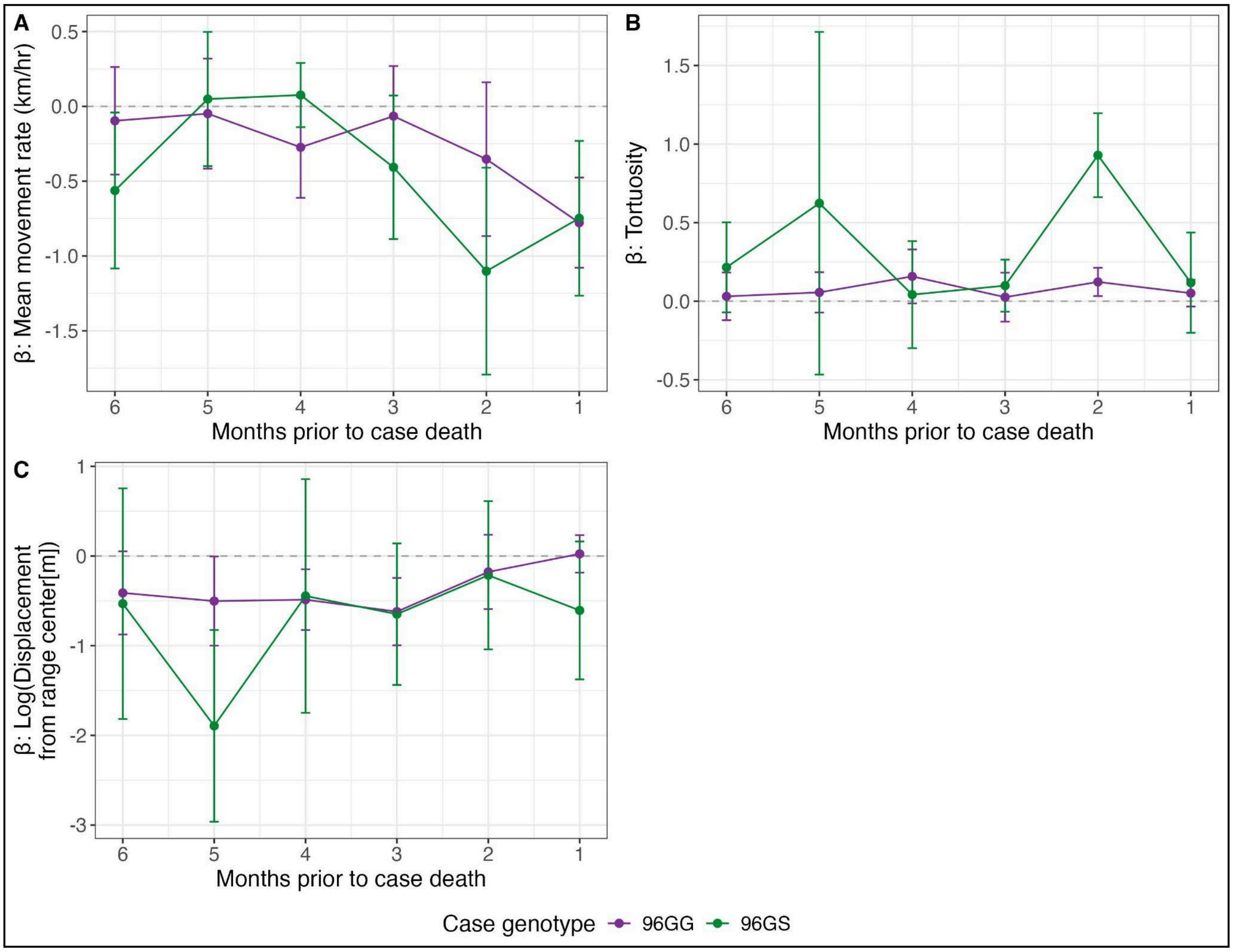

Figure S7: *The relationship between key movement characteristics and chronic wasting disease (CWD) status and host genotype for white-tailed deer (*Odocoileus virginianus*) in southwest Wisconsin, USA, as quantified by conditional logistic regression. The response variable was CWD case or control status, so the y-axis gives parameter estimates for the effect of each movement characteristic (β) on CWD status. A separate model was fit for each month prior to case death, so points represent the estimate from a given model, and bars are the 95% confidence intervals. Models were also fit separately based on the genotype of cases at* PRNP *codon 96 (given by point and line color). An estimate of zero indicates no relationship between a metric and CWD status; a negative value indicates case status was associated with lower metric values than controls.*

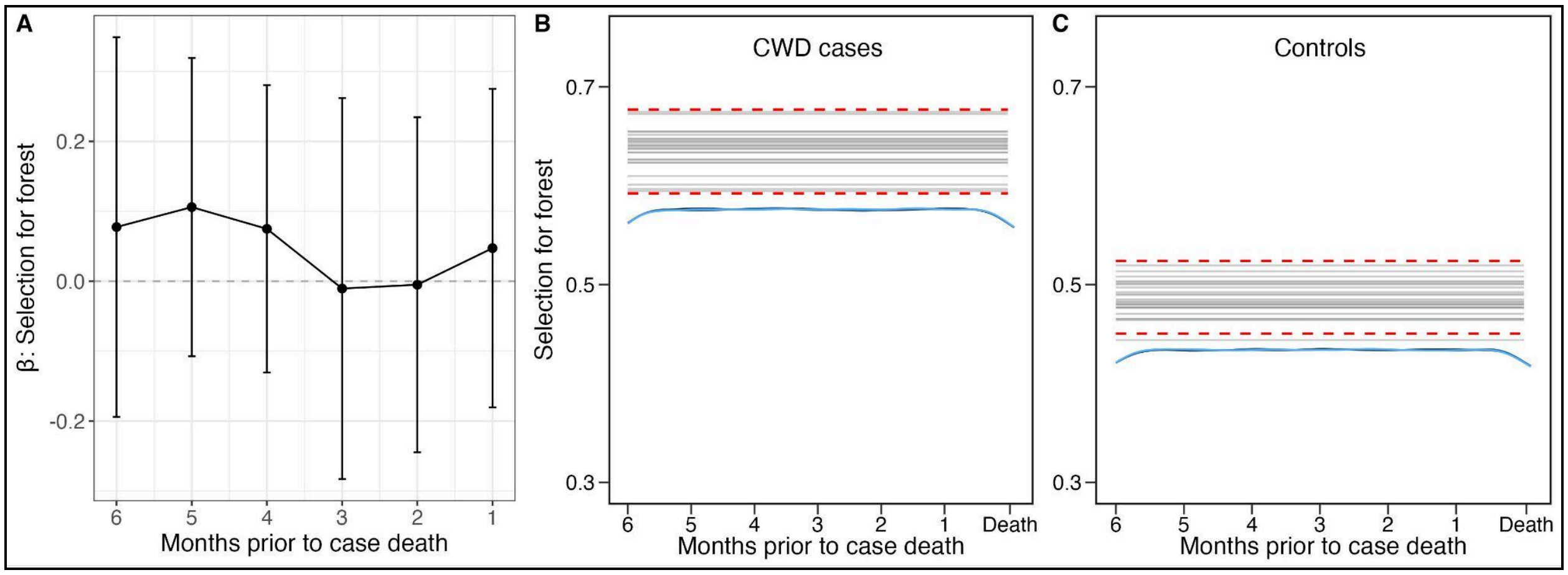

Figure S8: *The relationship between selection for forested habitat and chronic wasting disease (CWD) status for white-tailed deer (Odocoileus virginianus) in southwest Wisconsin, USA. A) conditional logistic regression results, such that the y-axis gives parameter estimates (points) and 95% confidence intervals (vertical bars) for the effect of selection for forest (β) on CWD status. A negative estimate indicates that case status was associated with reduced relative selection for that habitat characteristic, as compared to controls. B) and C) Bayesian change points for CWD cases (B) and controls (C) in selection for forested habitat. Blue lines in each plot show posterior model draws for the change point of a given metric. Solid gray lines give posterior draws for the fitted metric values, and red dashed lines the 95% quantiles. In these plots, zero indicates "no selection or avoidance," so positive fitted values indicate relative selection for forested habitat.*

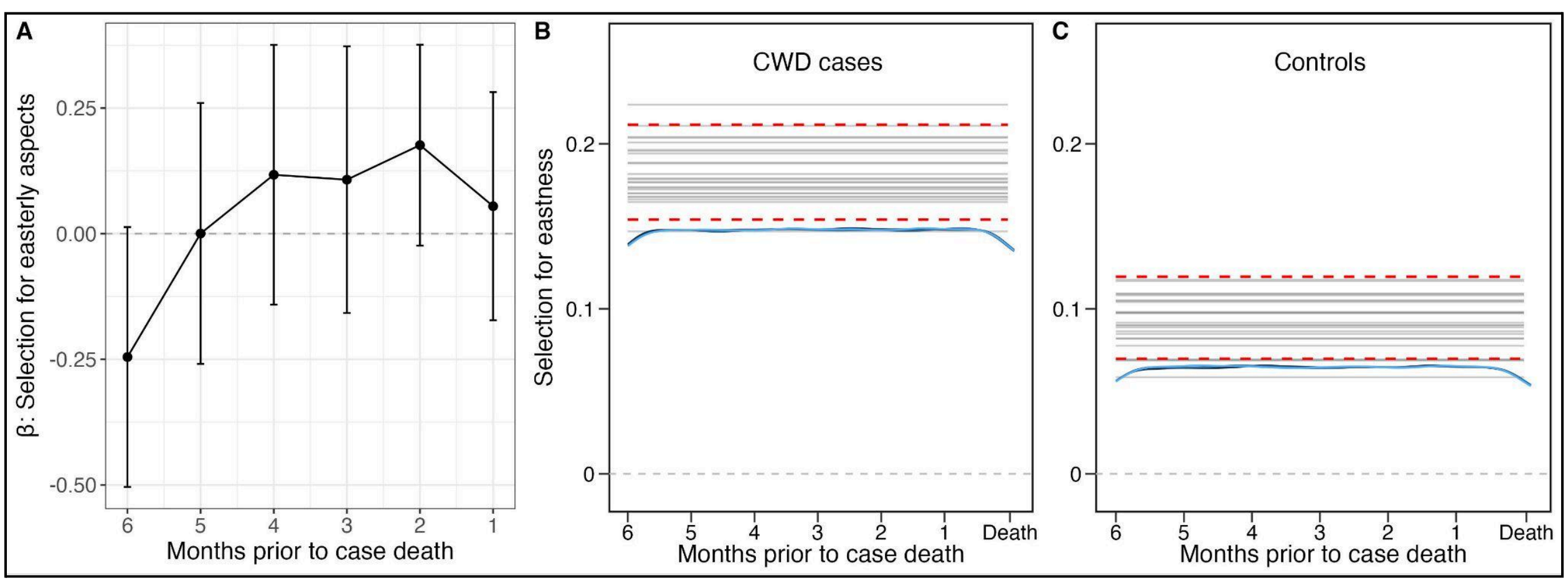

Figure S9: *The relationship between selection for easterly aspects and chronic wasting disease (CWD) status for white-tailed deer (Odocoileus virginianus) in southwest Wisconsin, USA. A) conditional logistic regression results, such that the y-axis gives parameter estimates (points) and 95% confidence intervals (vertical bars) for the effect of selection for easterly aspects (β) on CWD status. A negative estimate indicates that case status was associated with reduced relative selection for easterly aspects, as compared to controls. B) and C) Bayesian change points for*

CWD cases (B) and controls (C) in selection for easterly aspects. Blue lines in each plot show posterior model draws for the change point of a given metric. Solid gray lines give posterior draws for the fitted metric values, and red dashed lines the 95% quantiles. In these plots, zero indicates "no selection or avoidance," so positive fitted values indicate relative selection for easterly aspects.

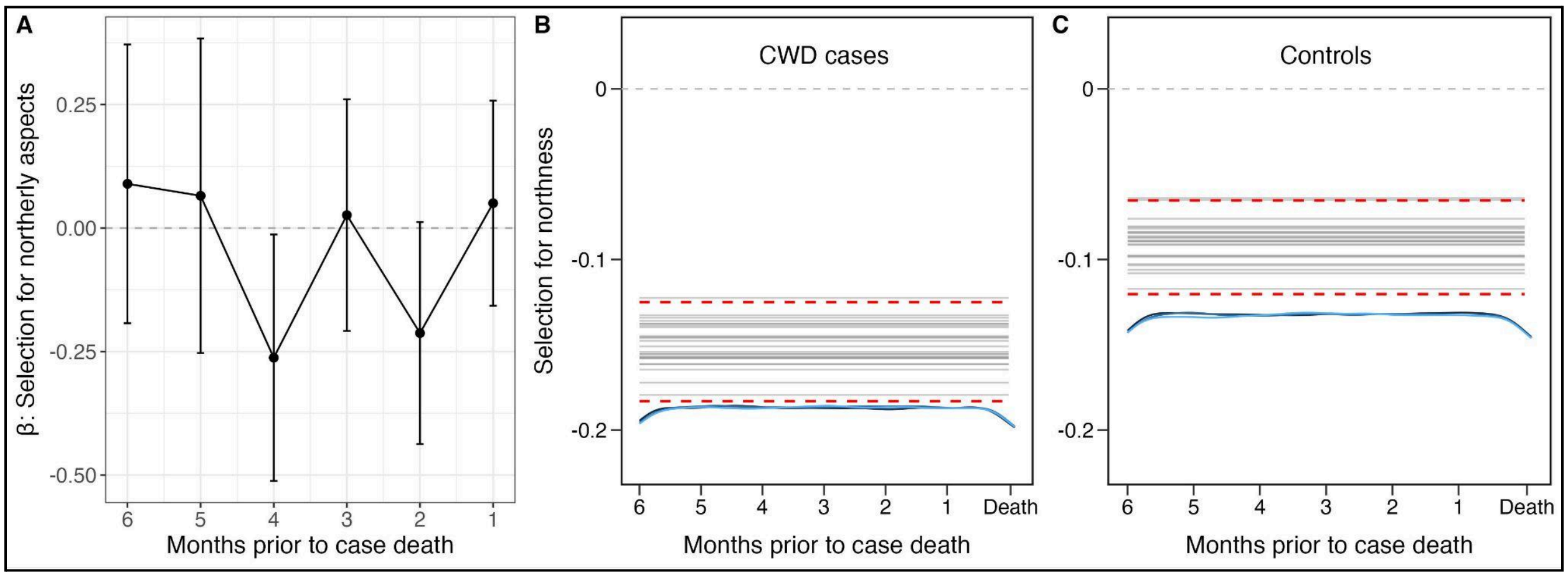

Figure S10: *The relationship between selection for northerly aspects and chronic wasting disease (CWD) status for white-tailed deer (Odocoileus virginianus) in southwest Wisconsin, USA. A) conditional logistic regression results, such that the y-axis gives parameter estimates (points) and 95% confidence intervals (vertical bars) for the effect of selection for northerly aspects (β) on CWD status. A negative estimate indicates that case status was associated with reduced relative selection for northerly aspects, as compared to controls. B) and C) Bayesian change points for CWD cases (B) and controls (C) in selection for northerly aspects. Blue lines in each plot show posterior model draws for the change point of a given metric. Solid gray lines give posterior draws for the fitted metric values, and red dashed lines the 95% quantiles. In these plots, zero indicates "no selection or avoidance," so negative fitted values indicate relative avoidance of northerly aspects.*

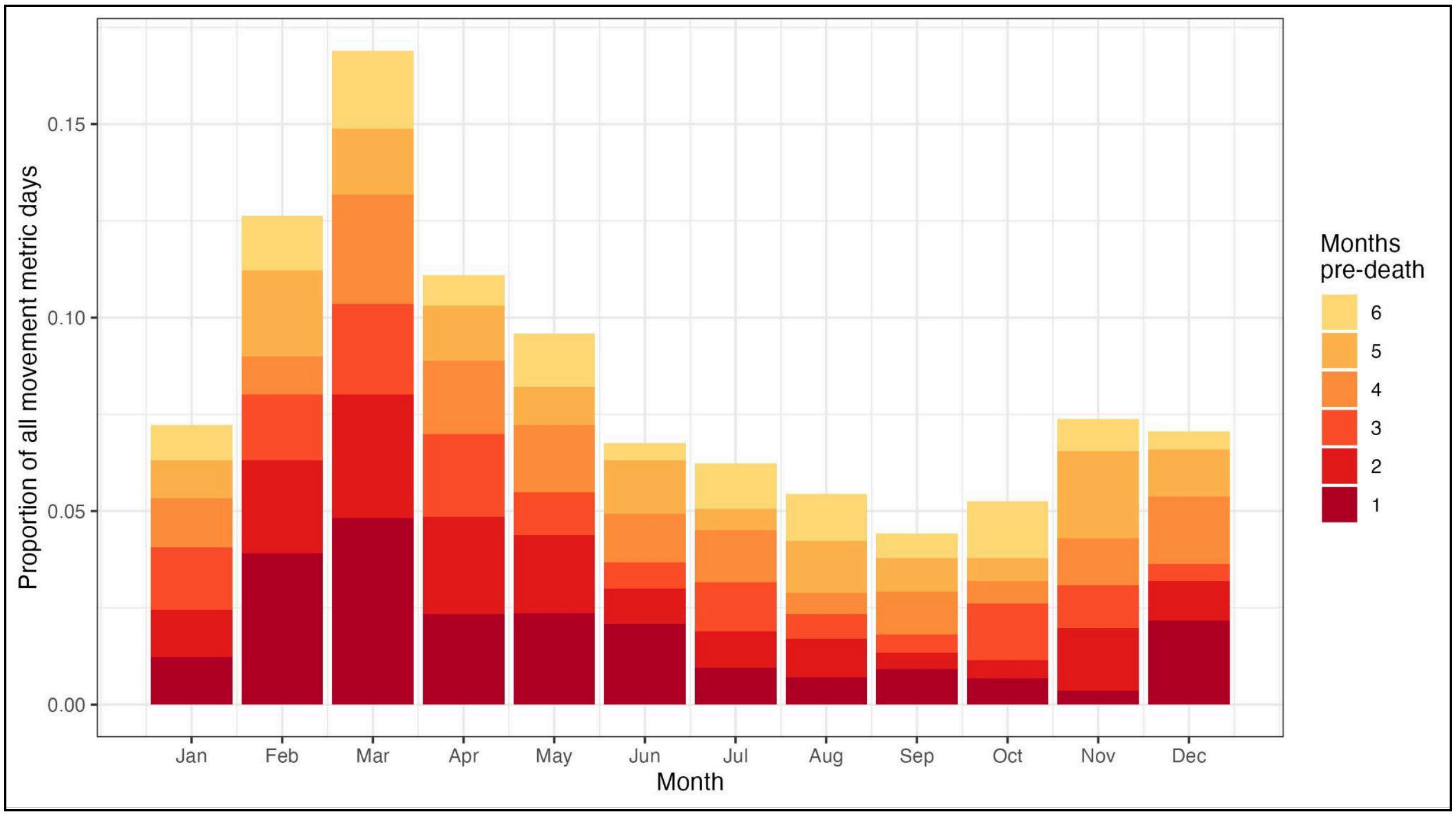

Figure S11: *Distribution of movement metric observations for white-tailed deer (*Odocoileus virginianus*) that died in end-stage chronic wasting disease in southwest Wisconsin, USA. Most chronic wasting disease mortalities in this study occurred in January through May, so the preponderance of "late stage" movement data (especially from 1-2 months prior to death) was from late winter to early spring.*
